# Supplementary material for: A Multifield Study on Dimethyl Acetylenedicarboxylate: A Reagent Able to Build a New Cycle on Diaminoimidazoles
Source: Molecules. 2022 May 22;27(10):3326. doi: 10.3390/molecules27103326 (PMC9147549; doi:10.3390/molecules27103326)
Supplement: Supplementary file 1 [file molecules-27-03326-s001.zip › molecules-1717117-supplementary.pdf]

Supporting Information

AN EXPERIMENTAL AND THEORETICAL STUDY OF THE INTERACTION OF DIAMINOIMIDAZOLES WITH DIMETHYL ACETYLENEDICARBOXYLATE

Dmitrii Yu. Vandyshev *, Oleg N. Burov, Anton V. Lisovin, Daria A. Mangusheva, Mikhail A. Potapov, Tatiana N. Ilyinova, Khidmet S. Shikhaliev

Content

Discussion of the results of quantum chemical calculations when alcohol molecule was included in the processes shown in Schemes S1 and S2...................... S1
Results of HPLC-MS analysis in assessing the conversion of the studied process................. S3
Spectral data of the obtained compounds S17
DFT calculation S41

Discussion of the results of quantum chemical calculations when alcohol molecules were included in the processes shown in Schemes S1 and S2

When alcohol molecules were included in the formation of covalent adduct **59** in **Scheme 11**, a similar picture was observed (**Scheme S1**). The alcohol molecule is coordinated in zwitterionic associate **65** with a negatively charged double bond carbon atom. Structure **65** was 38.5 kcal/mol higher in energy than the initial reagents (**Figure S1**). As in the previous cases, we observe the destabilization of the zwitterionic complex in comparison with a similar bimolecular processes. Associate **65** is 5.1 kcal/mol higher in energy than structure **58**.

Scheme S1.

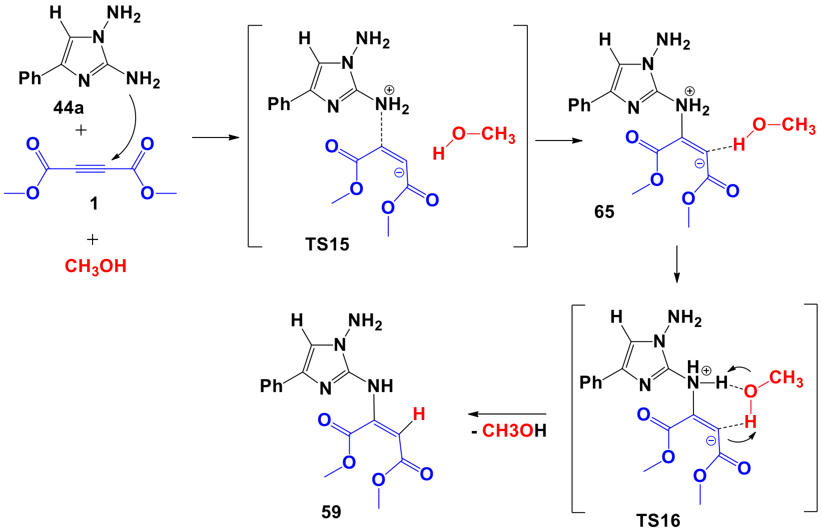

The inclusion of an alcohol molecule at the molecular level did not affect the possibility of further cyclization, and the covalent product is still the final stage of the studied process.

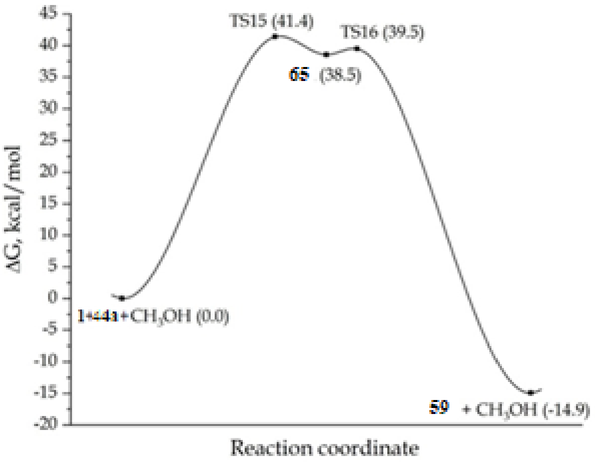

Figure S1. The MEP for the trimolecular reaction of the formation of adduct **59**. The total energy of the starting reagents is taken as the reference point.

The transition from a bimolecular process in **Scheme 12** to a trimolecular process involving an alcohol molecule in **Scheme S2** has a tendency similar to the three previously described cases. In particular, the formation of zwitterionic complex **66** becomes less advantageous in comparison with the bimolecular process (**Figure S2, Figure 6**.), and the proton transfer during the formation of covalent adduct **61** from structure **66** becomes barrier-free with the involvement of alcohol molecules.

Scheme S2.

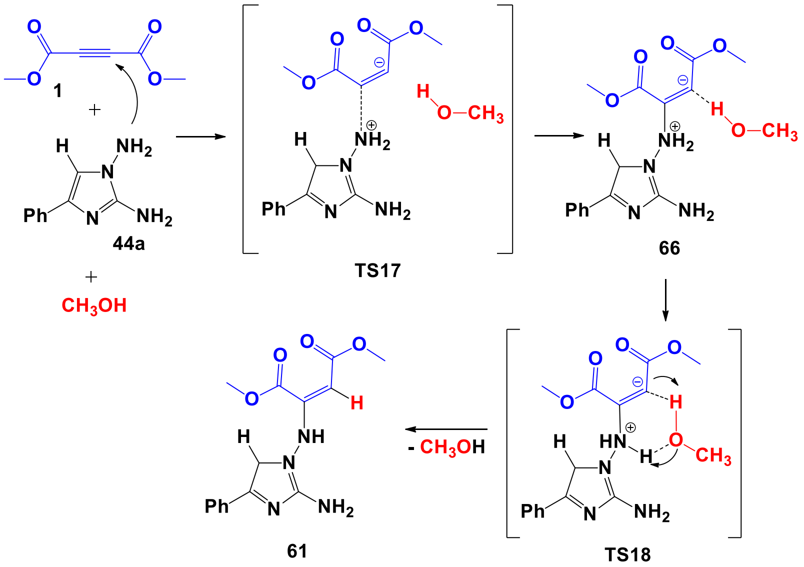

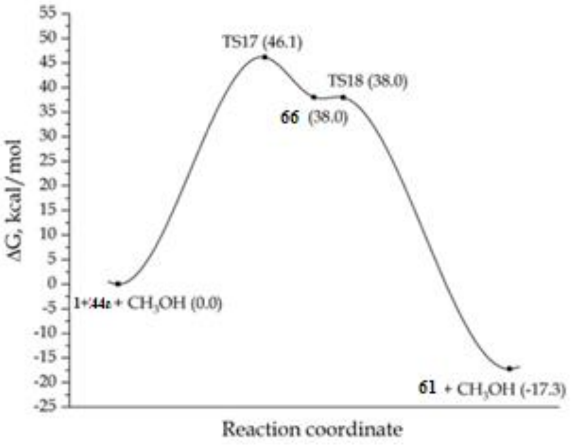

Figure S2. MEP for the trimolecular reaction of the formation of product **61**.The total energy of the starting reagents was taken as the reference point.

Results of HPLC-MS analysis in assessing the conversion of the studied process

Table S1. Start of the reaction 0 minutes (independent of the solvent).

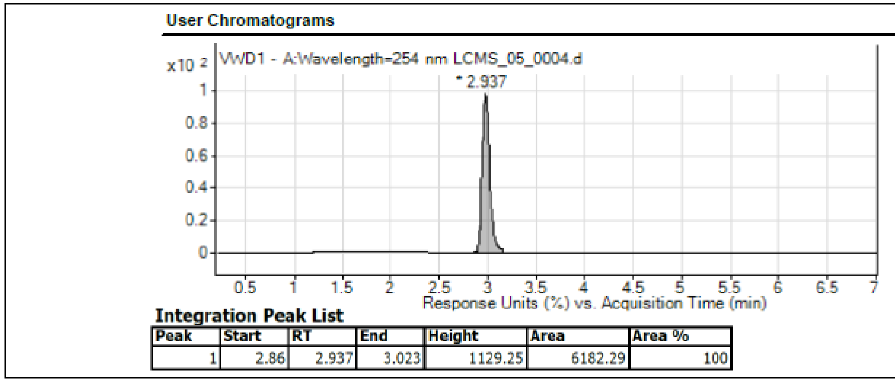

Integration Peak List

Peak	Start	RT	End	Height	Area	Area %
1	2.86	2.937	3.023	1129.25	6182.29	100

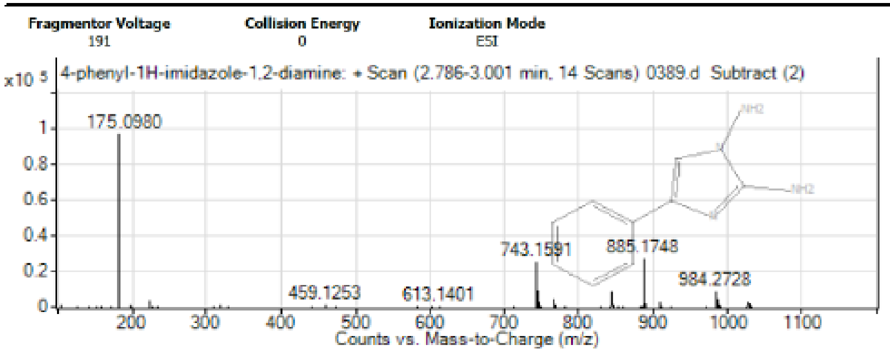

Table S2. Conducting the reaction in benzene.

60 minutes

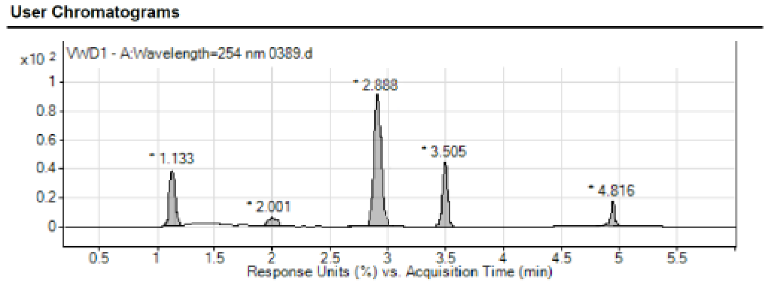

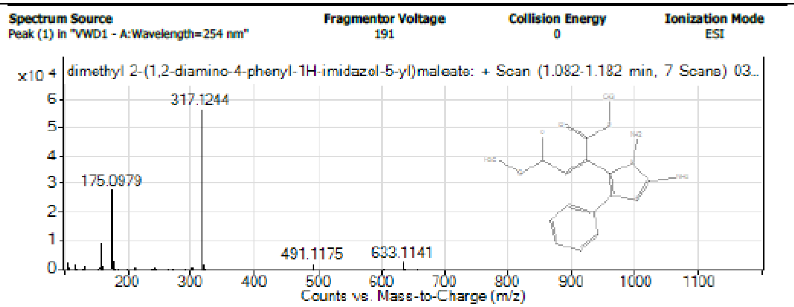

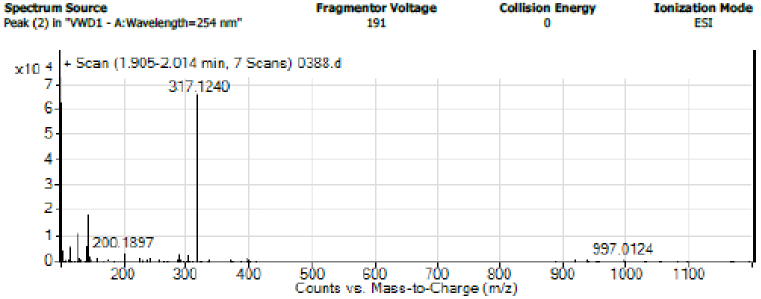

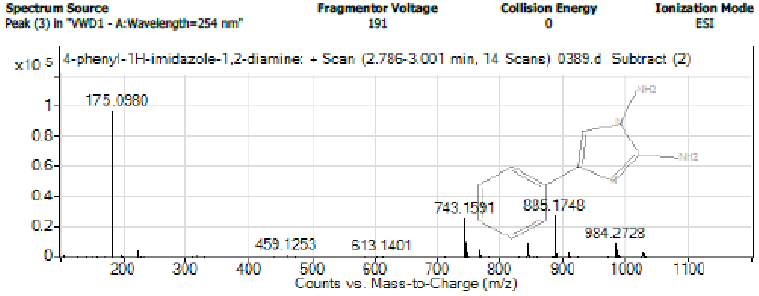

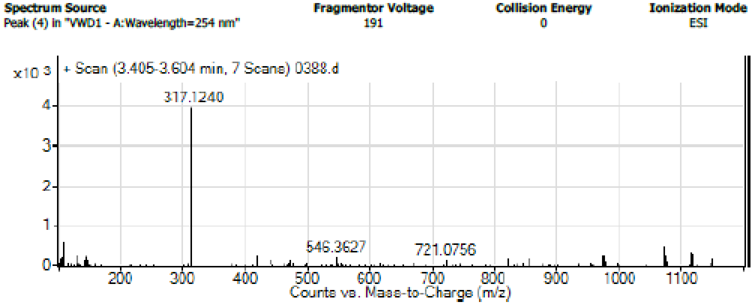
Spectrum Source
Peak (4) in "VWD1 - A:Wavelength=254 nm"
Fragmentor Voltage
191
Collision Energy
0
Ionization Mode
ESI
x10 3
+ Scan (3.405-3.604 min, 7 Scans) 0388.d
317.1240
546.3627
721.0756
Counts vs. Mass-to-Charge (m/z)

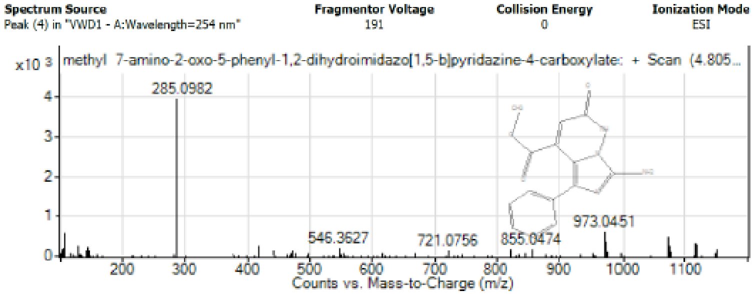
Spectrum Source
Peak (4) in "VWD1 - A:Wavelength=254 nm"
Fragmentor Voltage
191
Collision Energy
0
Ionization Mode
ESI
x10 3
methyl 7-amino-2-oxo-5-phenyl-1,2-dihydroimidazo[1,5-b]pyridazine-4-carboxylate: + Scan (4.805...
285.0982
546.3627
721.0756
855.0474
973.0451
Counts vs. Mass-to-Charge (m/z)

120 minutes

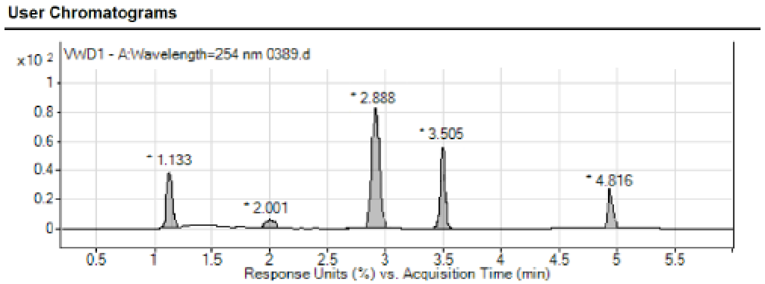
User Chromatograms
x10 2
VWD1 - A:Wavelength=254 nm 0389.d
1.133
2.001
2.888
3.505
4.816
Response Units (%) vs. Acquisition Time (min)

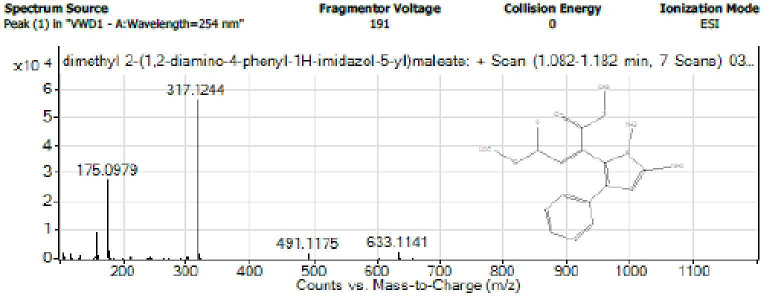
Spectrum Source
Peak (1) in "VWD1 - A:Wavelength=254 nm"
Fragmentor Voltage
191
Collision Energy
0
Ionization Mode
ESI
x10 4
dimethyl 2-(1,2-diamino-4-phenyl-1H-imidazol-5-yl)maleate: + Scan (1.082-1.182 min, 7 Scans) 03...
317.1244
175.0979
491.1175
633.1141
Counts vs. Mass-to-Charge (m/z)

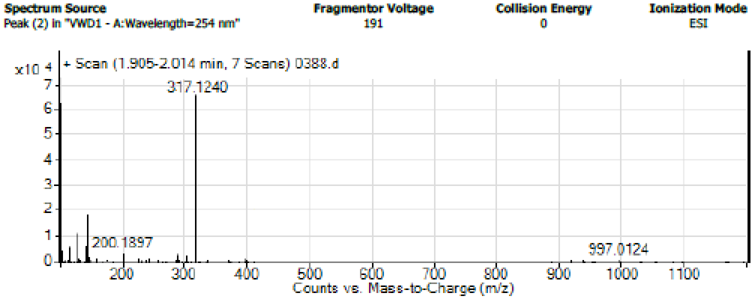
Spectrum Source
Peak (2) in "VWD1 - A:Wavelength=254 nm"
Fragmentor Voltage
191
Collision Energy
0
Ionization Mode
ESI
x10 4
+ Scan (1.905-2.014 min, 7 Scans) 0388.d
317.1240
200.1897
997.0124
Counts vs. Mass-to-Charge (m/z)

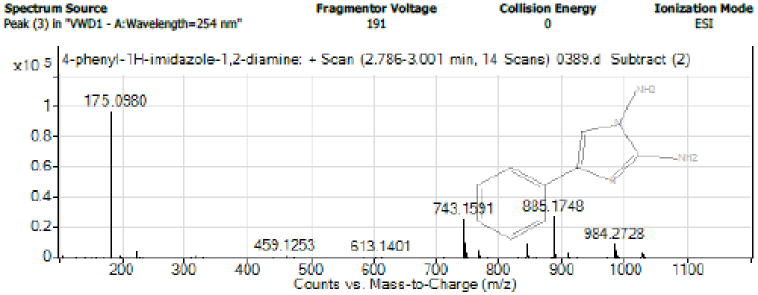
Spectrum Source
Peak (3) in "VWD1 - A:Wavelength=254 nm"
Fragmentor Voltage
191
Collision Energy
0
Ionization Mode
ESI
x10 5
4-phenyl-1H-imidazole-1,2-diamine: + Scan (2.786-3.001 min, 14 Scans) 0389.d Subtract (2)
175.0980
459.1253
613.1401
743.1591
885.1748
984.2728
Counts vs. Mass-to-Charge (m/z)

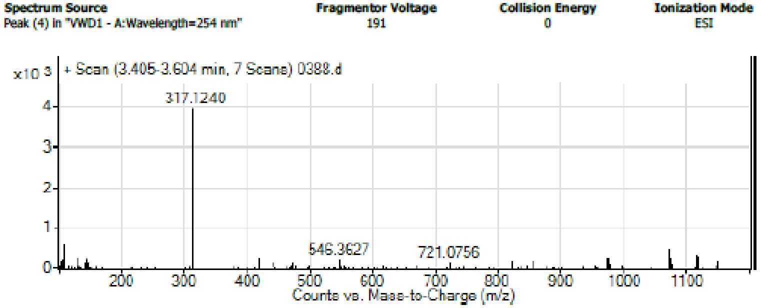
Spectrum Source
Peak (4) in "VWD1 - A:Wavelength=254 nm"
Fragmentor Voltage
191
Collision Energy
0
Ionization Mode
ESI
x10 3
+ Scan (3.405-3.604 min, 7 Scans) 0388.d
317.1240
546.3627
721.0756
Counts vs. Mass-to-Charge (m/z)

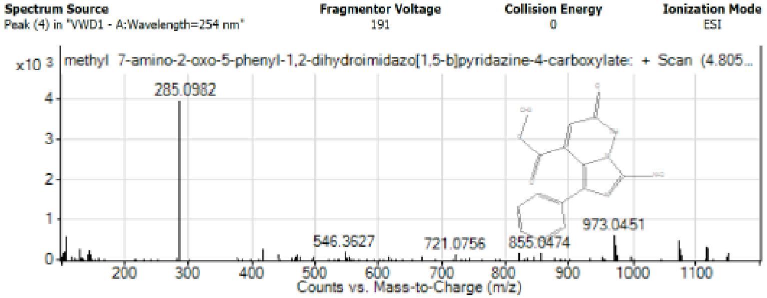
Spectrum Source
Peak (4) in "VWD1 - A:Wavelength=254 nm"
Fragmentor Voltage
191
Collision Energy
0
Ionization Mode
ESI
x10 3
methyl 7-amino-2-oxo-5-phenyl-1,2-dihydroimidazo[1,5-b]pyridazine-4-carboxylate: + Scan (4.805...
285.0982
546.3627
721.0756
855.0474
973.0451
Counts vs. Mass-to-Charge (m/z)

Table S3. Conducting the reaction in 1,4-dioxane.

<table>
<tr>
<td>60 minutes</td>
<td>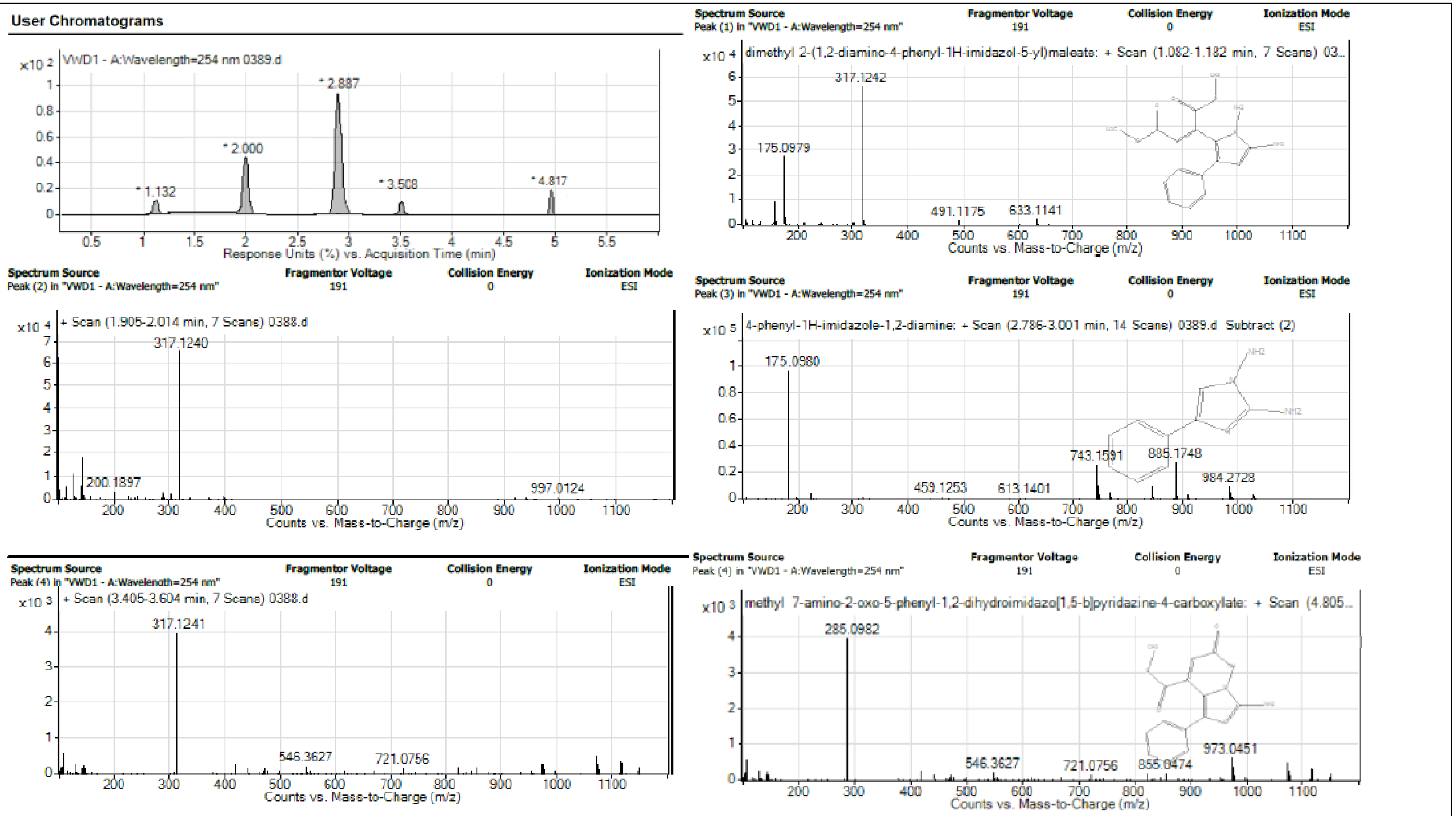
</td>
</tr>
</table>

120 minutes

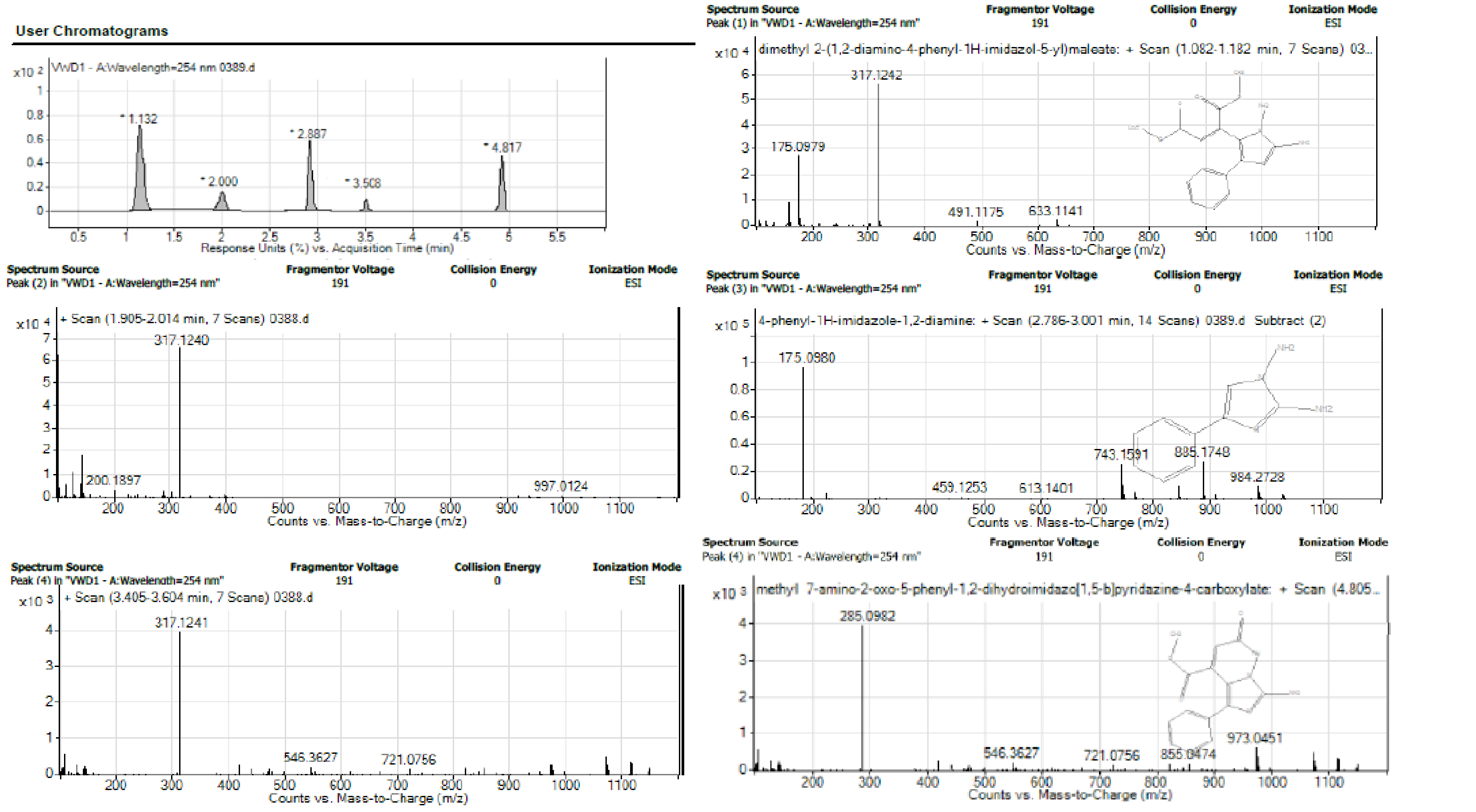
User Chromatograms
VWD1 - A:Wavelength=254 nm 0389.d
1.132
2.000
2.887
3.508
4.817
Response Units (%) vs. Acquisition Time (min)
Spectrum Source
Peak (1) in "VWD1 - A:Wavelength=254 nm"
Fragmentor Voltage 191
Collision Energy 0
Ionization Mode ESI
dimethyl 2-(1,2-diamino-4-phenyl-1H-imidazol-5-yl)maleate: + Scan (1.082-1.182 min, 7 Scans) 03...
317.1242
175.0979
491.1175
633.1141
Counts vs. Mass-to-Charge (m/z)
Spectrum Source
Peak (2) in "VWD1 - A:Wavelength=254 nm"
Fragmentor Voltage 191
Collision Energy 0
Ionization Mode ESI
+ Scan (1.905-2.014 min, 7 Scans) 0388.d
317.1240
200.1897
997.0124
Counts vs. Mass-to-Charge (m/z)
Spectrum Source
Peak (3) in "VWD1 - A:Wavelength=254 nm"
Fragmentor Voltage 191
Collision Energy 0
Ionization Mode ESI
4-phenyl-1H-imidazole-1,2-diamine: + Scan (2.786-3.001 min, 14 Scans) 0389.d Subtract (2)
175.0980
743.1591
885.1748
459.1253
613.1401
984.2728
Counts vs. Mass-to-Charge (m/z)
Spectrum Source
Peak (4) in "VWD1 - A:Wavelength=254 nm"
Fragmentor Voltage 191
Collision Energy 0
Ionization Mode ESI
+ Scan (3.405-3.604 min, 7 Scans) 0388.d
317.1241
546.3627
721.0756
Counts vs. Mass-to-Charge (m/z)
Spectrum Source
Peak (4) in "VWD1 - A:Wavelength=254 nm"
Fragmentor Voltage 191
Collision Energy 0
Ionization Mode ESI
methyl 7-amino-2-oxo-5-phenyl-1,2-dihydroimidazo[1,5-b]pyridazine-4-carboxylate: + Scan (4.805...
285.0982
546.3627
721.0756
855.0474
973.0451
Counts vs. Mass-to-Charge (m/z)

Table S4. Conducting the reaction in methylene chloride.

60 minutes

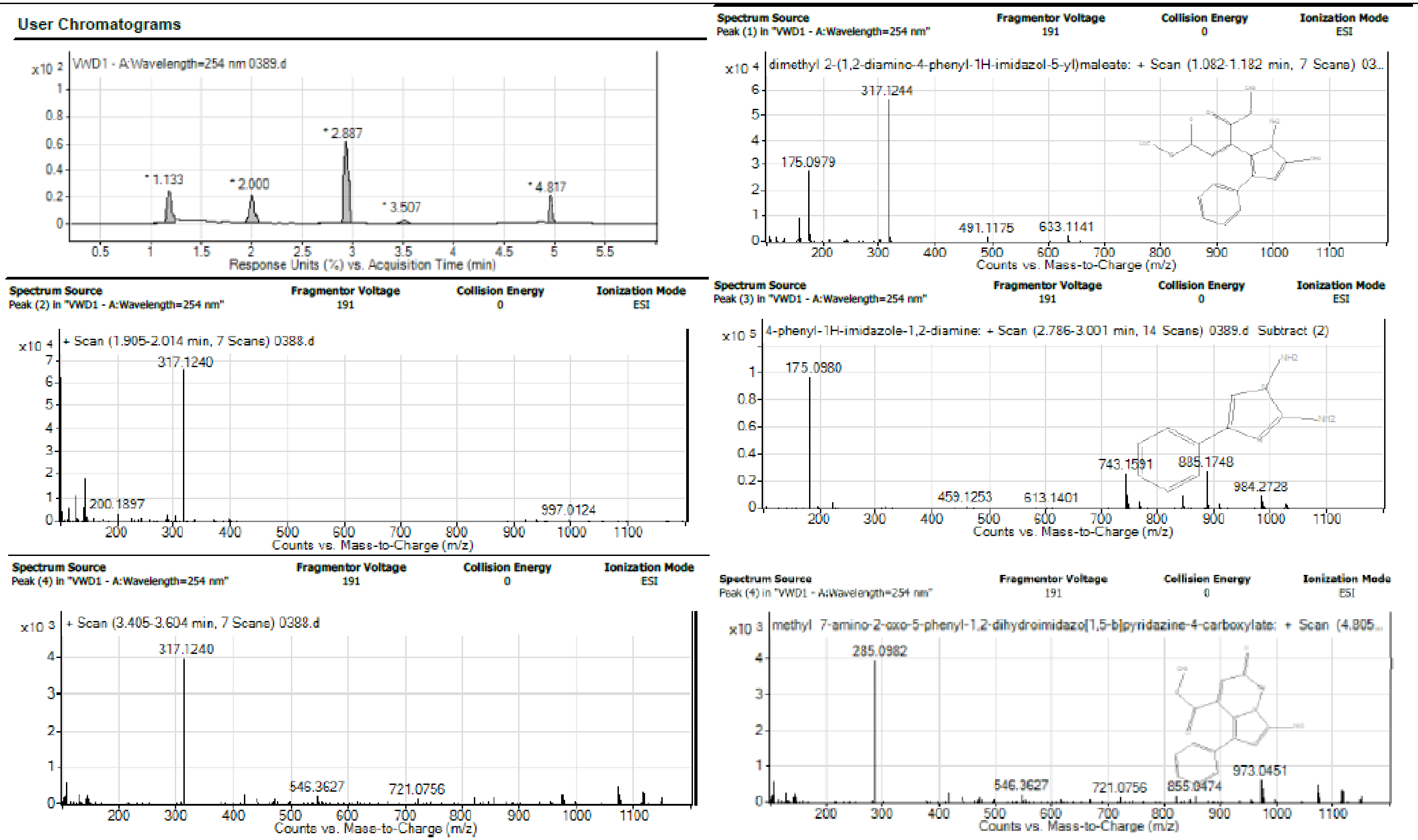

120 minutes	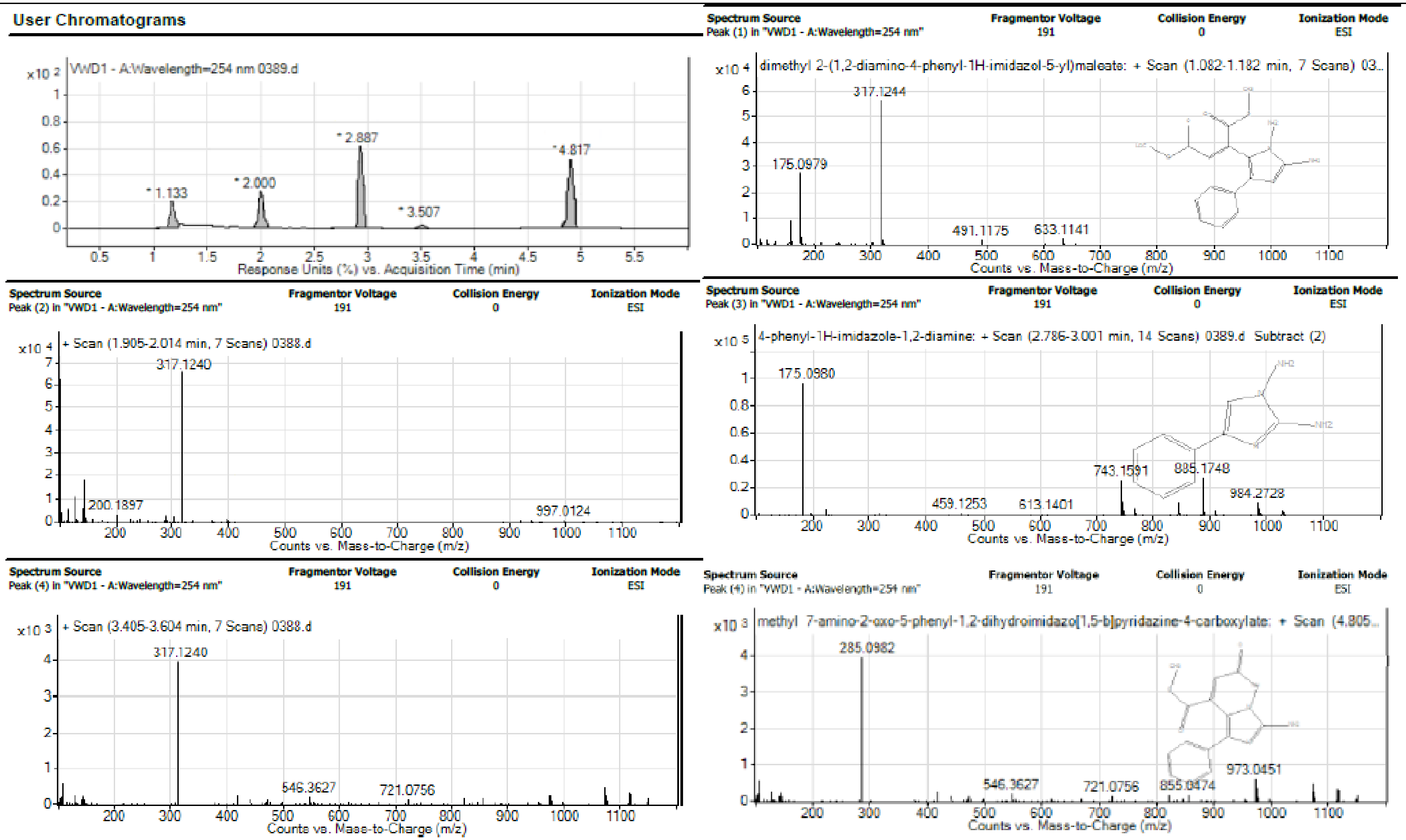

User Chromatograms
x10 2 VWD1 - A:Wavelength=254 nm 0389.d
* 1.133
* 2.000
* 2.887
* 3.507
* 4.817
Response Units (%) vs. Acquisition Time (min)
Spectrum Source
Peak (1) in "VWD1 - A:Wavelength=254 nm"
Fragmentor Voltage 191
Collision Energy 0
Ionization Mode ESI
x10 4 dimethyl 2-(1,2-diamino-4-phenyl-1H-imidazol-5-yl)maleate: + Scan (1.082-1.182 min, 7 Scans) 03...
317.1244
175.0979
491.1175
633.1141
Counts vs. Mass-to-Charge (m/z)
Spectrum Source
Peak (2) in "VWD1 - A:Wavelength=254 nm"
Fragmentor Voltage 191
Collision Energy 0
Ionization Mode ESI
x10 4 + Scan (1.905-2.014 min, 7 Scans) 0388.d
317.1240
200.1897
997.0124
Counts vs. Mass-to-Charge (m/z)
Spectrum Source
Peak (3) in "VWD1 - A:Wavelength=254 nm"
Fragmentor Voltage 191
Collision Energy 0
Ionization Mode ESI
x10 5 4-phenyl-1H-imidazole-1,2-diamine: + Scan (2.786-3.001 min, 14 Scans) 0389.d Subtract (2)
175.0980
743.1591
885.1748
984.2728
459.1253
613.1401
Counts vs. Mass-to-Charge (m/z)
Spectrum Source
Peak (4) in "VWD1 - A:Wavelength=254 nm"
Fragmentor Voltage 191
Collision Energy 0
Ionization Mode ESI
x10 3 + Scan (3.405-3.604 min, 7 Scans) 0388.d
317.1240
546.3627
721.0756
Counts vs. Mass-to-Charge (m/z)
Spectrum Source
Peak (4) in "VWD1 - A:Wavelength=254 nm"
Fragmentor Voltage 191
Collision Energy 0
Ionization Mode ESI
x10 3 methyl 7-amino-2-oxo-5-phenyl-1,2-dihydroimidazo[1,5-b]pyridazine-4-carboxylate: + Scan (4.805...
285.0982
973.0451
546.3627
721.0756
855.0474
Counts vs. Mass-to-Charge (m/z)

Table S5. Conducting the reaction in chloroform.

**60 minutes**	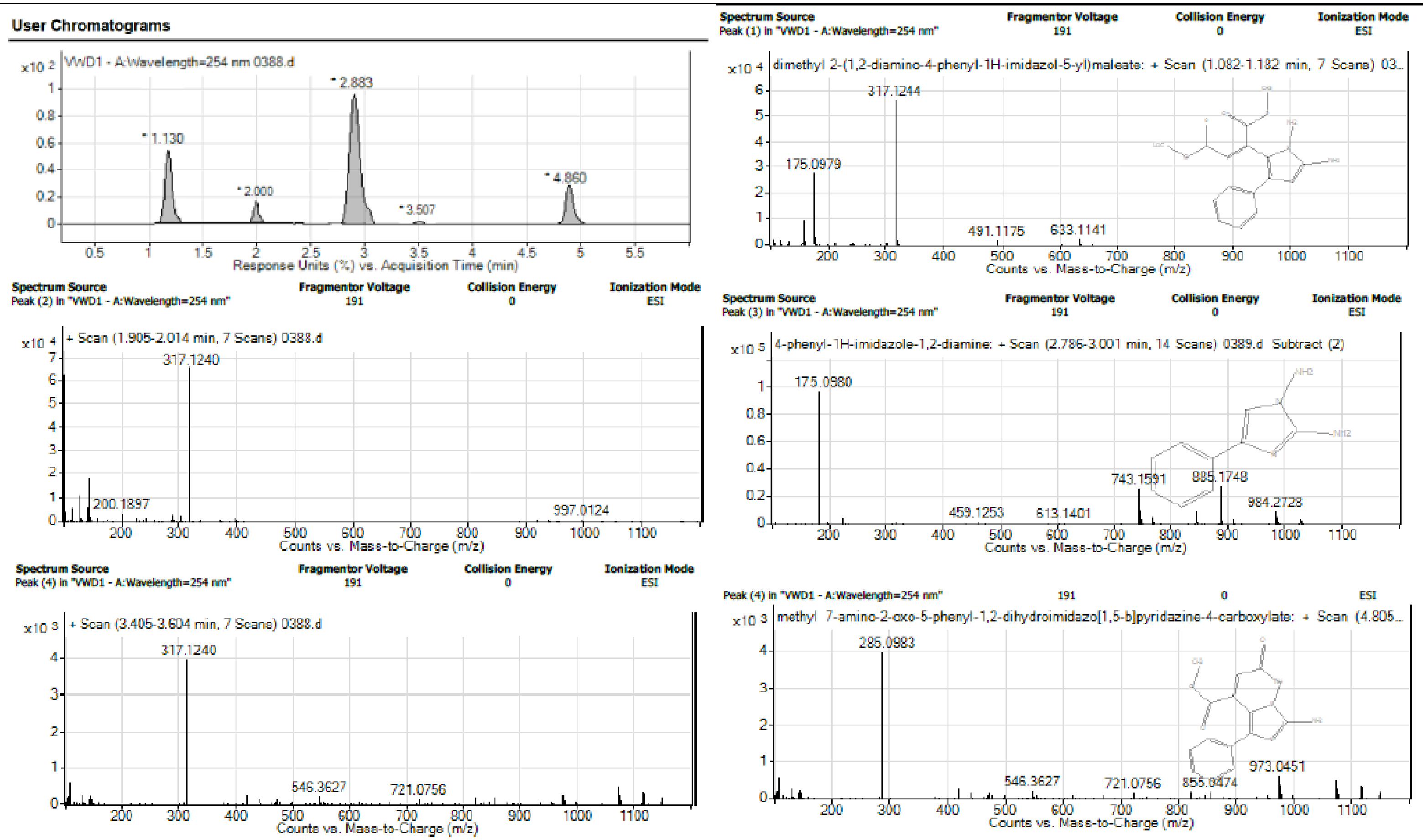

**120 minutes**	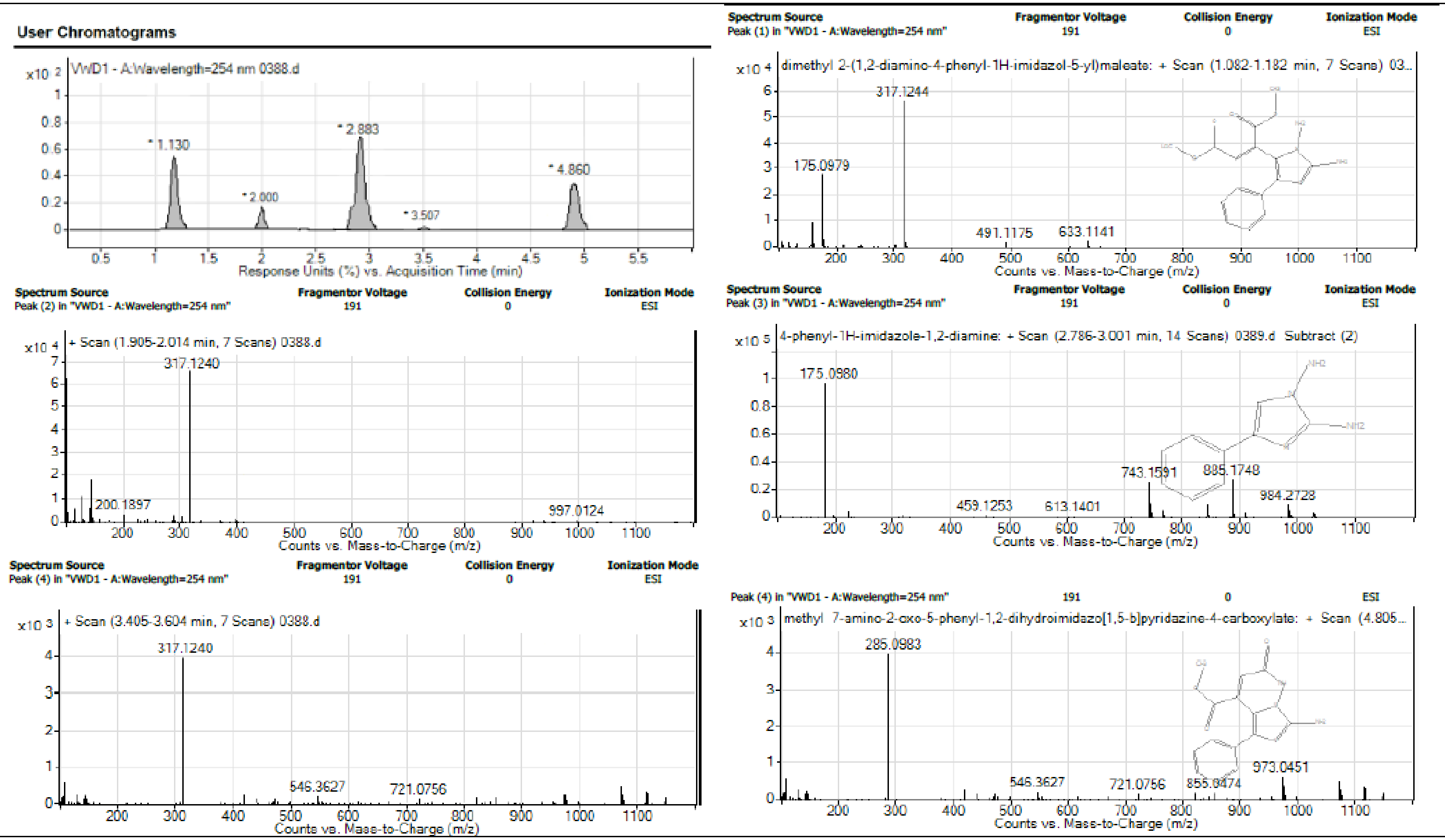

User Chromatograms
VWD1 - A:Wavelength=254 nm 0388.d
1.130
2.000
2.883
3.507
4.860
Response Units (%) vs. Acquisition Time (min)
Spectrum Source Peak (1) in "VWD1 - A:Wavelength=254 nm" Fragmentor Voltage 191 Collision Energy 0 Ionization Mode ESI
dimethyl 2-(1,2-diamino-4-phenyl-1H-imidazol-5-yl)maleate: + Scan (1.082-1.182 min, 7 Scans) 03...
317.1244
175.0979
491.1175
633.1141
Counts vs. Mass-to-Charge (m/z)
Spectrum Source Peak (2) in "VWD1 - A:Wavelength=254 nm" Fragmentor Voltage 191 Collision Energy 0 Ionization Mode ESI
+ Scan (1.905-2.014 min, 7 Scans) 0388.d
317.1240
200.1897
997.0124
Counts vs. Mass-to-Charge (m/z)
Spectrum Source Peak (3) in "VWD1 - A:Wavelength=254 nm" Fragmentor Voltage 191 Collision Energy 0 Ionization Mode ESI
4-phenyl-1H-imidazole-1,2-diamine: + Scan (2.786-3.001 min, 14 Scans) 0389.d Subtract (2)
175.0980
459.1253
613.1401
743.1591
885.1748
984.2728
Counts vs. Mass-to-Charge (m/z)
Spectrum Source Peak (4) in "VWD1 - A:Wavelength=254 nm" Fragmentor Voltage 191 Collision Energy 0 Ionization Mode ESI
+ Scan (3.405-3.604 min, 7 Scans) 0388.d
317.1240
546.3627
721.0756
Counts vs. Mass-to-Charge (m/z)
Peak (4) in "VWD1 - A:Wavelength=254 nm" 191 0 ESI
methyl 7-amino-2-oxo-5-phenyl-1,2-dihydroimidazo[1,5-b]pyridazine-4-carboxylate: + Scan (4.805...
285.0983
546.3627
721.0756
855.0474
973.0451
Counts vs. Mass-to-Charge (m/z)

Table S6. Conducting the reaction in methanol.

<table>
<tr>
<td>60 minutes</td>
<td>
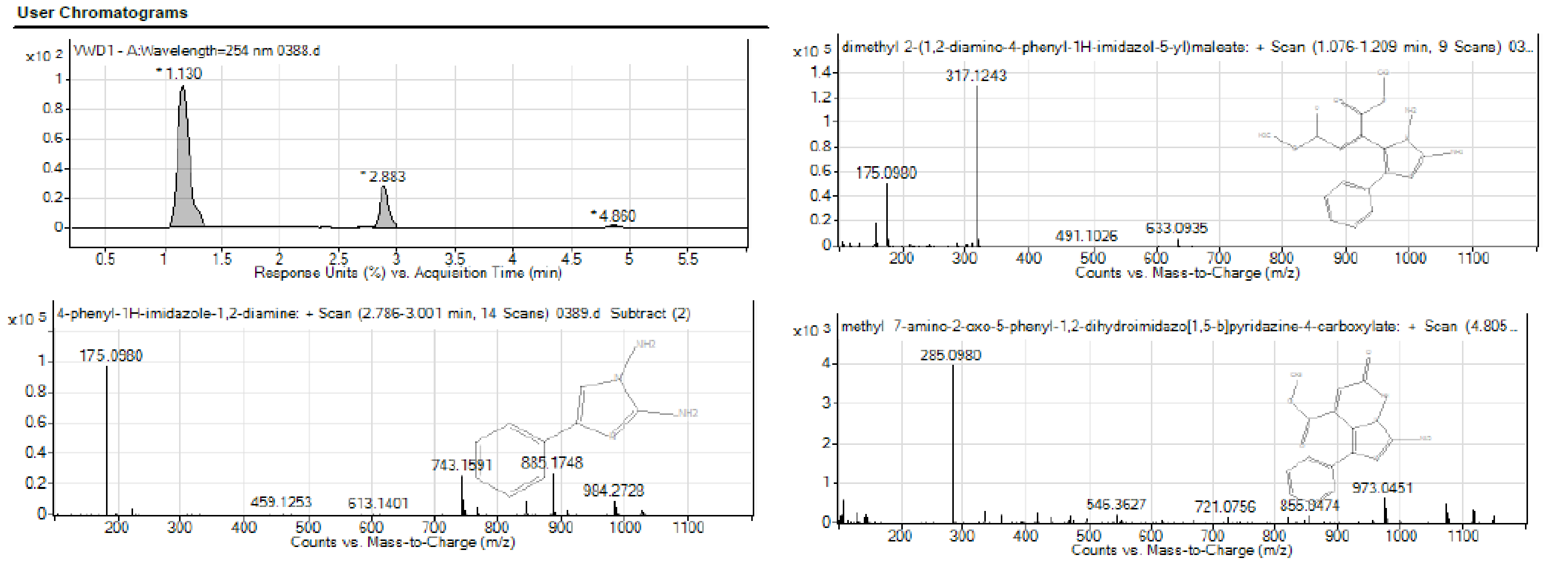

</td>
</tr>
<tr>
<td>120 minutes</td>
<td>
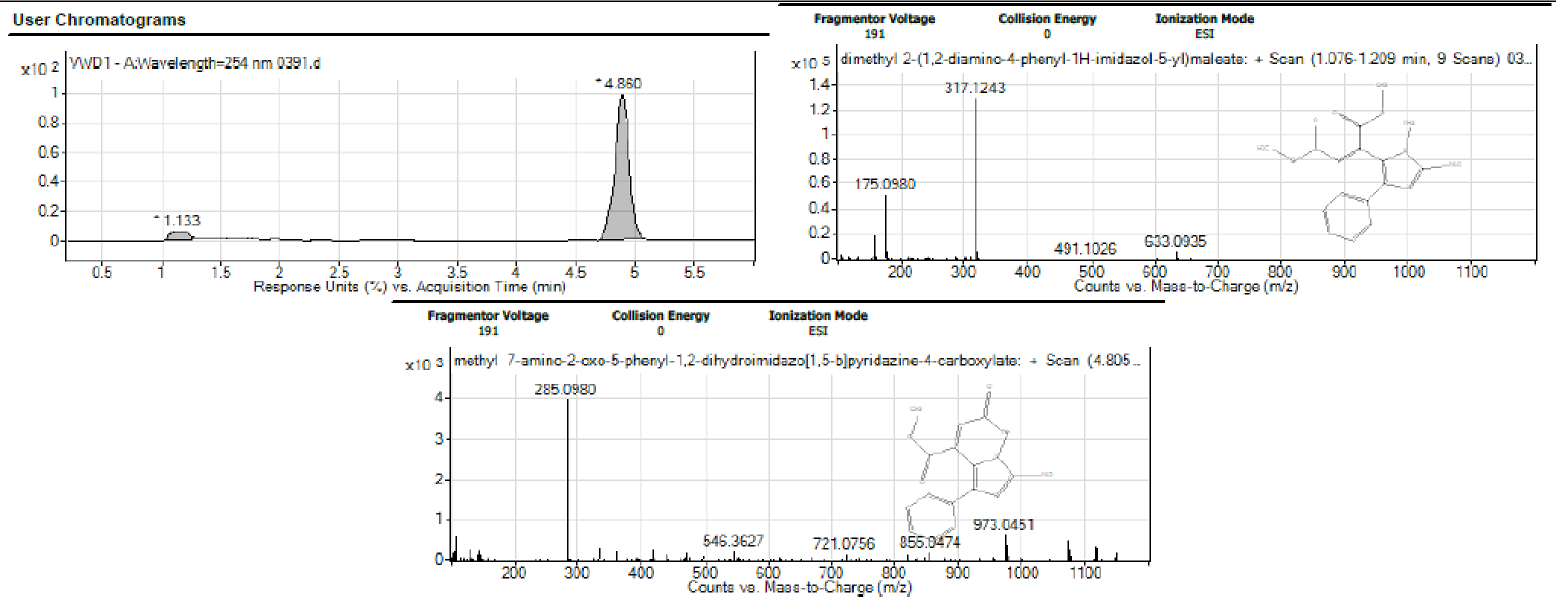

</td>
</tr>
</table>

Table S7. Conducting the reaction in ethanol.

<table>
<tr><td>60 minutes</td><td>
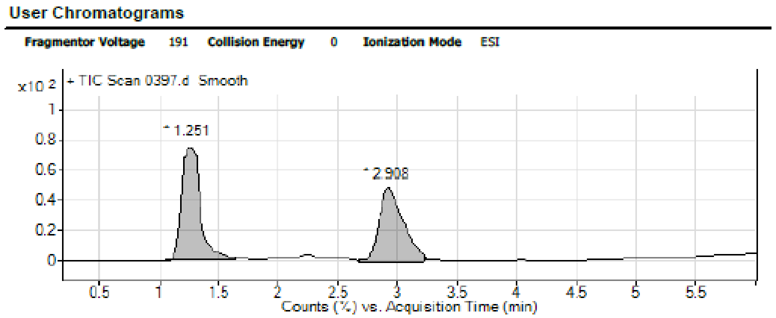

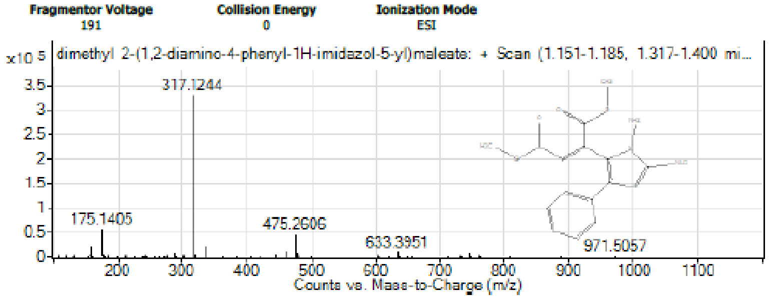

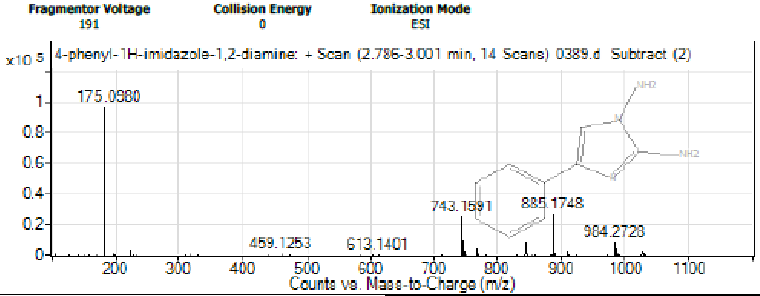

</td></tr>
<tr><td>120 minutes</td><td>
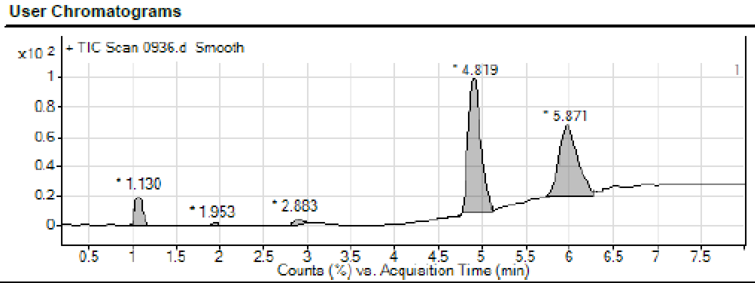

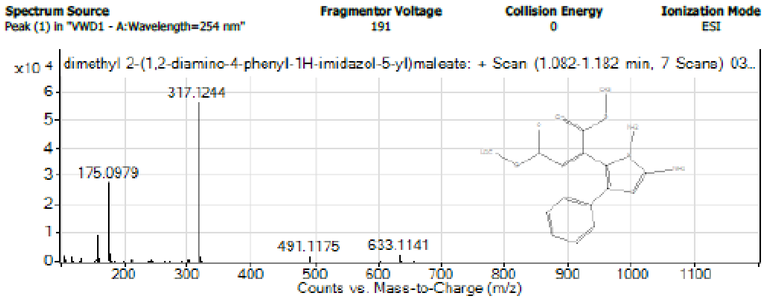

</td></tr>
</table>

	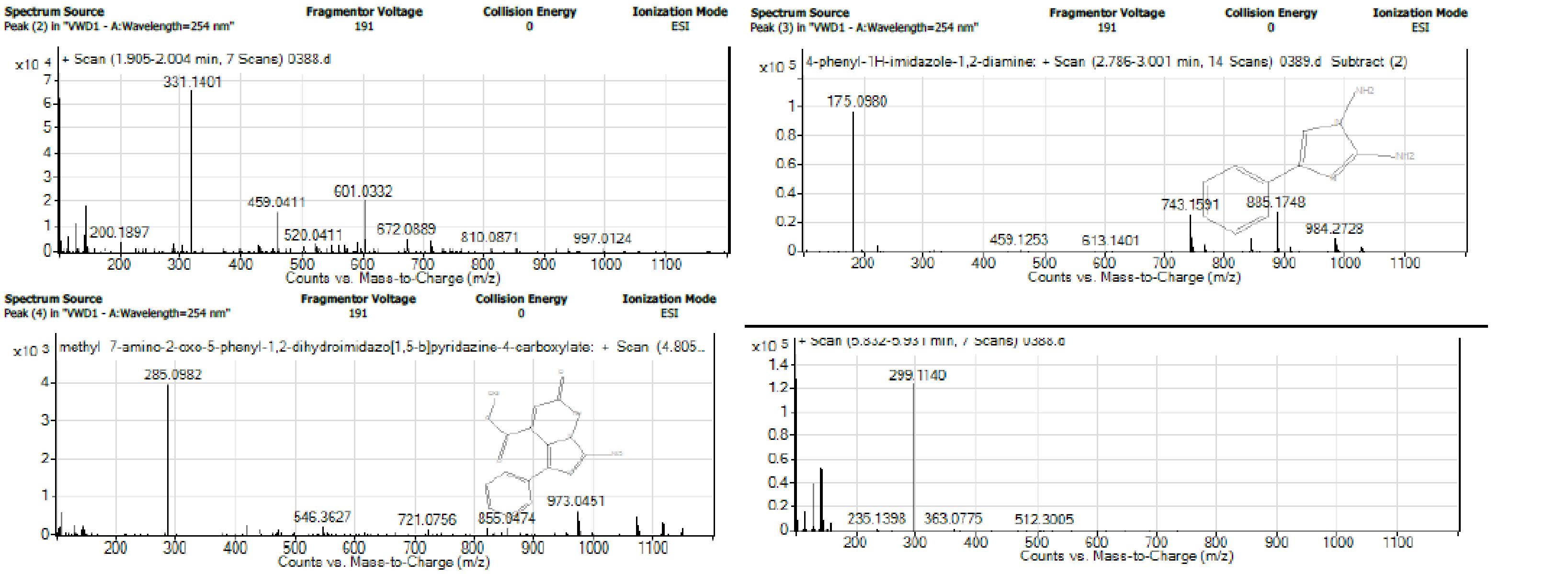

Table S8. Reaction in methanol with the addition of acetic acid as a catalyst.

**60 minutes**	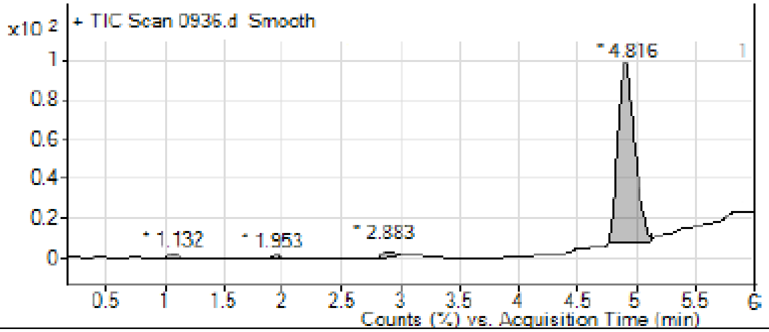 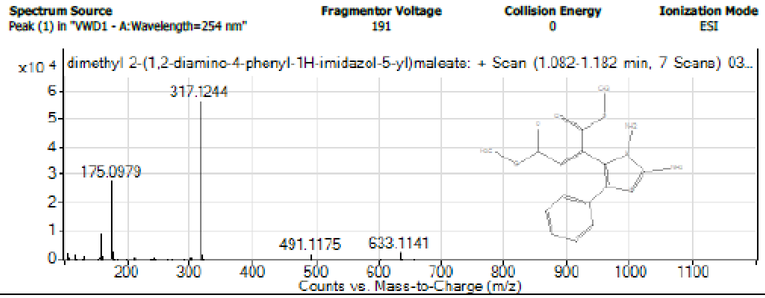

	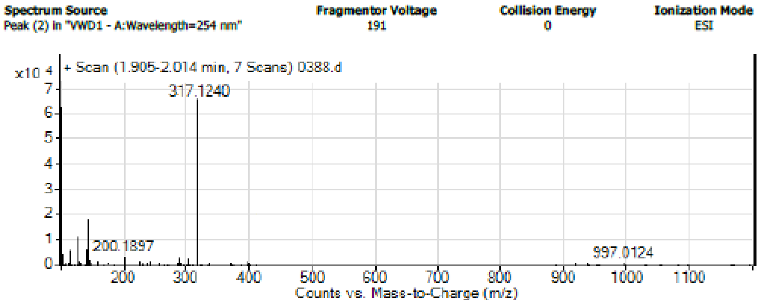 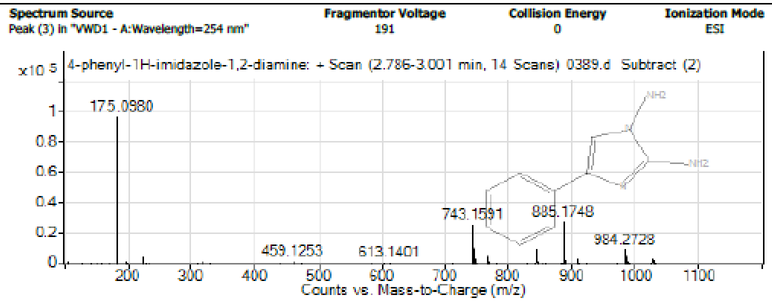 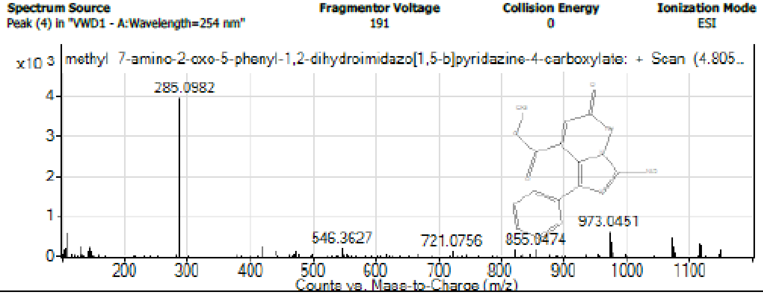

Table S9. Conducting the reaction in a 1:1 mixture of methanol and acetic acid.

**60 minutes**	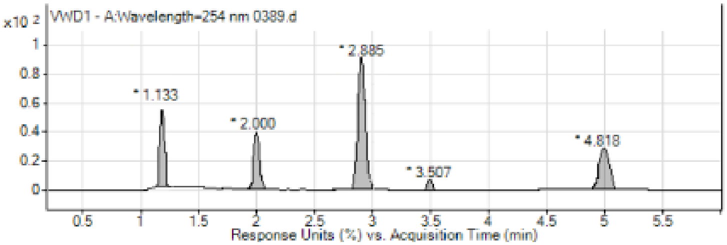 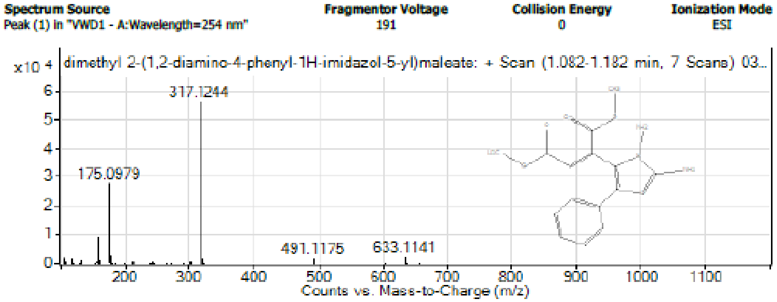

<table>
<tr>
<td></td>
<td>
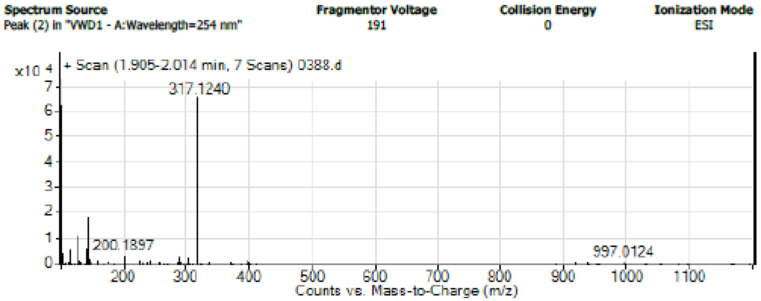

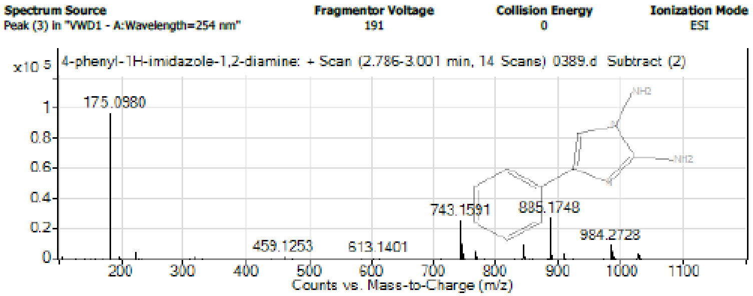

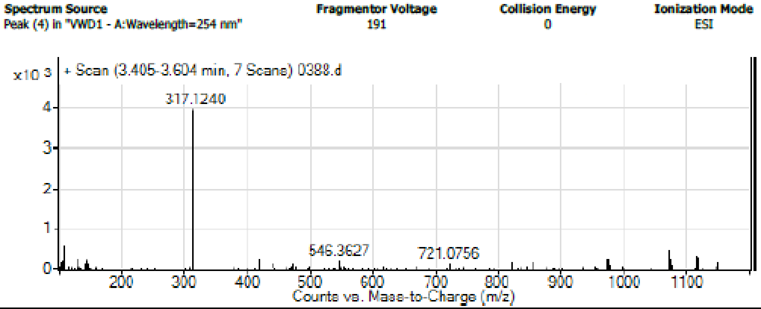

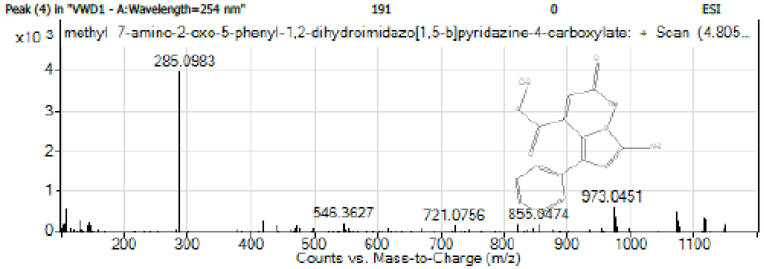

</td>
</tr>
<tr>
<td>120 minutes</td>
<td>
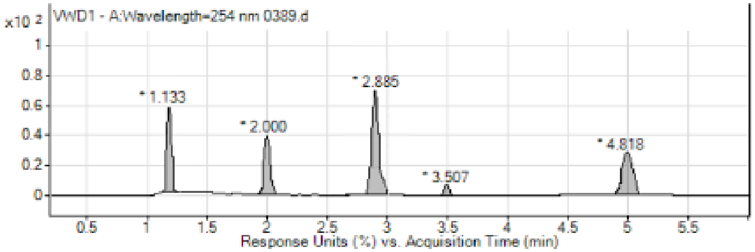

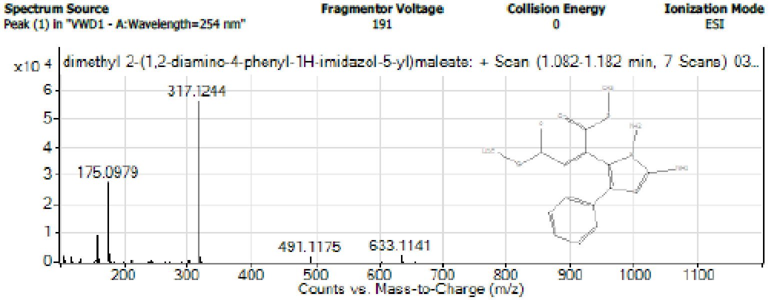

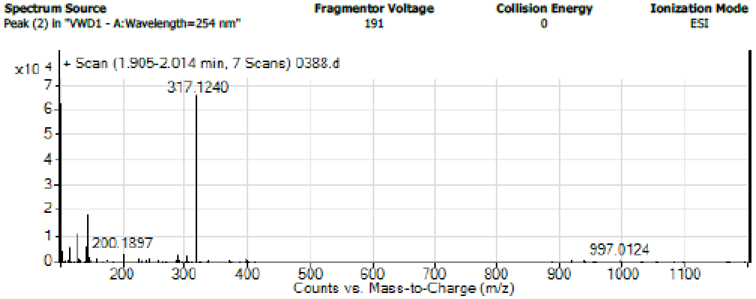

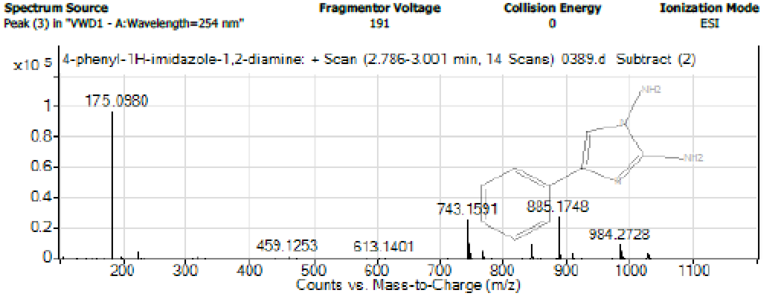

</td>
</tr>
</table>

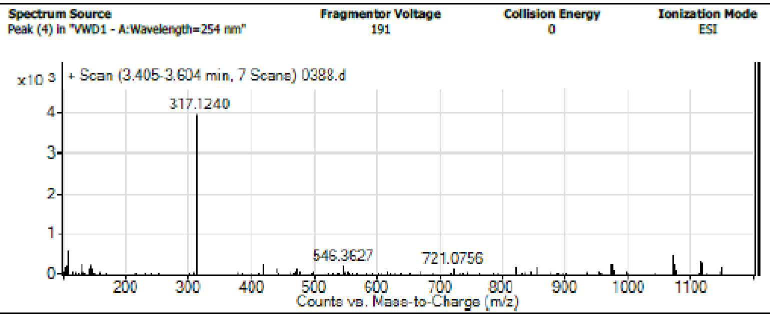

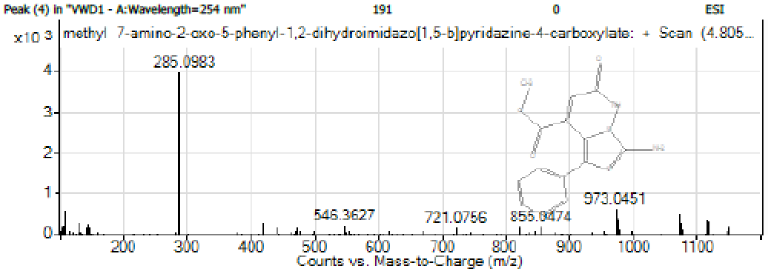

Table S10. Conducting the reaction in acetic acid.

60 minutes

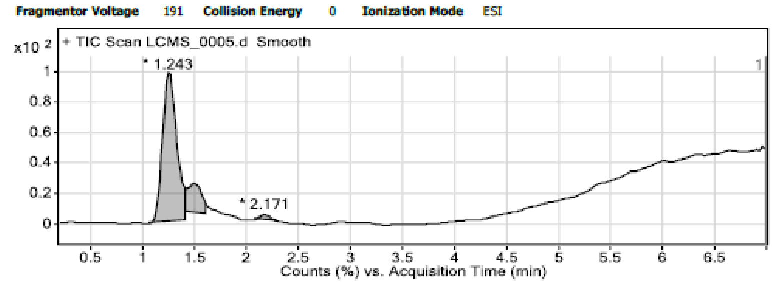

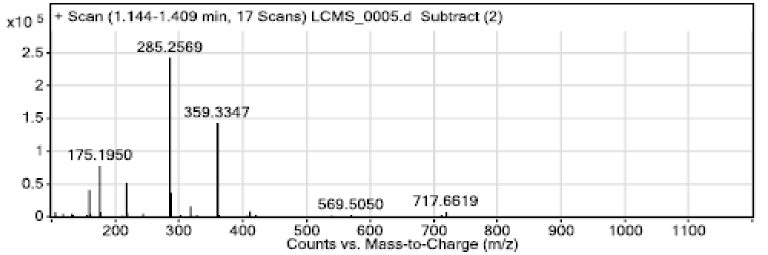

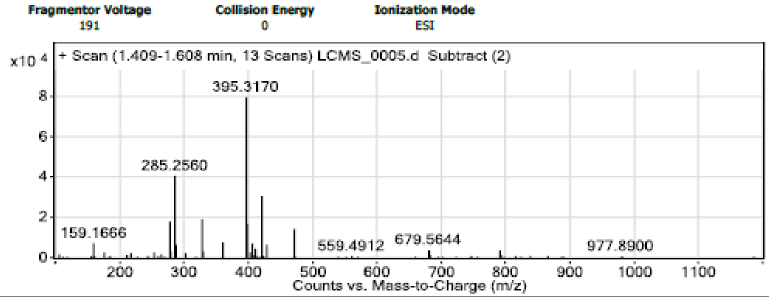

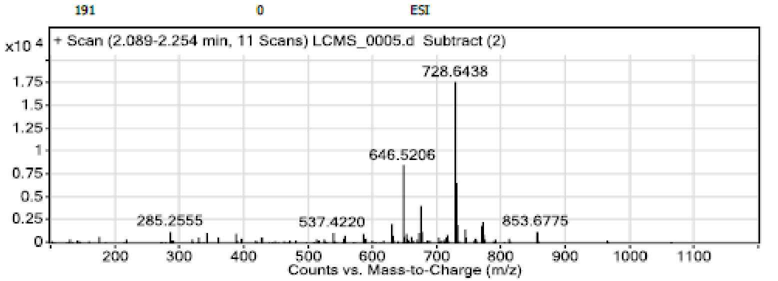

Spectral data of the obtained compounds

Table S11.

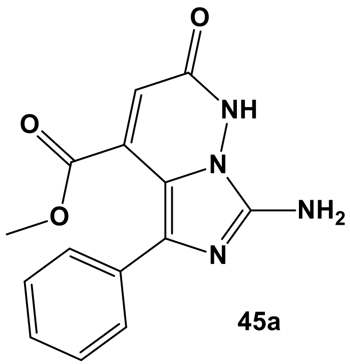 **45a** 7-amino-1,2-dihydro-2-oxo-5-phenylimidazo[1,5-b]pyridazine-4-carboxylate	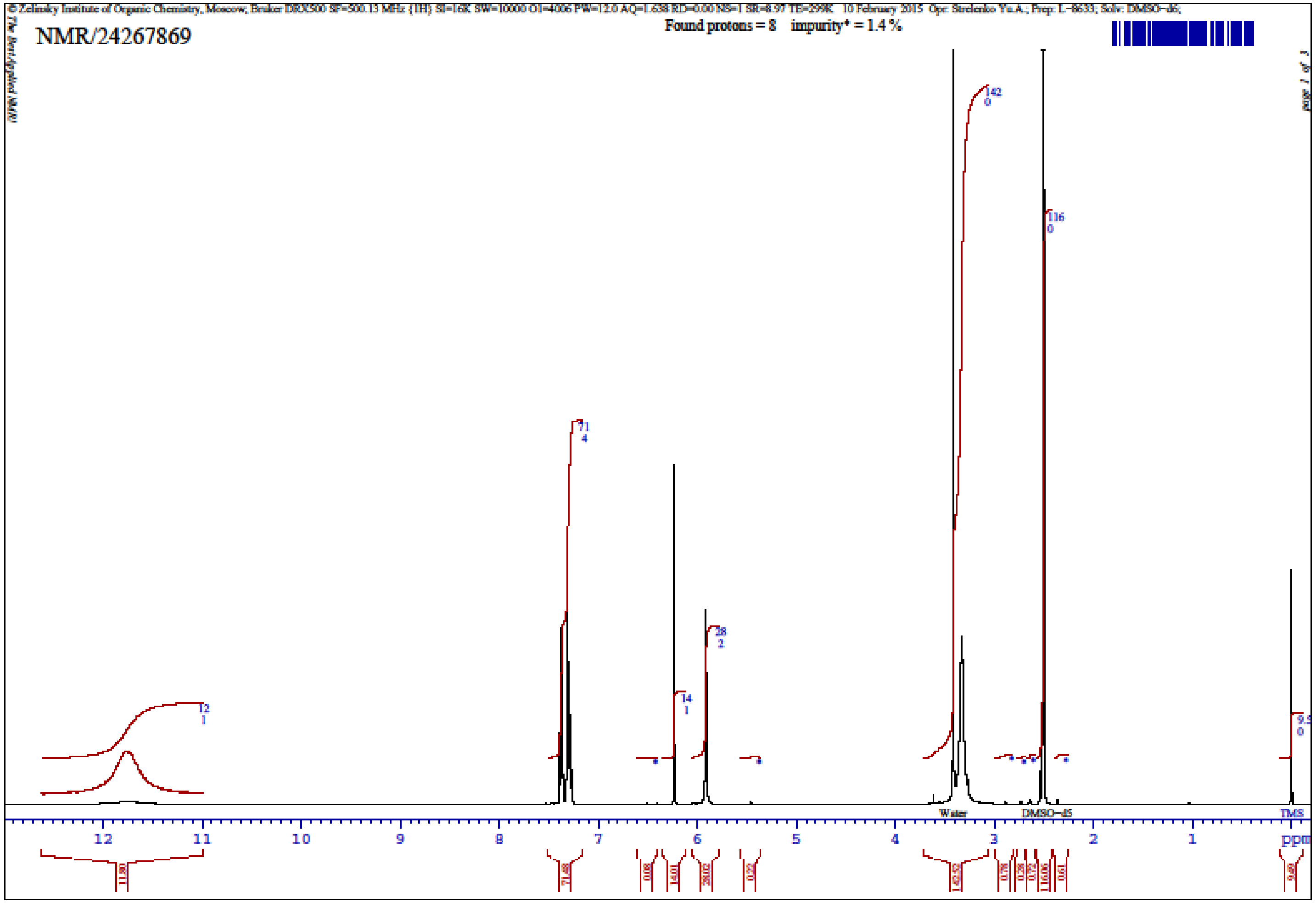

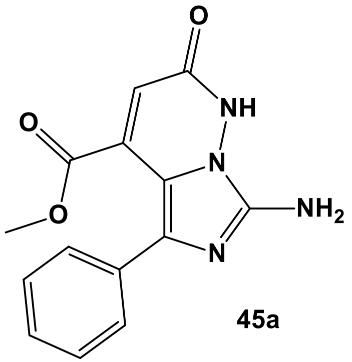

45a

7-amino-1,2-dihydro-2-oxo-5-phenylimidazo[1,5-b]pyridazine-4-carboxylate

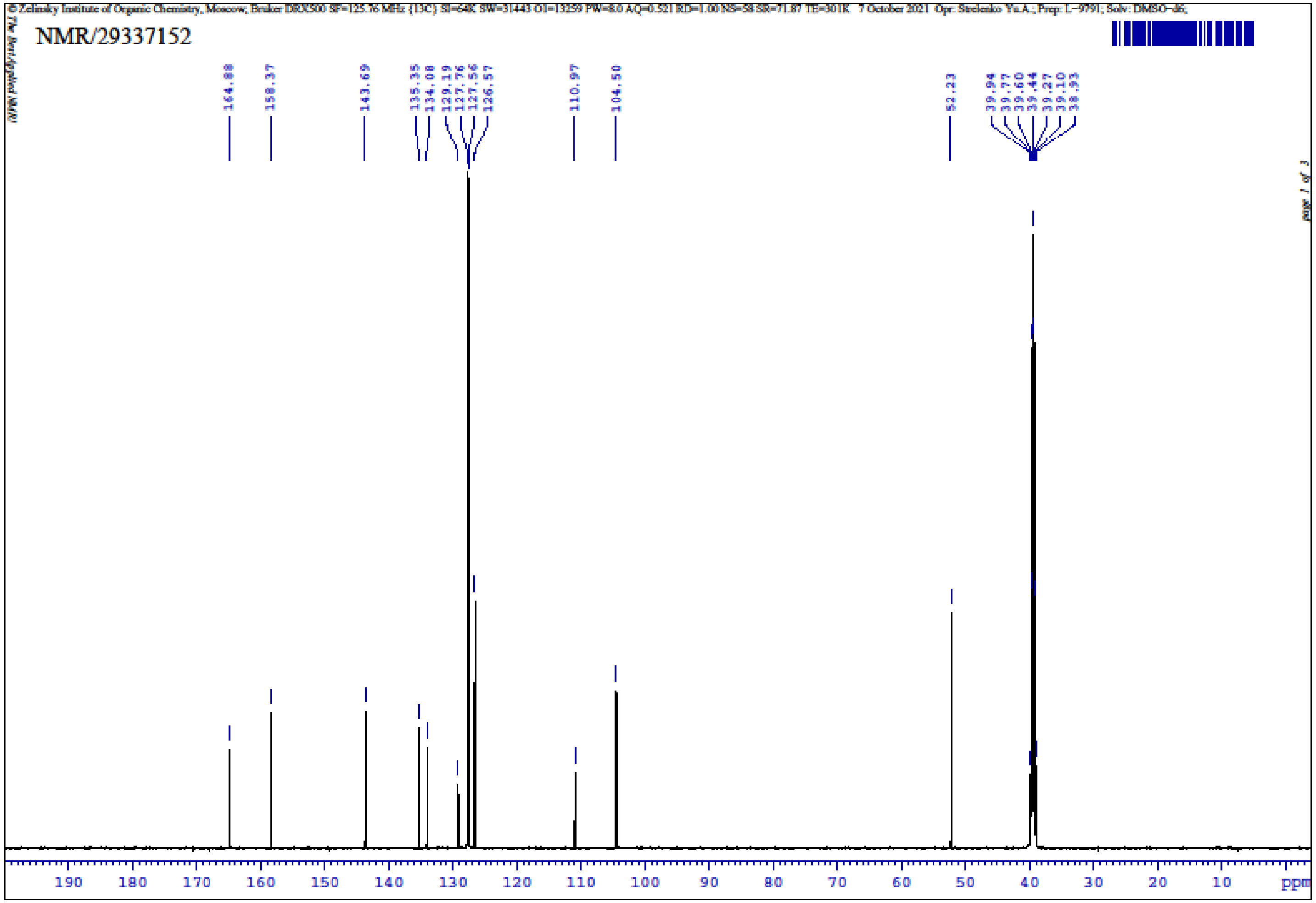

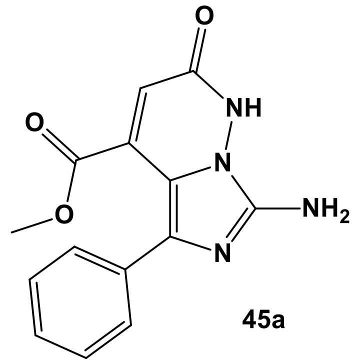

45a

7-amino-1,2-dihydro-2-oxo-5-phenylimidazo[1,5-b]pyridazine-4-carboxylate

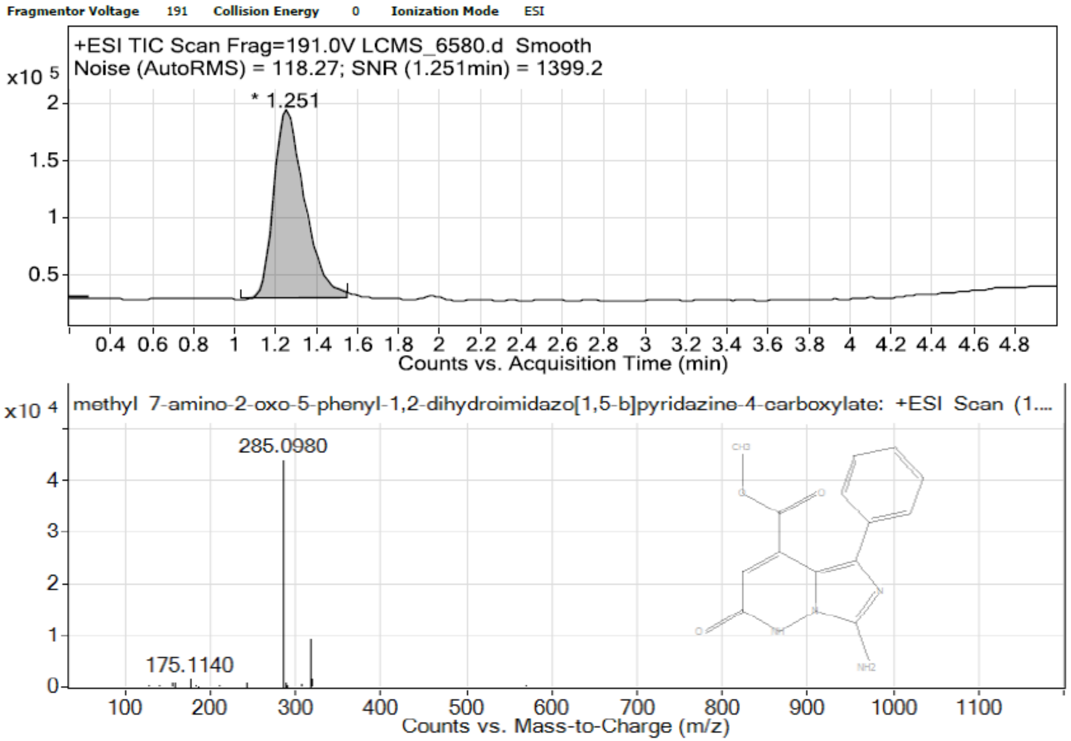

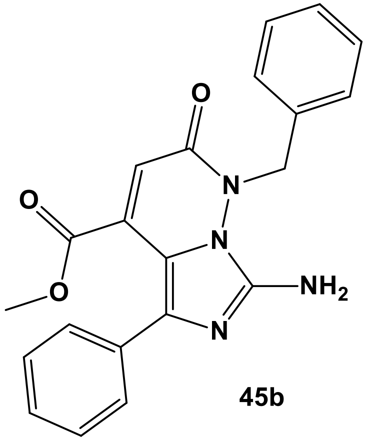

45b

7-amino-1-benzyl-1,2-dihydro-2-oxo-5-phenylimidazo[1,5-b]pyridazine-4-carboxylate

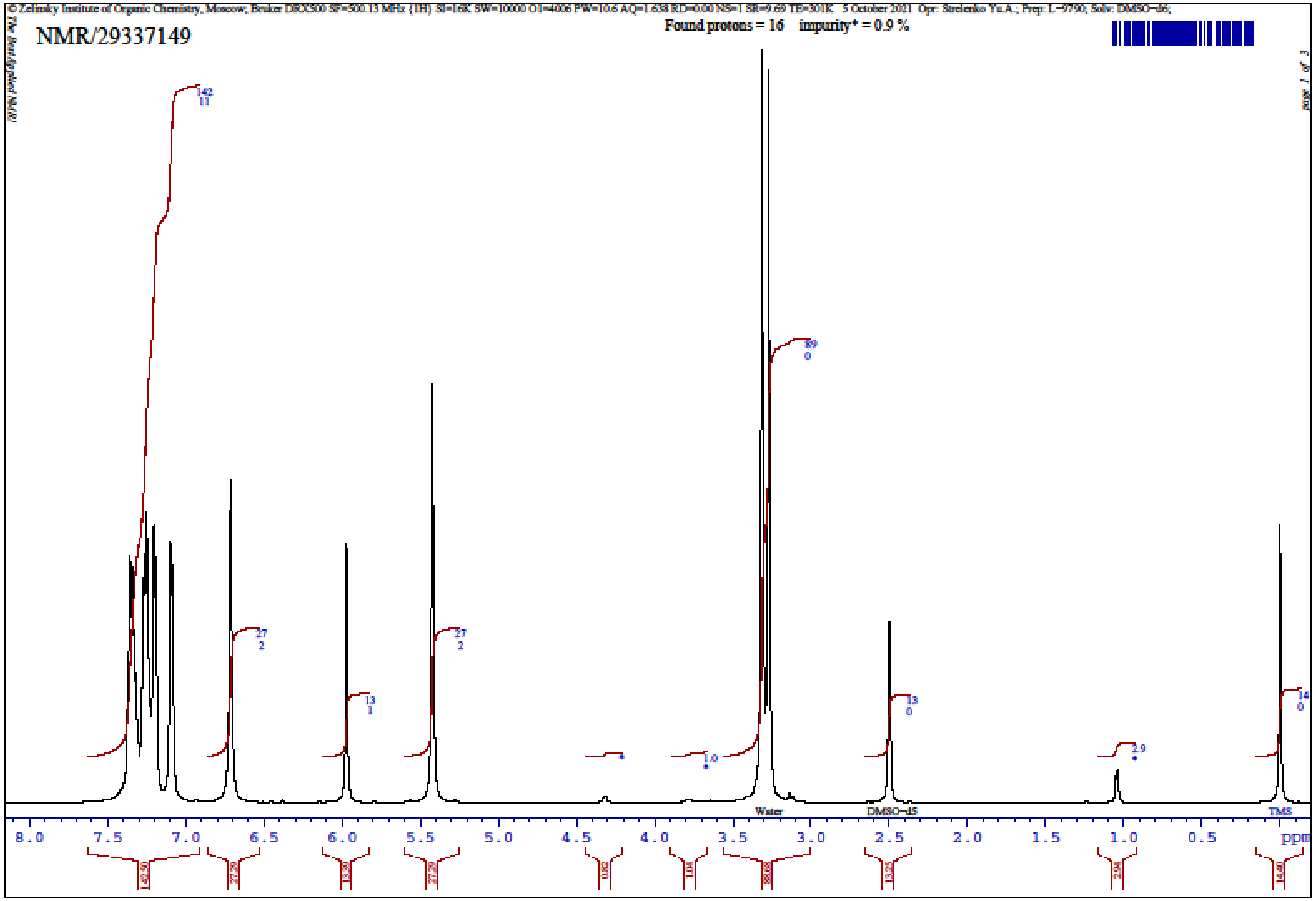

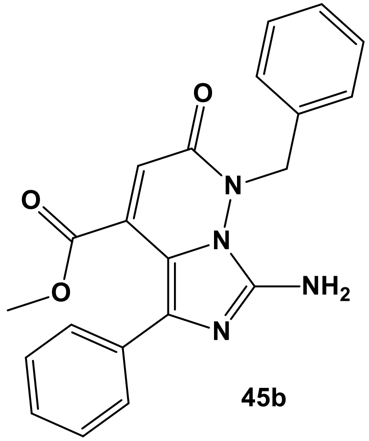

45b

7-amino-1-benzyl-1,2-dihydro-2-oxo-5-phenylimidazo[1,5-b]pyridazine-4-carboxylate

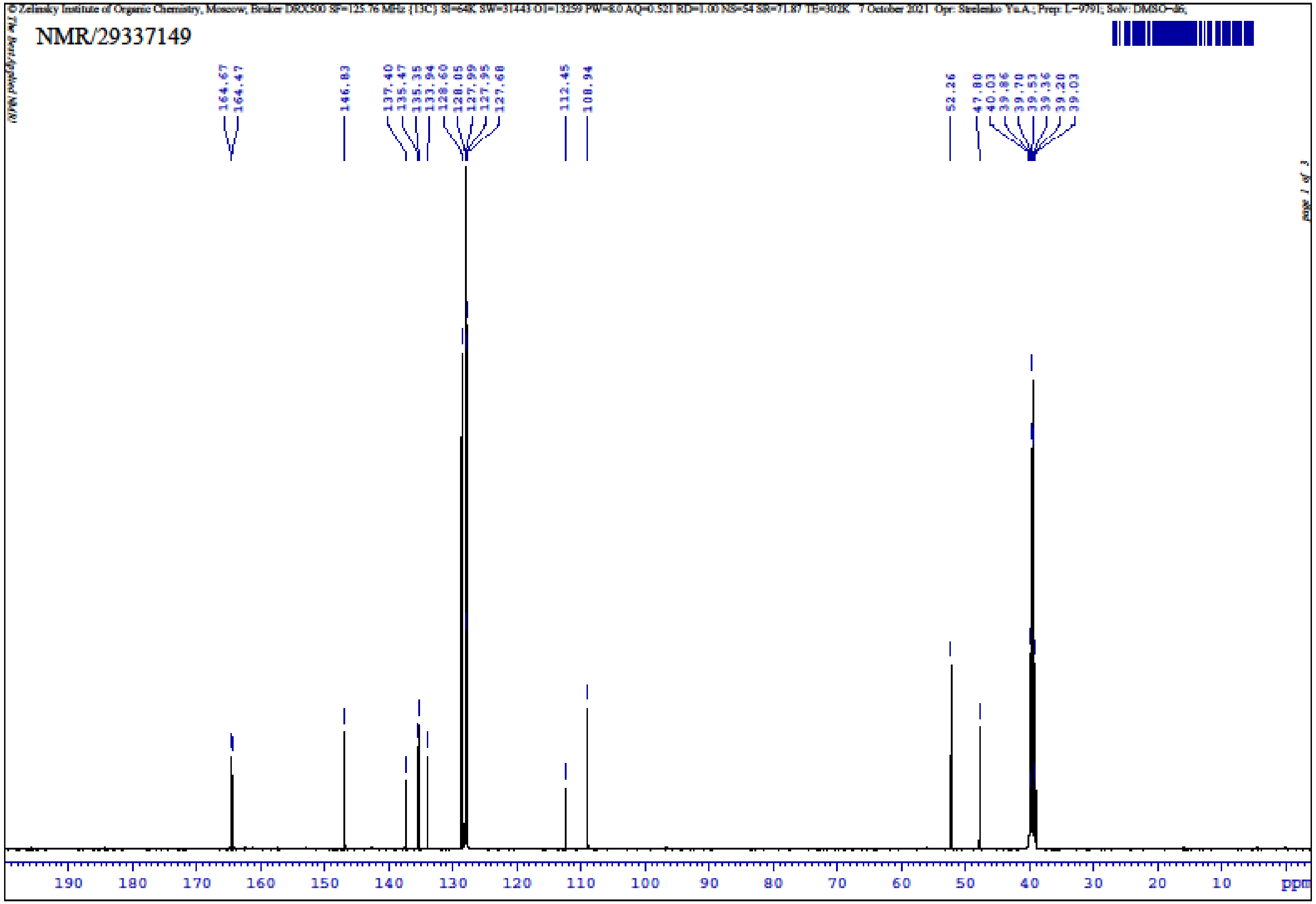

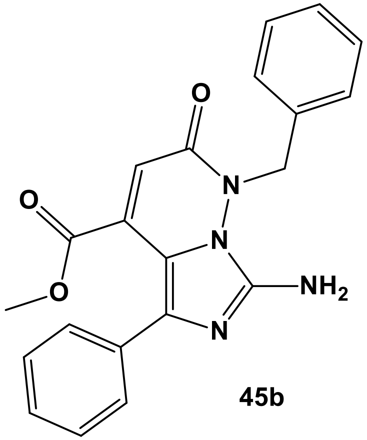

45b

7-amino-1-benzyl-1,2-dihydro-2-oxo-5-phenylimidazo[1,5-b]pyridazine-4-carboxylate

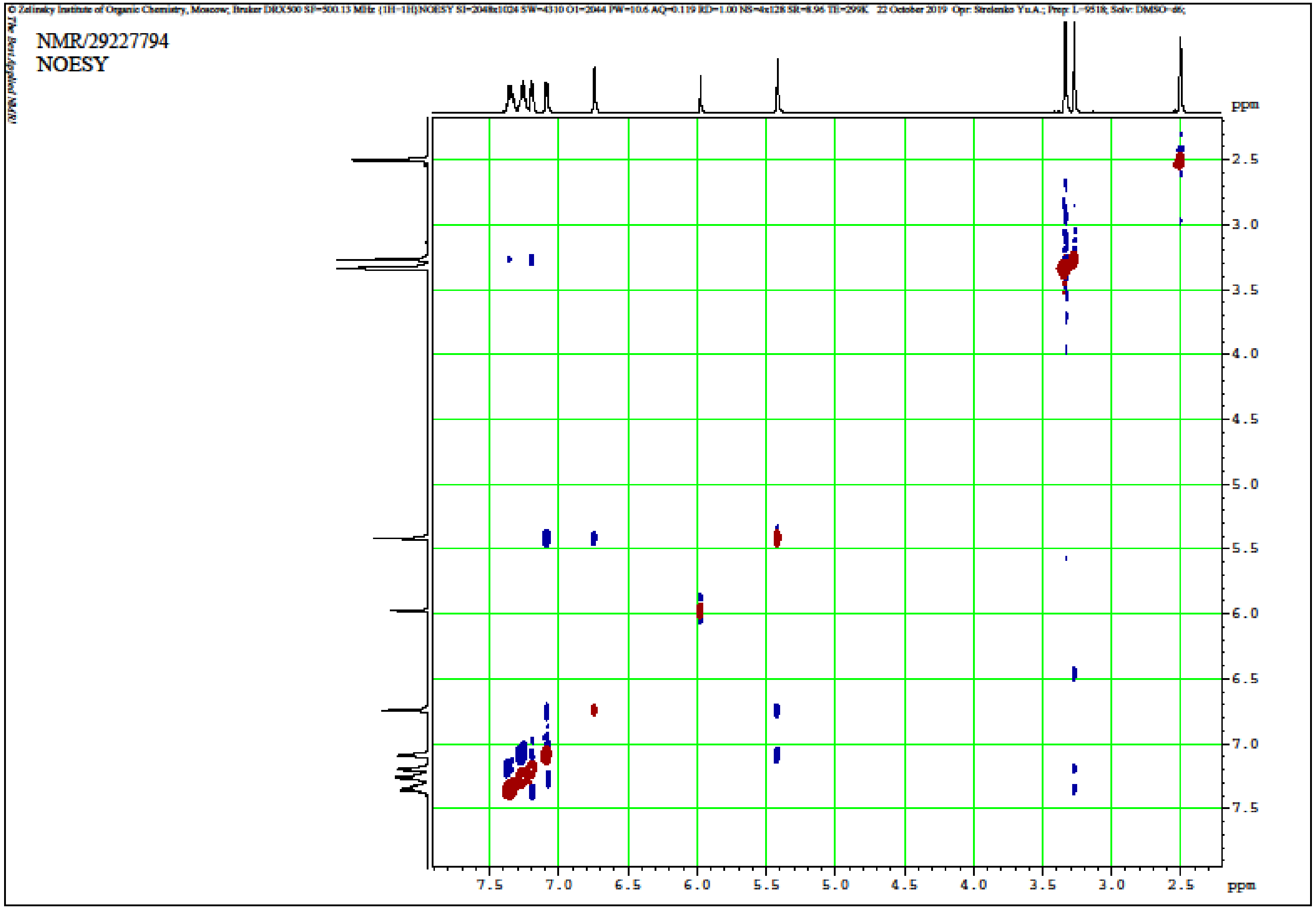

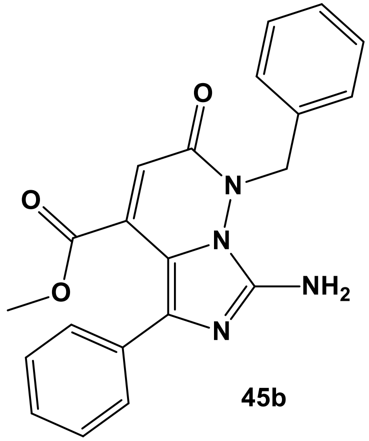

45b

7-amino-1-benzyl-1,2-dihydro-2-oxo-5-phenylimidazo[1,5-b]pyridazine-4-carboxylate

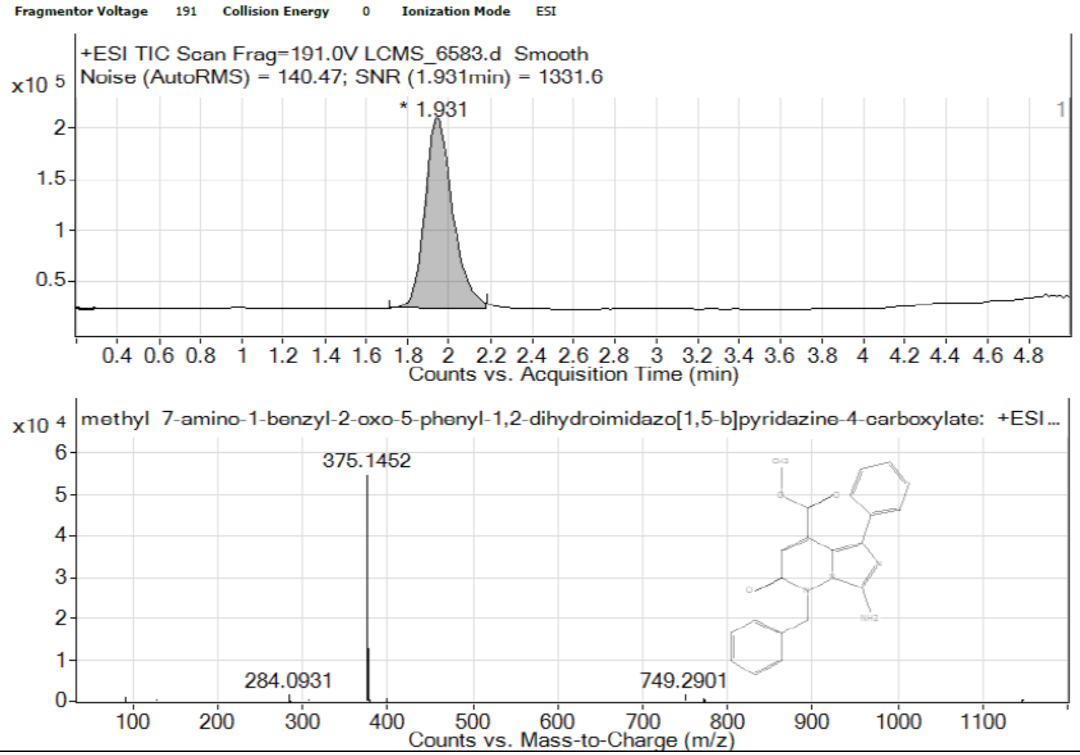

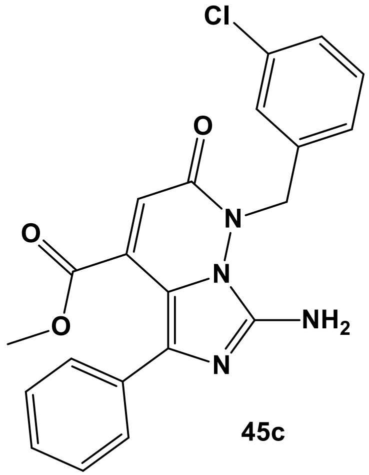

45c

7-amino-1-(3-chlorobenzyl)-1,2-dihydro-2-oxo-5-phenylimidazo[1,5-b]pyridazine-4-carboxylate

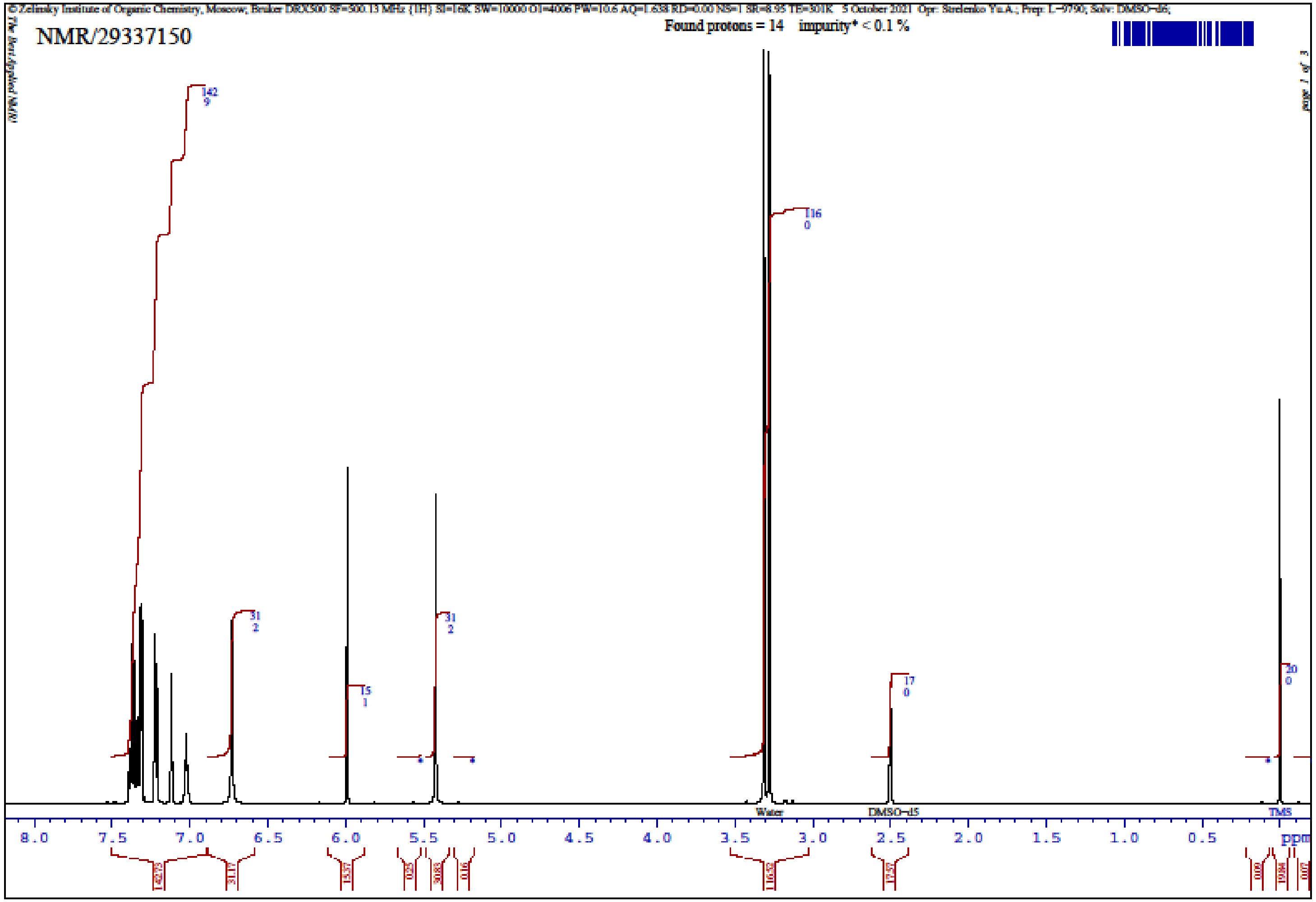

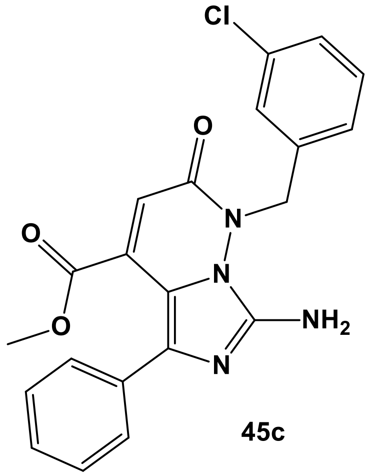

45c

7-amino-1-(3-chlorobenzyl)-1,2-dihydro-2-oxo-5-phenylimidazo[1,5-b]pyridazine-4-carboxylate

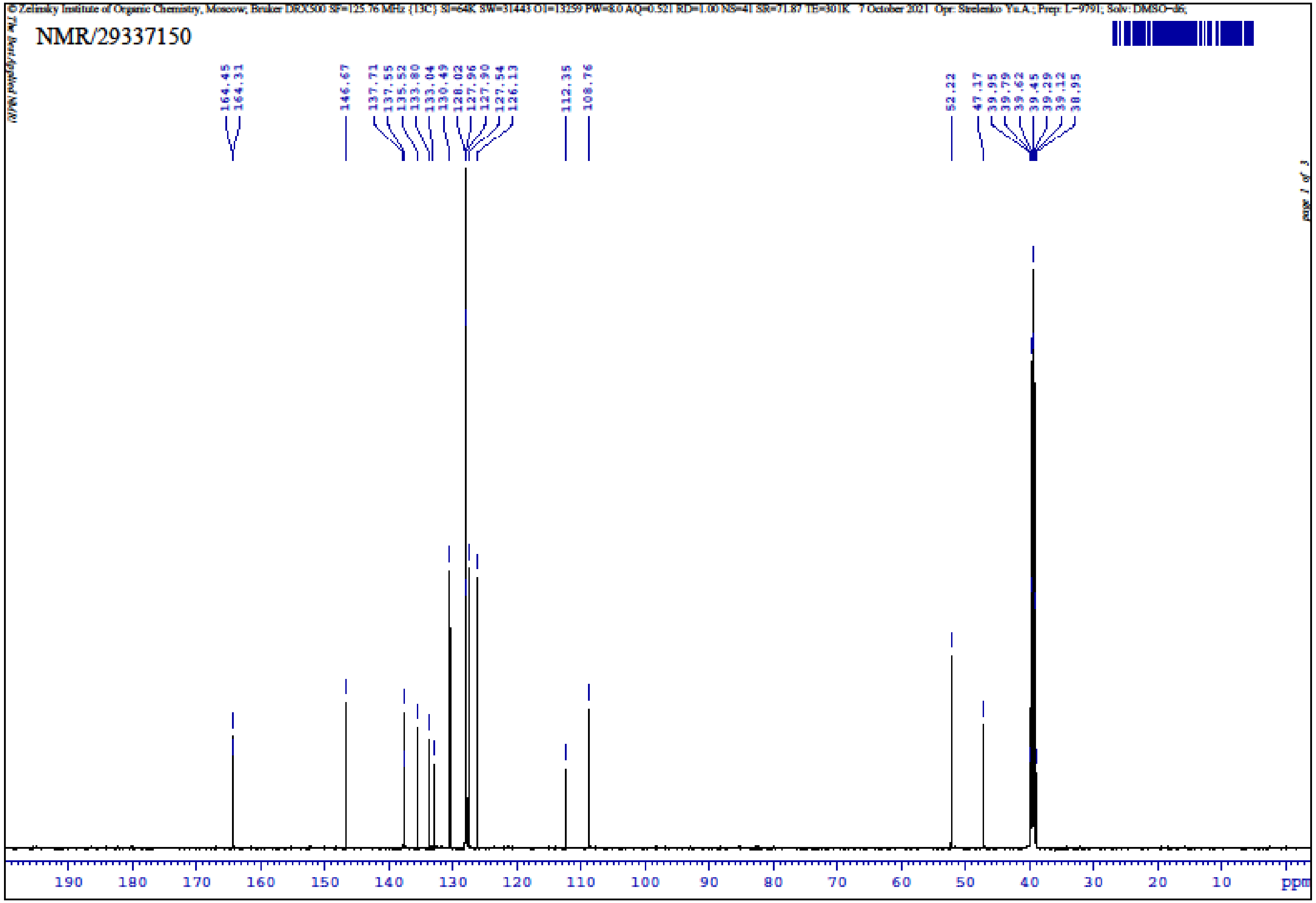

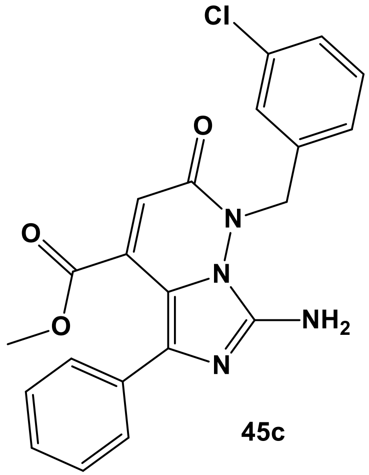

45c

7-amino-1-(3-chlorobenzyl)-1,2-dihydro-2-oxo-5-phenylimidazo[1,5-b]pyridazine-4-carboxylate

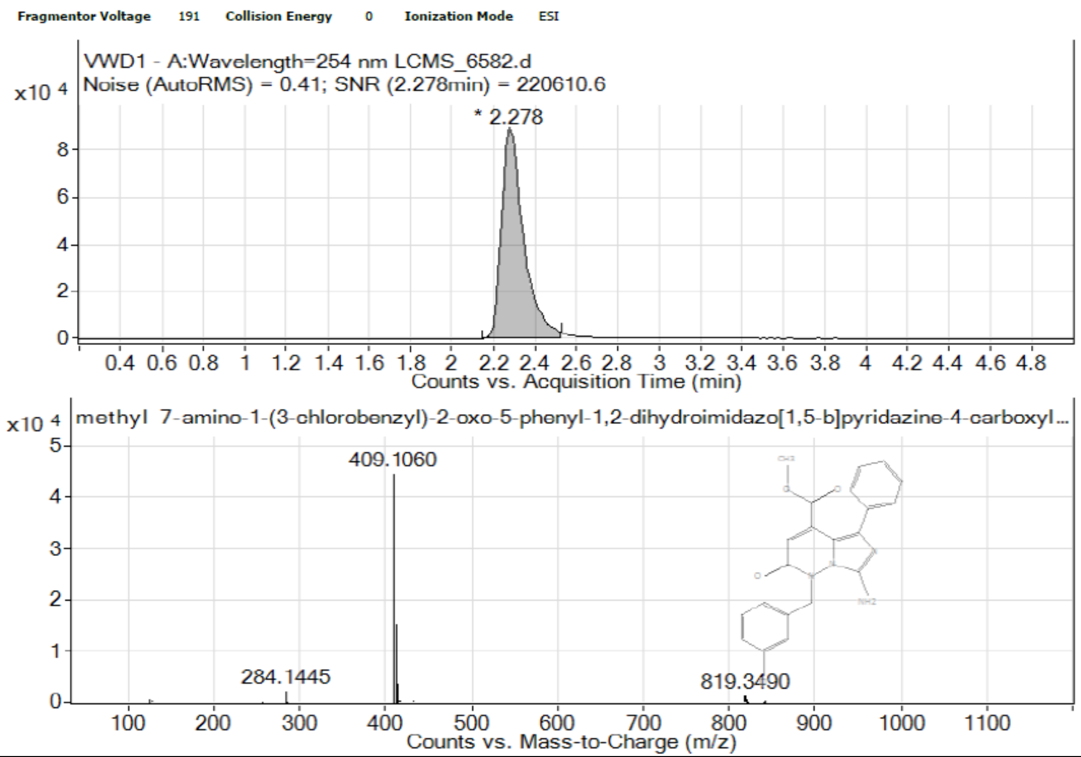

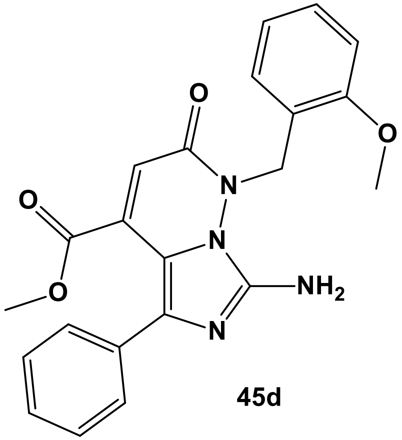

45d

7-amino-1-(2-methoxybenzyl)-1,2-dihydro-2-oxo-5-phenylimidazo[1,5-b]pyridazine-4-carboxylate

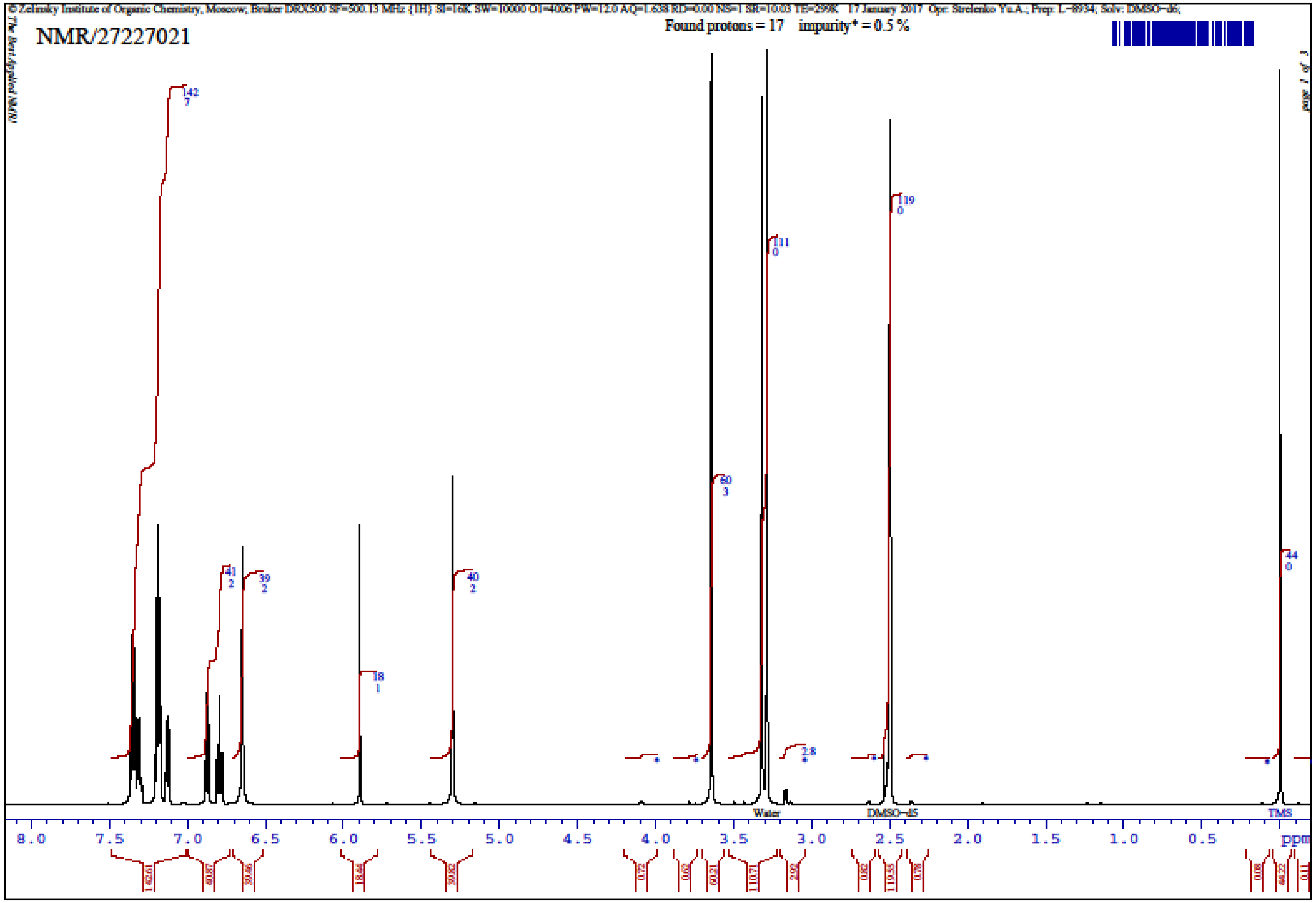

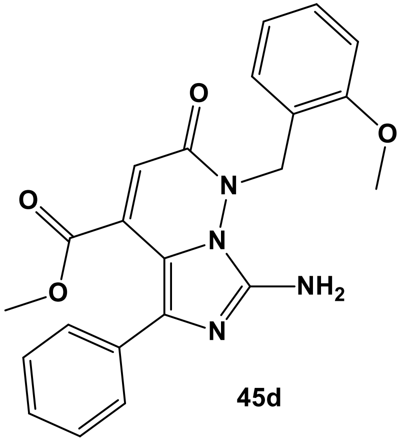

45d

7-amino-1-(2-methoxybenzyl)-1,2-dihydro-2-oxo-5-phenylimidazo[1,5-b]pyridazine-4-carboxylate

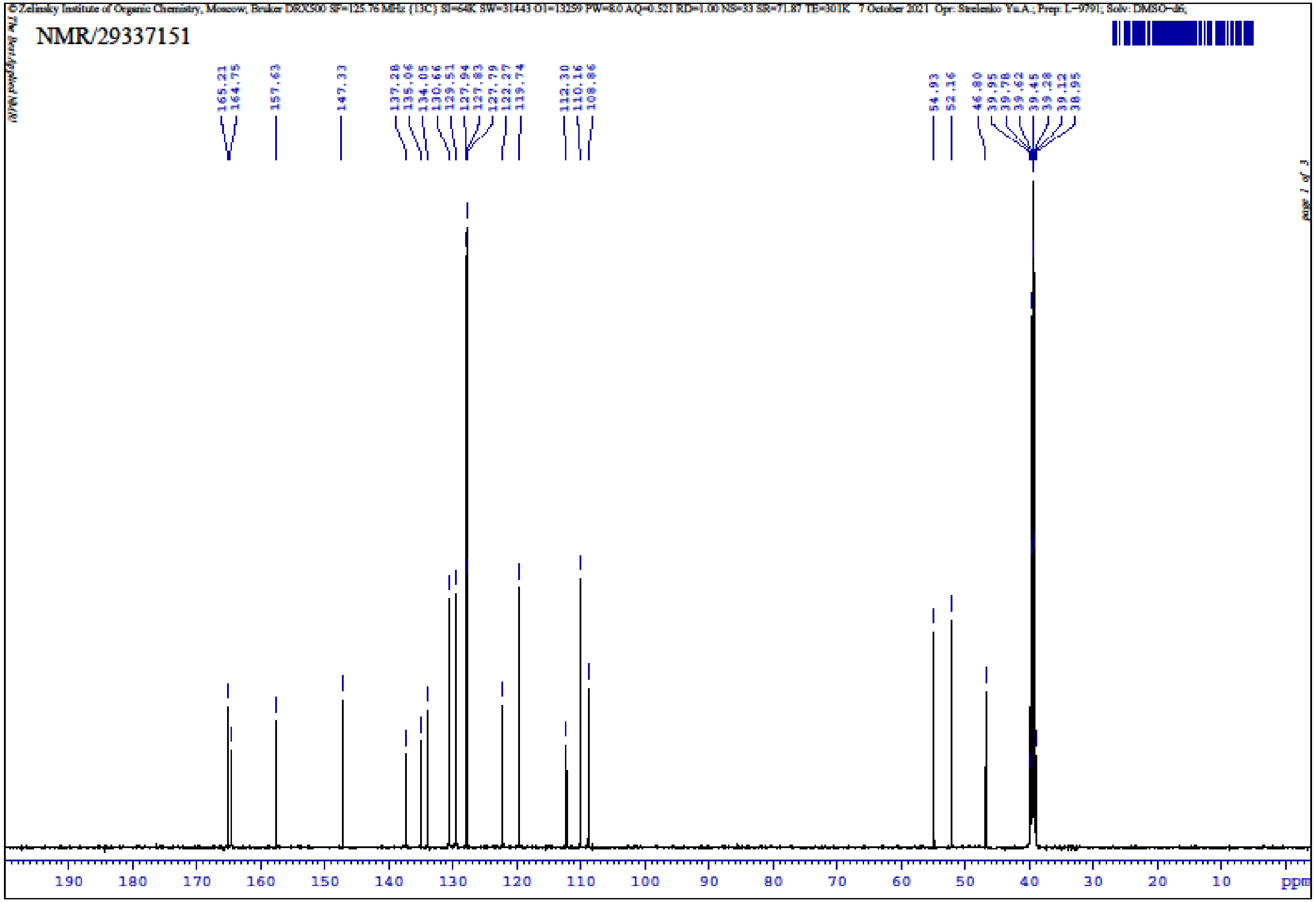

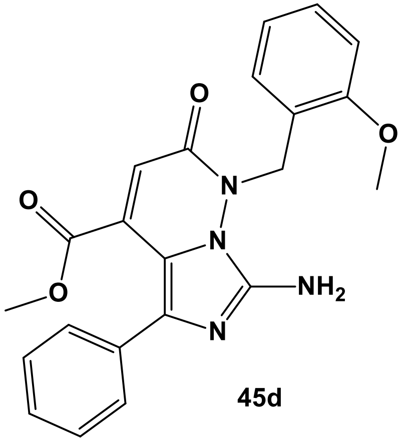

45d

7-amino-1-(2-methoxybenzyl)-1,2-dihydro-2-oxo-5-phenylimidazo[1,5-b]pyridazine-4-carboxylate

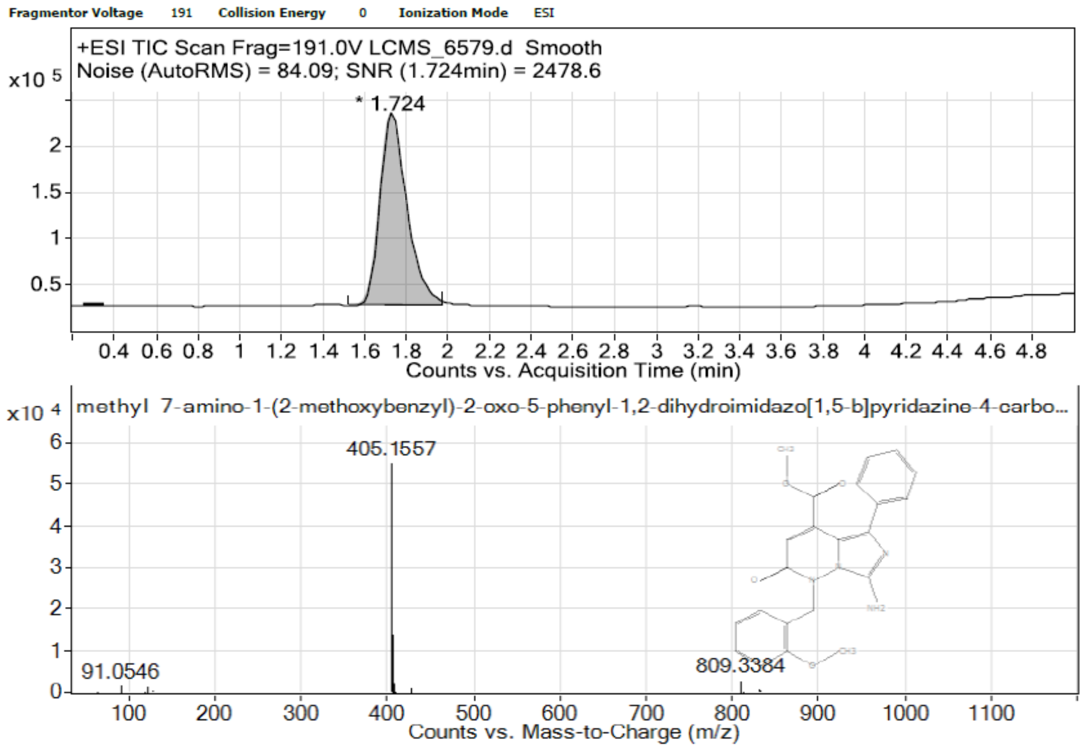

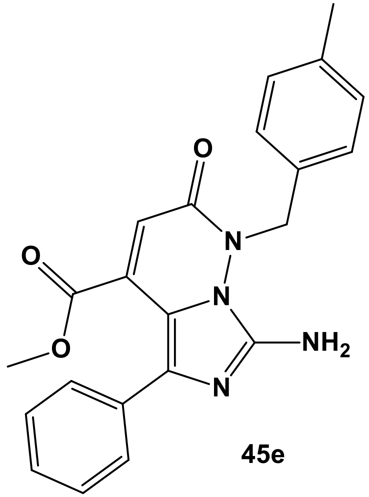

45e

7-amino-1-(4-methylbenzyl)-1,2-dihydro-2-oxo-5-phenylimidazo[1,5-b]pyridazine-4-carboxylate

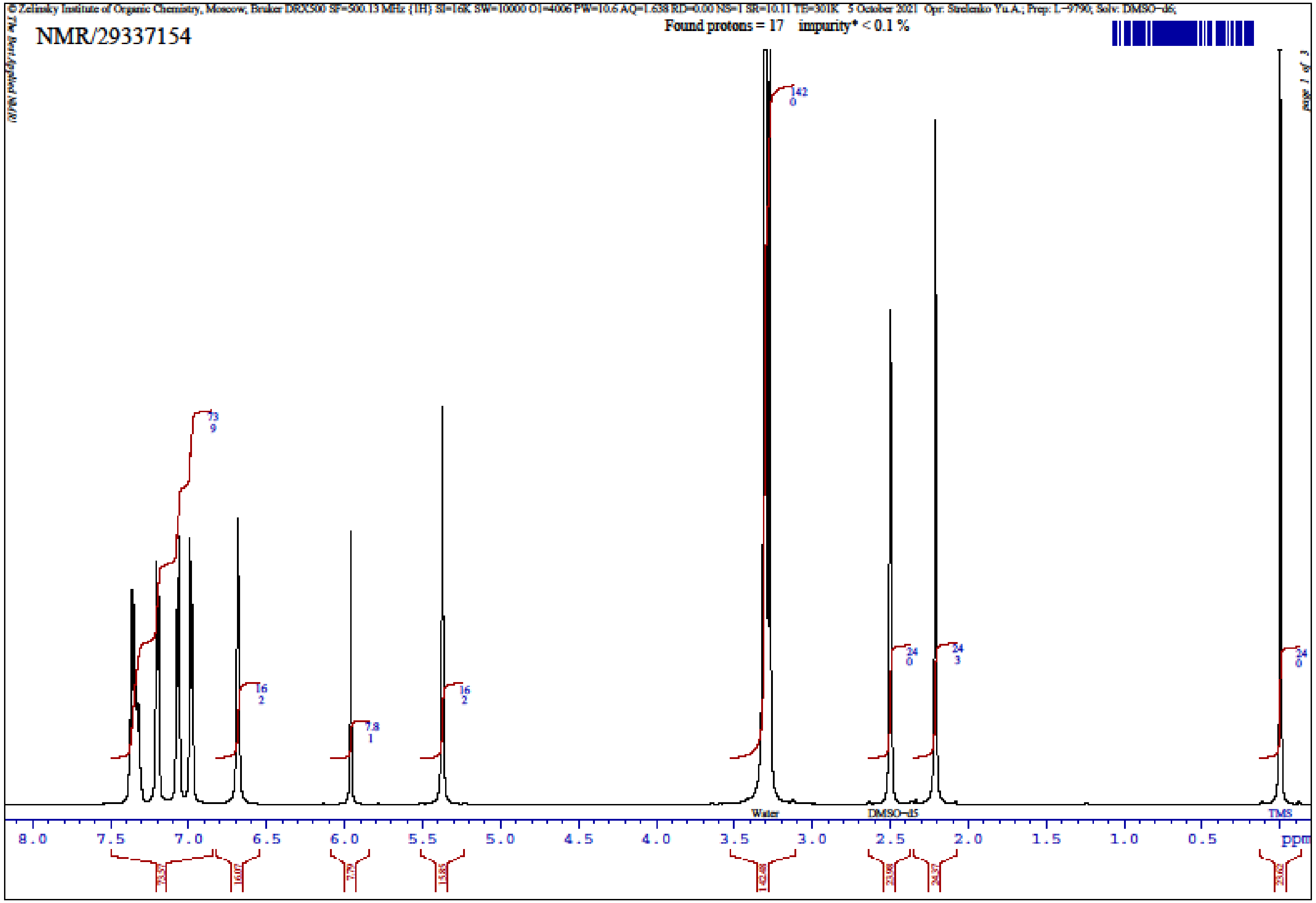

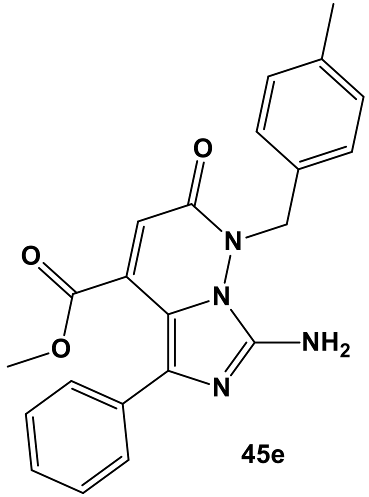

45e

7-amino-1-(4-methylbenzyl)-1,2-dihydro-2-oxo-5-phenylimidazo[1,5-b]pyridazine-4-carboxylate

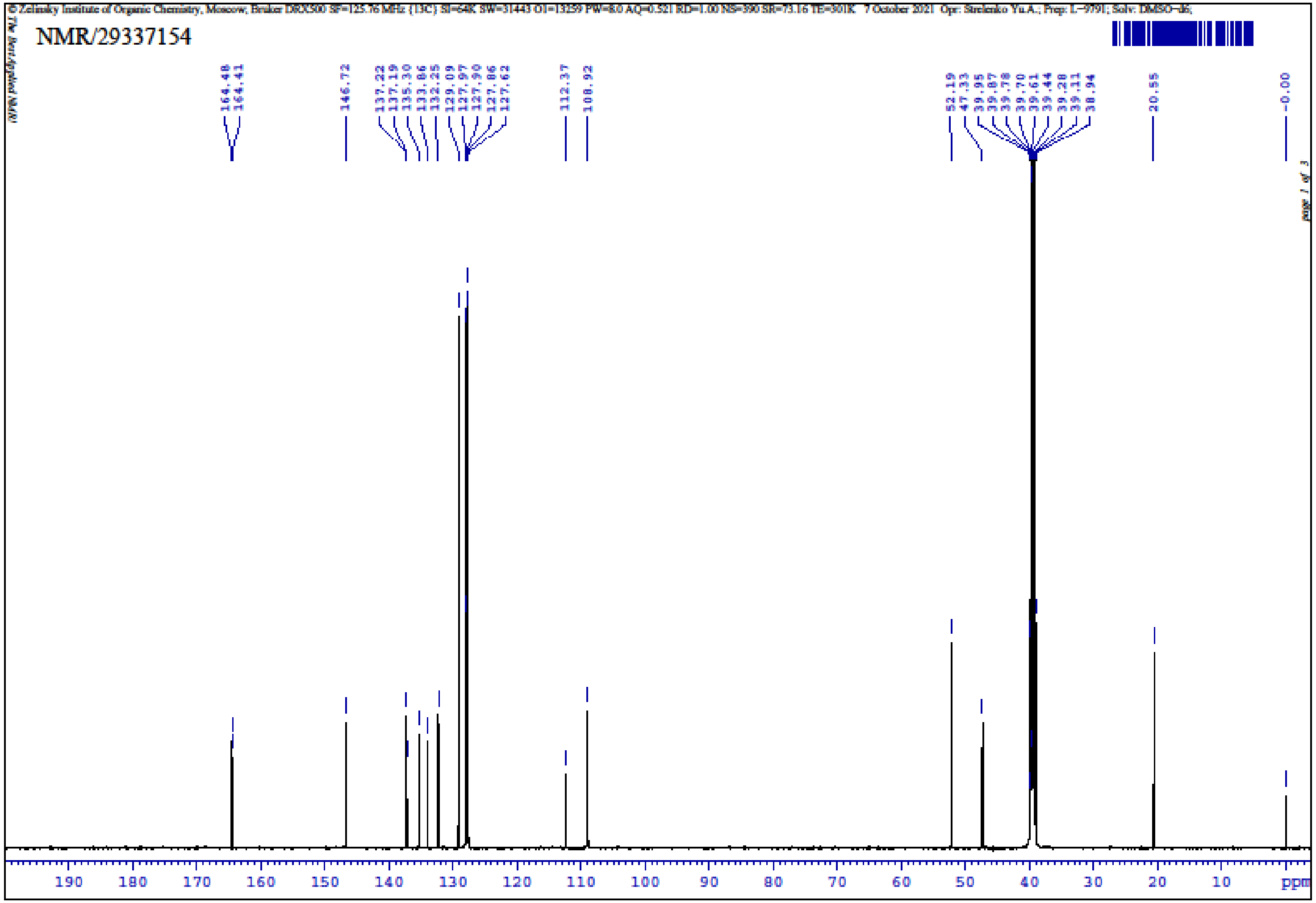

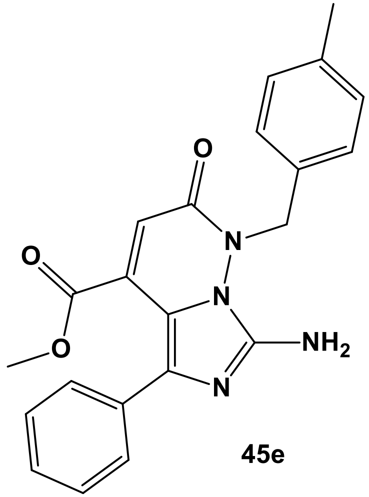 **45e** 7-amino-1-(4-methylbenzyl)-1,2-dihydro-2-oxo-5-phenylimidazo[1,5-b]pyridazine-4-carboxylate	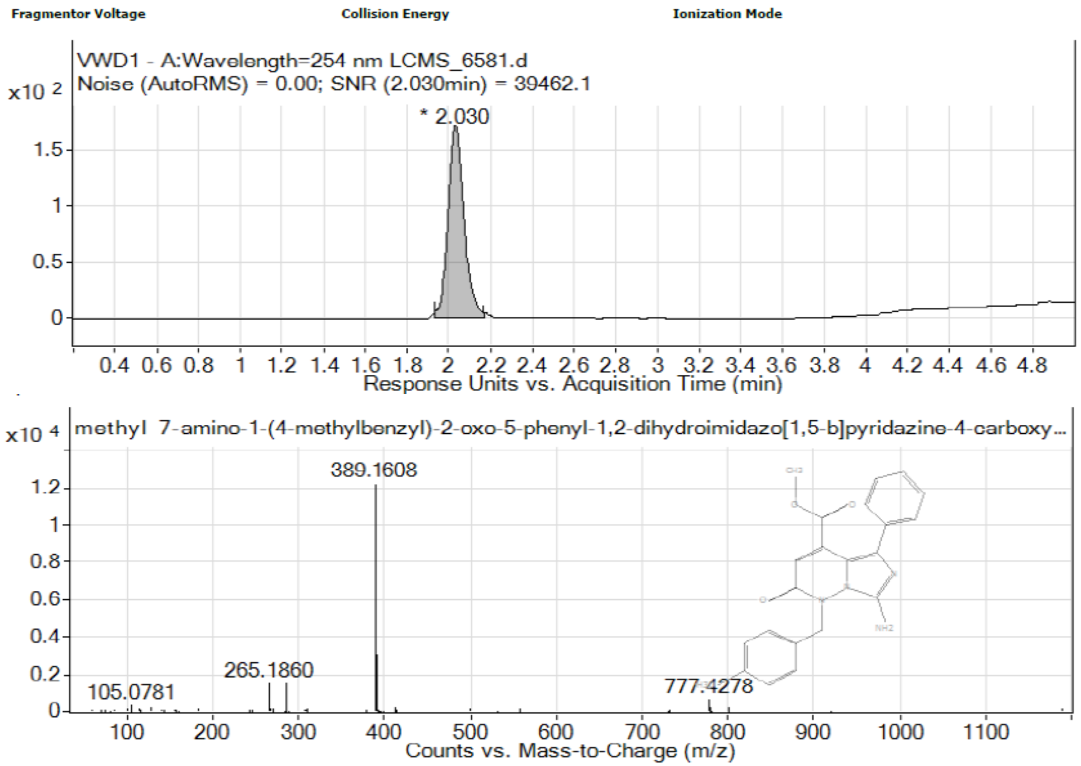

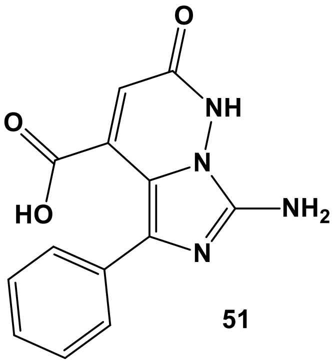

7-Amino-1,2-dihydro-2-oxo-5-phenylimidazo[1,5-b]pyridazine-4-carboxylic acid

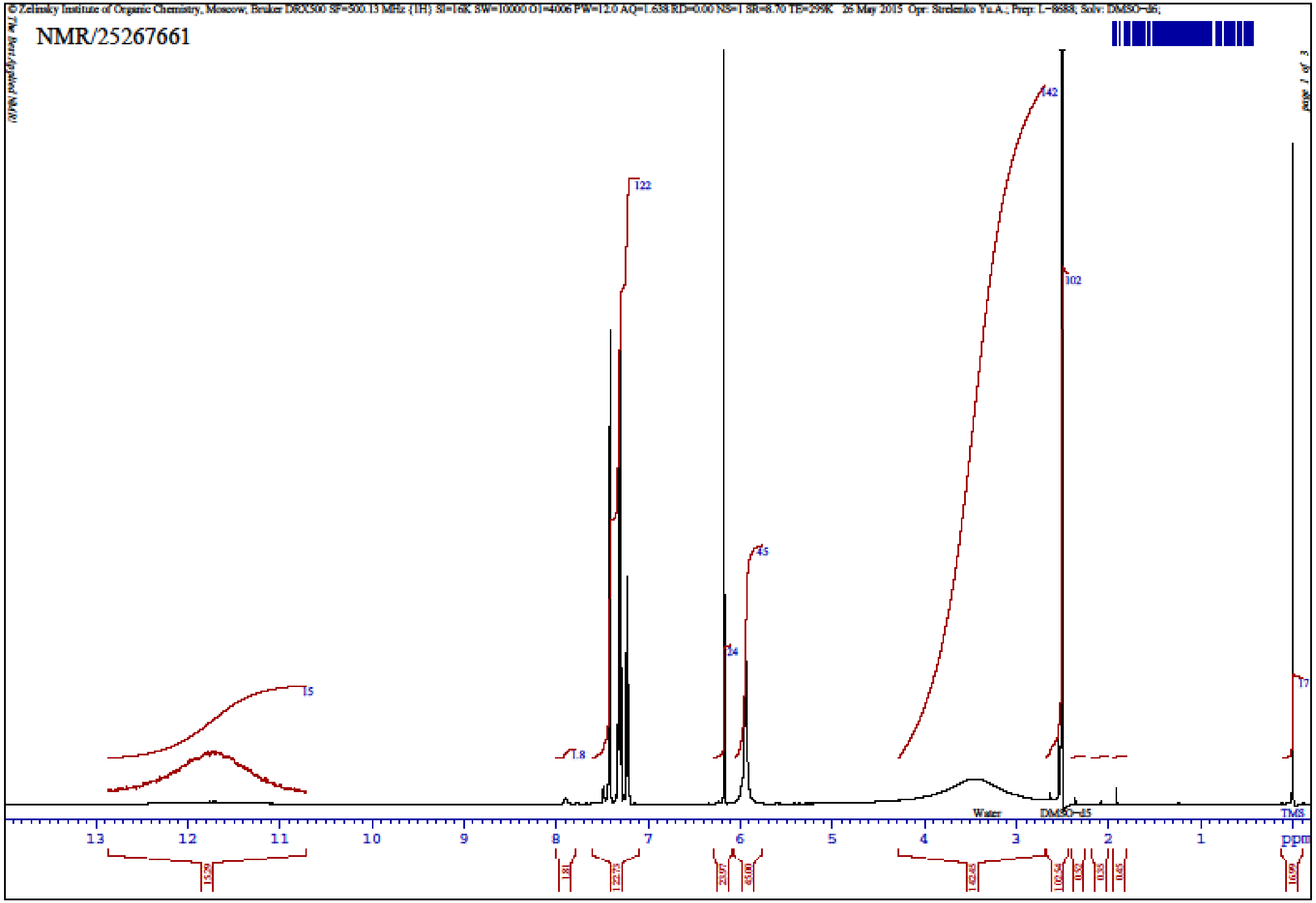

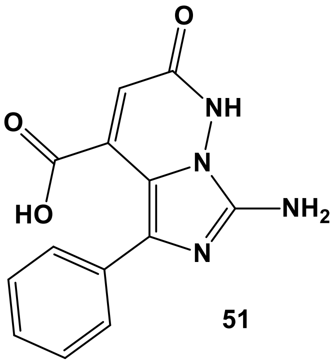

51

7-Amino-1,2-dihydro-2-oxo-5-phenylimidazo[1,5-b]pyridazine-4-carboxylic acid

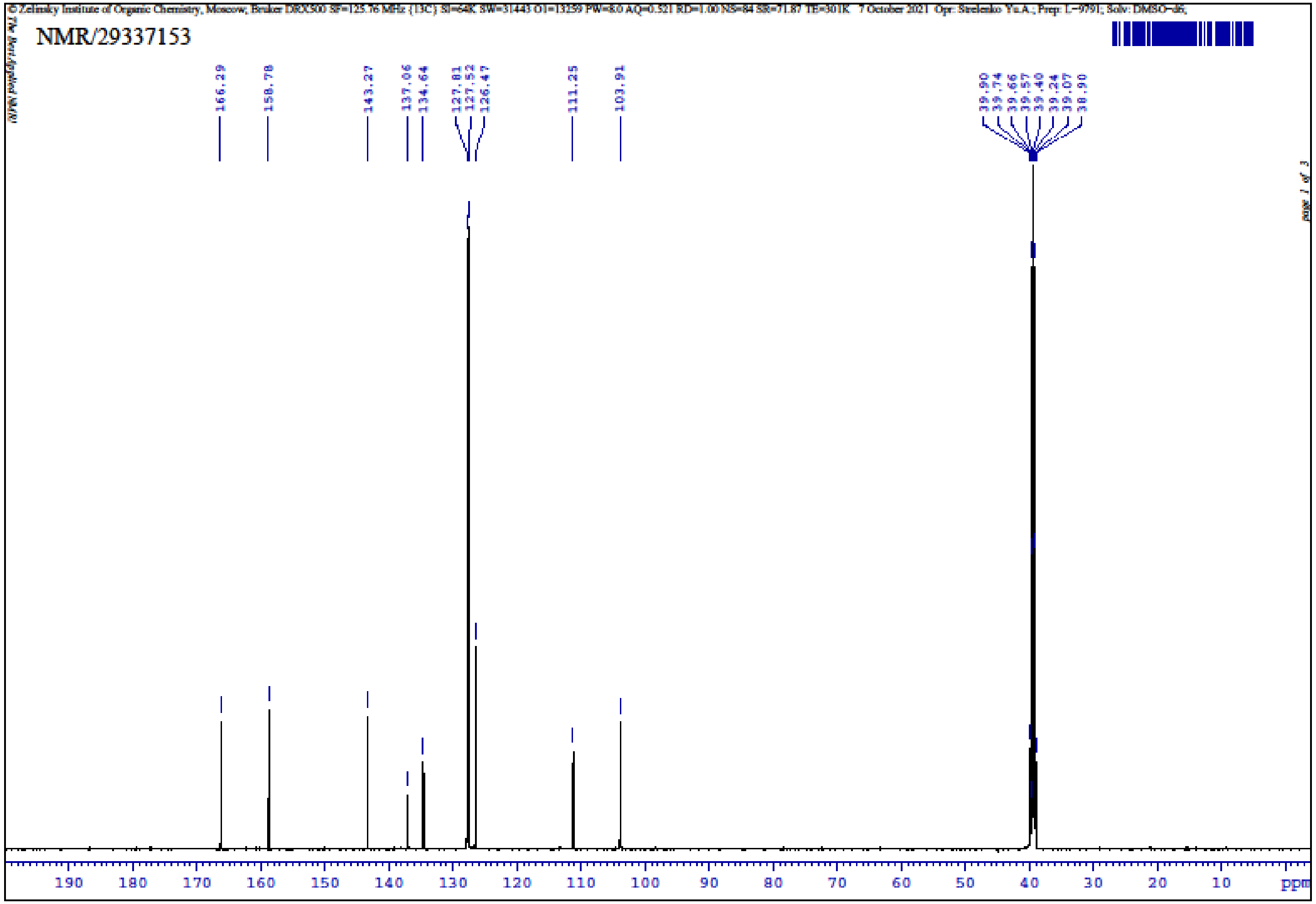

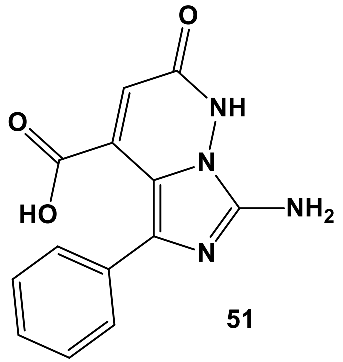 51<br>7-Amino-1,2-dihydro-2-oxo-5-phenylimidazo[1,5-b]pyridazine-4-carboxylic acid	Fragmentor Voltage Collision Energy Ionization Mode<br>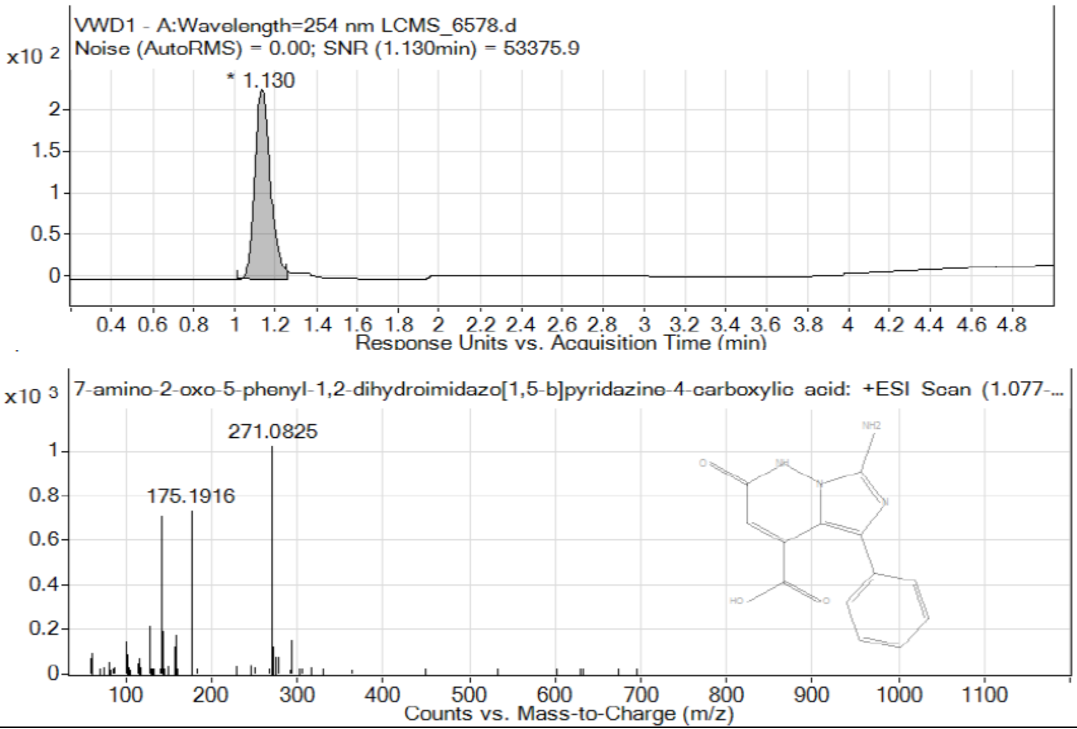

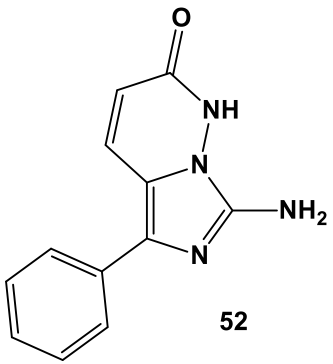

52

7-Amino-5-phenylimidazo[1,5-b]pyridazin-2(1H)-one

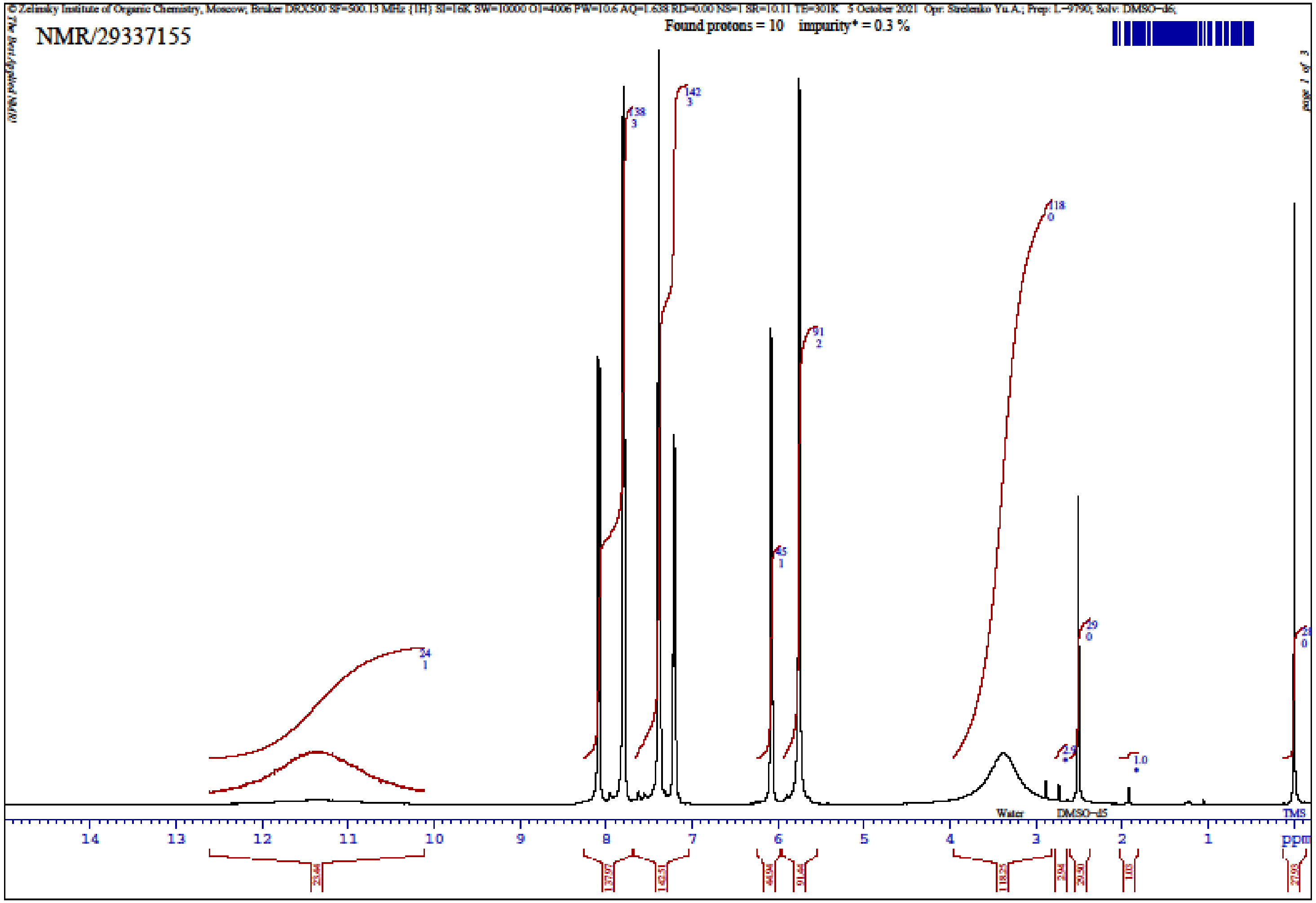

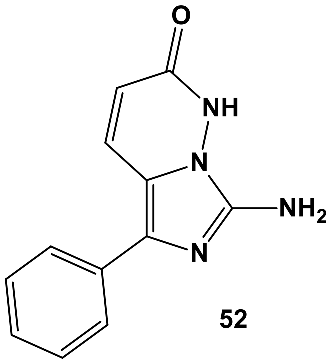

52

7-Amino-5-phenylimidazo[1,5-b]pyridazin-2(1H)-one

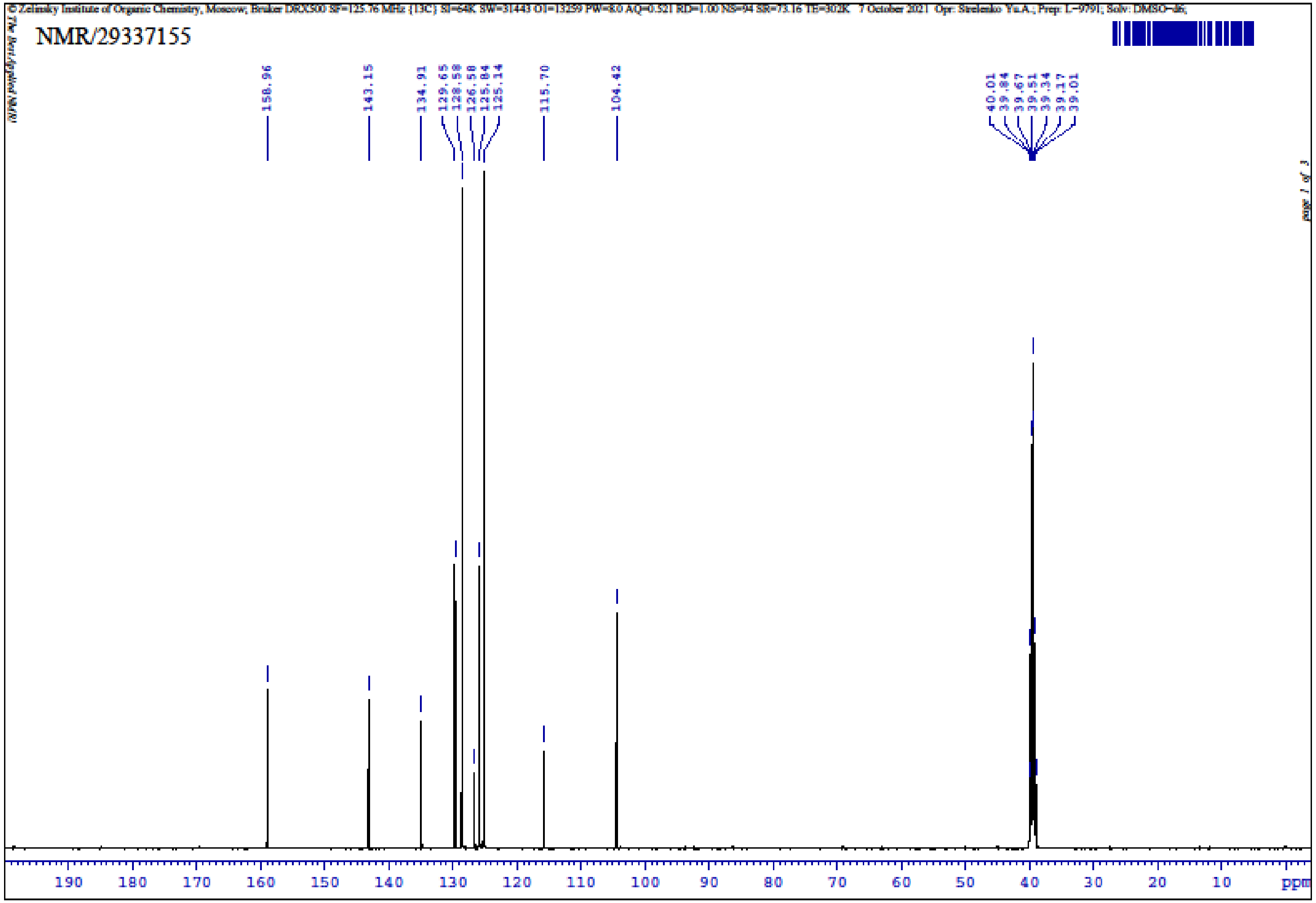

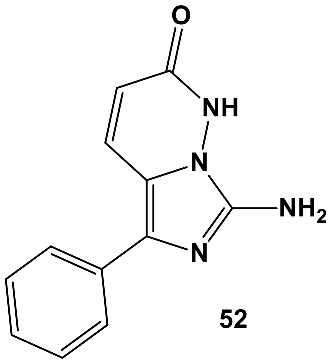

52

7-Amino-5-phenylimidazo[1,5-b]pyridazin-2(1H)-one

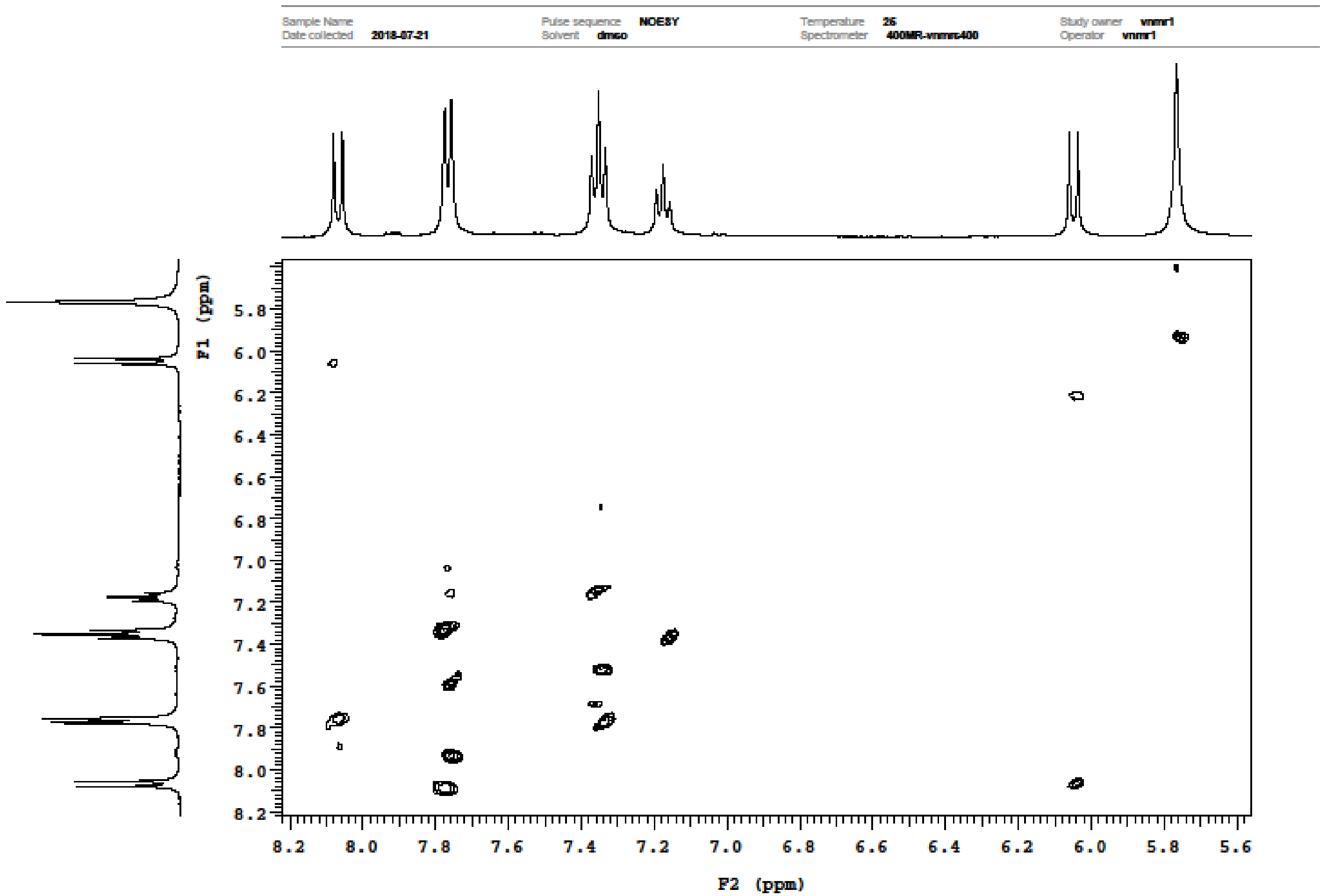

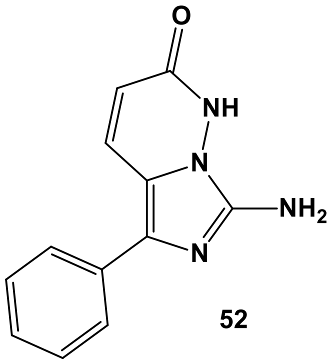

52

7-Amino-5-phenylimidazo[1,5-b]pyridazin-2(1H)-one

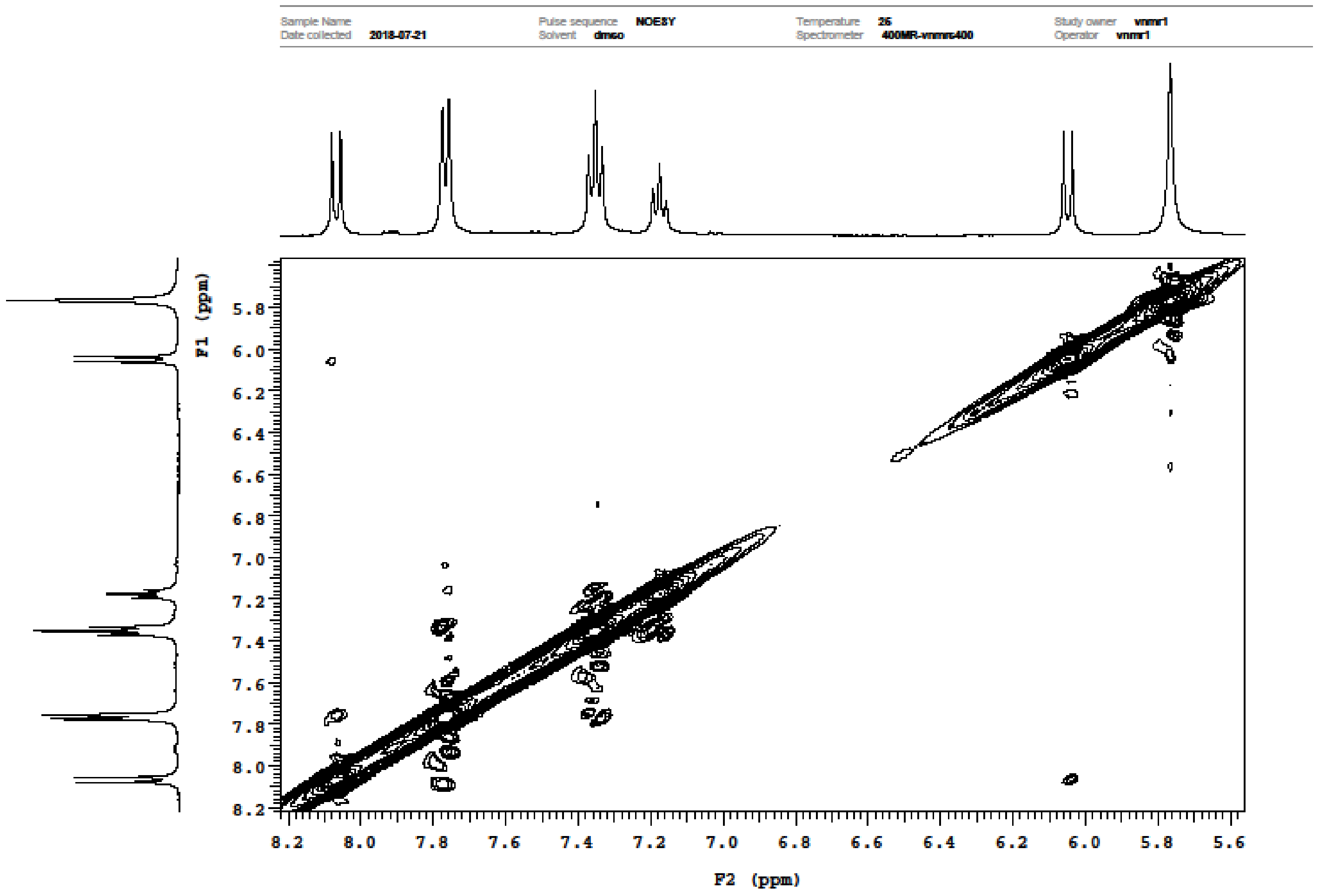

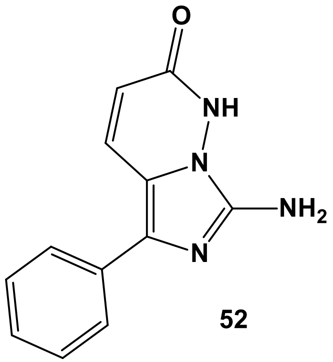 **52** 7-Amino-5-phenylimidazo[1,5-b]pyridazin-2(1H)-one	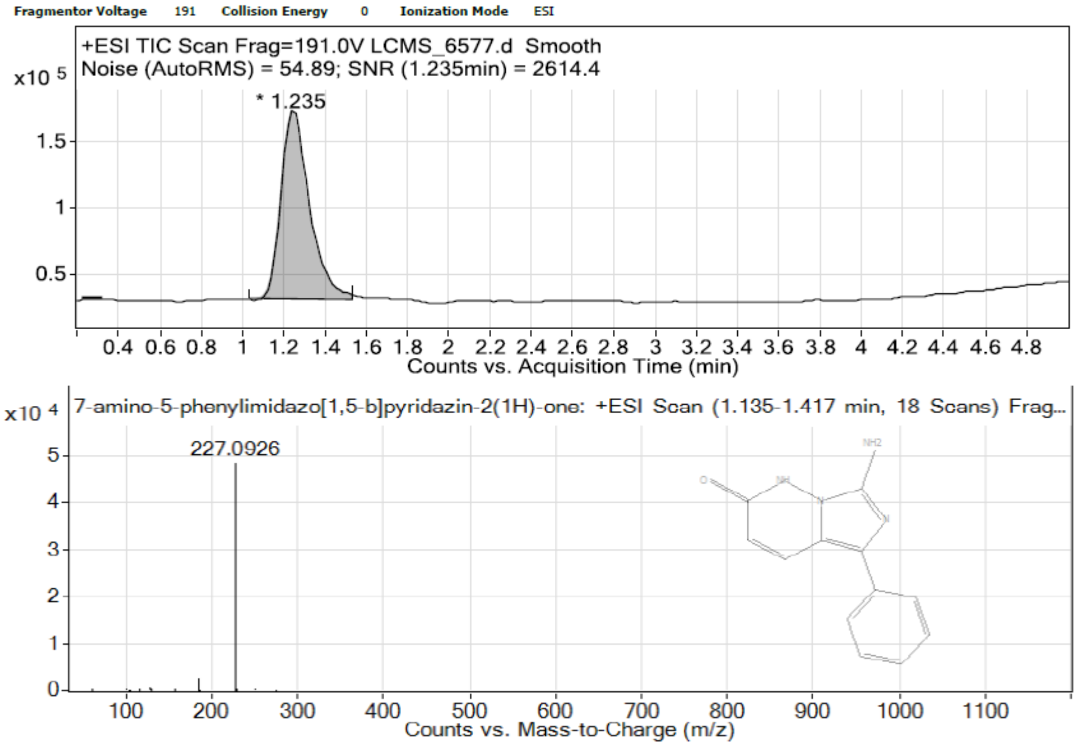

DFT calculation

Table S12. Optimized geometries of stationary points of the Scheme 9. PCM/B3LYP/6-311++G(d,p) calculations (Methanol, 64.7 °C). Coordinates in Angstroms. Sum of electronic and thermal Free Energies in a.u. are in parentheses.

1

$\nu_{im.}$ = 0 cm^{-1}(-533,18109)

6	-0.599208000	-0.188711000	-0.061238000
6	0.599187000	-0.188585000	0.061348000
6	-2.030004000	-0.248251000	-0.280073000
8	-2.540787000	-0.987031000	-1.091905000
8	-2.677402000	0.599821000	0.516534000
6	-4.123637000	0.629453000	0.391561000
1	-4.402662000	0.918512000	-0.620988000
1	-4.536455000	-0.349844000	0.630939000
1	-4.452831000	1.373742000	1.110535000
6	2.030000000	-0.248238000	0.280069000
8	2.540789000	-0.987001000	1.091906000
8	2.677410000	0.599781000	-0.516584000
6	4.123644000	0.629439000	-0.391590000
1	4.402662000	0.918436000	0.620979000
1	4.536490000	-0.349828000	-0.631026000
1	4.452832000	1.373782000	-1.110511000

44a

$\nu_{im.}$ = 0 (-567.98908)

7	-1.013234000	1.096078000	0.049545000
6	-2.270924000	0.714569000	0.036552000
6	-0.255253000	-0.074629000	0.002893000
6	-1.088173000	-1.163345000	-0.037888000
7	-2.377762000	-0.651835000	-0.016397000
1	-0.922994000	-2.225825000	-0.075669000
7	-3.534082000	-1.437705000	-0.086237000
1	-4.106209000	-1.133793000	-0.869455000
1	-4.068750000	-1.350885000	0.773630000
7	-3.377135000	1.540228000	-0.022346000
1	-3.152173000	2.501040000	0.199922000
1	-4.188434000	1.222280000	0.493142000
6	1.212397000	-0.039241000	0.004304000
6	1.895083000	1.186255000	-0.057576000
6	1.974219000	-1.219710000	0.064971000
6	3.288186000	1.229659000	-0.063239000
6	3.364854000	-1.174309000	0.057634000
6	4.032238000	0.051332000	-0.006622000
1	1.321130000	2.103202000	-0.103187000
1	1.477526000	-2.181873000	0.122100000
1	3.793351000	2.188275000	-0.112967000
1	3.930516000	-2.098445000	0.105374000
1	5.115773000	0.085162000	-0.011015000

TS1

$\nu_{im.}$ = -403.2cm^{-1}(-1101.11573)

7	1.965759000	1.484722000	-0.680306000
6	2.624938000	0.411457000	-1.147239000
6	0.654164000	1.154570000	-0.689395000
6	0.477263000	-0.215263000	-1.063511000
7	1.776963000	-0.595932000	-1.476265000
1	-0.344111000	-0.591593000	-1.651994000
7	2.101951000	-1.900251000	-1.849239000
1	2.679907000	-2.334851000	-1.133790000
1	2.572342000	-1.904710000	-2.748668000
7	3.961986000	0.324417000	-1.230047000
1	4.495324000	1.169521000	-1.096390000
1	4.397776000	-0.418767000	-1.753129000
6	-0.383864000	2.114060000	-0.322894000
6	-0.026547000	3.415123000	0.074102000
6	-1.746595000	1.770858000	-0.366693000
6	-1.003565000	4.342298000	0.418370000
6	-2.720196000	2.701758000	-0.020405000
6	-2.353935000	3.990186000	0.372790000
1	1.020878000	3.685799000	0.107156000
1	-2.050947000	0.775065000	-0.663302000
1	-0.713079000	5.341456000	0.722438000
1	-3.766682000	2.422000000	-0.057515000
1	-3.115025000	4.714223000	0.640268000
6	-1.106463000	-1.887446000	0.601921000
6	-0.039997000	-1.219709000	0.613061000
6	-2.238909000	-2.439178000	-0.039029000
8	-2.250631000	-3.490712000	-0.672846000
8	-3.359416000	-1.692090000	0.163292000
6	-4.582859000	-2.214160000	-0.391951000
1	-4.509105000	-2.300077000	-1.476728000
1	-4.812704000	-3.190511000	0.036788000
1	-5.351400000	-1.493426000	-0.122268000
6	0.998281000	-0.964282000	1.634387000
8	0.783029000	-0.448828000	2.709462000
8	2.211322000	-1.382632000	1.239250000
6	3.295873000	-1.202268000	2.183356000
1	3.412285000	-0.146134000	2.424327000
1	3.097762000	-1.771803000	3.090953000

54

$\nu_{im.}$ = 0 cm^{-1}(-1101.13298)

7	2.691232000	-0.800820000	-0.237211000
6	2.273625000	-2.080739000	-0.523370000
6	1.681346000	-0.007778000	-0.487790000
6	0.469587000	-0.753313000	-1.015950000
7	1.024250000	-2.123092000	-0.982066000
1	0.312661000	-0.472582000	-2.061764000
7	0.311257000	-3.232326000	-1.422261000
1	0.141429000	-3.885436000	-0.663410000
1	0.783540000	-3.686514000	-2.198353000
7	3.071663000	-3.120818000	-0.357345000
1	4.007235000	-2.969158000	-0.011840000
1	2.776897000	-4.063214000	-0.564171000
6	1.765915000	1.435909000	-0.314006000
6	2.874113000	1.992772000	0.352063000
6	0.766091000	2.286464000	-0.817112000
6	2.974282000	3.367454000	0.512638000
6	0.877018000	3.663883000	-0.658863000
6	1.976724000	4.205750000	0.006539000
1	3.641583000	1.336607000	0.741850000
1	-0.091853000	1.872589000	-1.331327000
1	3.826693000	3.789829000	1.031310000
1	0.105827000	4.314164000	-1.054037000
1	2.058239000	5.279425000	0.131408000
6	-1.946322000	-0.217311000	-1.071606000
6	-0.889468000	-0.584837000	-0.330153000
6	-3.288009000	-0.052213000	-0.619815000
8	-4.180257000	-0.901650000	-0.689169000
8	-3.566370000	1.240618000	-0.246693000
6	-4.938904000	1.538272000	0.054191000
1	-5.580146000	1.342872000	-0.807406000
1	-5.290736000	0.951323000	0.904521000
1	-4.960451000	2.598392000	0.301079000
6	-0.968702000	-0.838196000	1.135289000
8	-1.977165000	-0.791182000	1.812872000
8	0.242140000	-1.132640000	1.670723000
6	0.287856000	-1.371680000	3.091845000
1	-0.057395000	-0.492511000	3.636458000
1	-0.331709000	-2.230371000	3.352340000

1	4.180214000	-1.580818000	1.678371000	1	1.331671000	-1.573812000	3.320252000

**TS2**				**55**			
$\nu_{im.}$ = -1724.2cm^{-1}(-1101.09963)				$\nu_{im.}$ = 0 cm^{-1}(-1101.19909)			
7	2.744405000	-0.887713000	-0.723411000	7	2.125459000	-2.208112000	-0.111096000
6	2.283504000	-2.146844000	-0.850981000	6	1.146361000	-3.081825000	0.038982000
6	1.675448000	-0.106980000	-0.487021000	6	1.524104000	-0.970686000	-0.185246000
6	0.448778000	-0.864663000	-0.564539000	6	0.143468000	-1.084201000	-0.070217000
7	0.946735000	-2.212649000	-0.698204000	7	-0.078162000	-2.479383000	0.041089000
1	-0.327009000	-0.473402000	-1.613176000	1	-2.385019000	-1.267218000	-1.002472000
7	0.121908000	-3.317290000	-0.898121000	7	-1.286817000	-3.083845000	0.393698000
1	0.247919000	-3.996711000	-0.154529000	1	-1.171025000	-3.608540000	1.256290000
1	0.298702000	-3.742067000	-1.804238000	1	-1.603211000	-3.702296000	-0.347180000
7	3.060441000	-3.193334000	-1.172055000	7	1.304457000	-4.421915000	0.270347000
1	4.059031000	-3.053731000	-1.155730000	1	2.236398000	-4.760947000	0.075598000
1	2.724684000	-4.137323000	-1.057571000	1	0.578429000	-5.038307000	-0.069233000
6	1.849718000	1.328668000	-0.245187000	6	2.355472000	0.220300000	-0.433616000
6	3.141155000	1.822143000	0.018703000	6	3.563874000	0.379313000	0.264904000
6	0.776063000	2.235414000	-0.275782000	6	2.005131000	1.175326000	-1.397817000
6	3.349329000	3.176700000	0.249122000	6	4.386041000	1.474751000	0.019275000
6	0.991800000	3.592548000	-0.052085000	6	2.831474000	2.270421000	-1.644883000
6	2.275389000	4.068494000	0.213928000	6	4.022166000	2.425679000	-0.936824000
1	3.972326000	1.129853000	0.046192000	1	3.849143000	-0.360372000	1.003406000
1	-0.225122000	1.886951000	-0.485103000	1	1.093093000	1.052560000	-1.968850000
1	4.349689000	3.538757000	0.457150000	1	5.311584000	1.587515000	0.572818000
1	0.153852000	4.279222000	-0.086291000	1	2.547772000	2.996773000	-2.398164000
1	2.438404000	5.125122000	0.393055000	1	4.664975000	3.276896000	-1.130889000
6	-1.709643000	-0.255395000	-0.938096000	6	-2.174019000	-0.349882000	-0.474134000
6	-0.886135000	-0.617222000	0.066385000	6	-0.919105000	-0.105245000	-0.024803000
6	-3.125099000	0.080248000	-0.928440000	6	-3.330071000	0.528603000	-0.253963000
8	-4.027712000	-0.721232000	-1.121269000	8	-3.414394000	1.435395000	0.553864000
8	-3.334631000	1.413095000	-0.816684000	8	-4.350135000	0.174221000	-1.067933000
6	-4.701215000	1.864550000	-0.928232000	6	-5.569239000	0.933755000	-0.940300000
1	-5.117360000	1.587162000	-1.897377000	1	-5.966401000	0.846741000	0.071588000
1	-5.311385000	1.437954000	-0.131467000	1	-5.390249000	1.983588000	-1.174476000
1	-4.656329000	2.946691000	-0.831086000	1	-6.258953000	0.497041000	-1.658263000
6	-1.210903000	-0.749042000	1.504999000	6	-0.590926000	1.236763000	0.601806000
8	-2.301312000	-0.524389000	1.993827000	8	-0.594943000	2.290653000	0.012086000
8	-0.145066000	-1.152819000	2.222965000	8	-0.273391000	1.093473000	1.893834000
6	-0.344618000	-1.312480000	3.644622000	6	0.054697000	2.304345000	2.615839000
1	-0.639333000	-0.363836000	4.093782000	1	0.924759000	2.783745000	2.167780000
1	-1.109956000	-2.064662000	3.837101000	1	-0.793754000	2.988363000	2.604556000
1	0.615801000	-1.637161000	4.036732000	1	0.274249000	1.982804000	3.630394000

**TS3**				**45a**			
$\nu_{im.}$ = -677.4cm^{-1}(-1101.12660)				$\nu_{im.}$ = 0 cm^{-1}(-1101.19909)			
7	0.394072000	2.503318000	-0.217839000	7	-0.304793000	-2.427925000	0.072134000
6	-0.908305000	2.505474000	-0.427636000	6	-1.626974000	-2.302706000	0.067182000
6	0.778347000	1.185479000	-0.078929000	6	0.222553000	-1.166607000	-0.016633000
6	-0.311880000	0.341817000	-0.251632000	6	-0.807746000	-0.222255000	-0.040252000
7	-1.383526000	1.225350000	-0.477085000	7	-1.977014000	-0.997448000	0.018524000
1	-2.003346000	-2.635679000	-0.325496000	1	-2.391271000	2.789080000	-0.280071000
7	-2.731389000	0.831726000	-0.535448000	7	-3.255617000	-0.468005000	-0.055863000
1	-3.197280000	1.200183000	-1.366903000	1	-3.971200000	-1.013583000	0.412729000
1	-3.246558000	0.913159000	0.451480000	7	-2.552562000	-3.313380000	0.187958000
7	-1.690818000	3.600858000	-0.662574000	1	-2.123442000	-4.229704000	0.167936000
1	-1.228700000	4.476369000	-0.456582000	1	-3.339935000	-3.264568000	-0.449546000
1	-2.644399000	3.575028000	-0.324754000	6	1.677385000	-0.980409000	-0.137763000
6	2.170346000	0.868543000	0.277815000	6	2.545112000	-1.713830000	0.685459000
6	3.224451000	1.542429000	-0.356773000	6	2.226743000	-0.121828000	-1.100859000
6	2.471022000	-0.058410000	1.286175000	6	3.924865000	-1.573229000	0.562888000
6	4.545776000	1.275051000	-0.009029000	6	3.607985000	0.018073000	-1.221650000
6	3.794188000	-0.324349000	1.633029000	6	4.461896000	-0.704368000	-0.388621000
6	4.835958000	0.338614000	0.984783000	1	2.130304000	-2.387744000	1.425511000
1	3.001126000	2.269537000	-1.128226000	1	1.574739000	0.415729000	-1.780010000
1	1.668560000	-0.551596000	1.822772000	1	4.582143000	-2.141209000	1.211749000
1	5.350310000	1.797555000	-0.514270000	1	4.015736000	0.681204000	-1.976212000
1	4.009186000	-1.039217000	2.419167000	1	5.536364000	-0.597441000	-0.484592000
1	5.865121000	0.133594000	1.256740000	6	-2.240083000	1.720510000	-0.219736000
6	-1.834124000	-1.567381000	-0.298051000	6	-0.988551000	1.188798000	-0.120934000
6	-0.574752000	-1.068612000	-0.252398000	6	-3.445156000	0.908977000	-0.110284000
6	-3.039316000	-0.760700000	-0.448859000	8	-4.585456000	1.361116000	-0.043492000
8	-4.076345000	-1.080110000	-0.983052000	6	0.158881000	2.160401000	-0.041519000

8	-3.443826000	-0.176455000	1.414347000	8	0.325337000	3.065745000	-0.826504000
6	-4.671938000	-0.658896000	1.890381000	8	0.913108000	1.931141000	1.031817000
1	-5.271227000	-1.088396000	1.070807000	6	2.031783000	2.827927000	1.245089000
1	-4.527509000	-1.435698000	2.653950000	1	2.726661000	2.758164000	0.408864000
1	-5.265913000	0.150271000	2.337094000	1	1.673871000	3.851343000	1.352186000
6	0.531319000	-2.087653000	-0.309276000	1	2.499858000	2.485287000	2.163415000
8	0.555635000	-3.092625000	0.365649000				
8	1.432647000	-1.781614000	-1.243628000				
6	2.529108000	-2.711340000	-1.416989000				
1	3.106564000	-2.780227000	-0.495511000				
1	2.146714000	-3.693377000	-1.693841000				
1	3.132912000	-2.294252000	-2.218009000				

**CH_3OH**			
$\nu_{im.}$ = 0cm^{-1}(-115.74626)			
1	1.146969000	-0.755172000	0.000004000
8	0.752515000	0.122872000	0.000001000
6	-0.671353000	-0.019805000	0.000000000
1	-1.090218000	0.986428000	-0.000251000
1	-1.024356000	-0.547914000	-0.891719000
1	-1.024395000	-0.547487000	0.891959000

Table S13. Optimized geometries of stationary points of the Scheme 10. PCM/B3LYP/6-311++G(d,p) calculations (Methanol, 64.7 °C). Coordinates in Angstroms. Sum of electronic and thermal Free Energies in a.u. are in parentheses.

**TS4**				**56**			
$\nu_{im.}$ = -249.5cm^{-1}(-1101.11891)				$\nu_{im.}$ = 0 cm^{-1}(-1101.13791)			
7	0.224306000	0.742692000	-0.205663000	7	-0.415246000	1.099756000	-0.224734000
6	0.846818000	1.915531000	-0.238670000	6	-0.546113000	2.439113000	-0.252653000
6	-1.045676000	0.925784000	-0.764555000	6	-1.684410000	0.514714000	-0.442853000
6	-1.168321000	2.230428000	-1.142945000	6	-2.563915000	1.536967000	-0.582798000
7	0.030491000	2.851759000	-0.808362000	7	-1.850558000	2.727929000	-0.462793000
1	-1.943451000	2.774018000	-1.654015000	1	-3.617242000	1.548183000	-0.797022000
7	0.301635000	4.198813000	-1.059986000	7	-2.433186000	3.989628000	-0.586623000
1	1.074726000	4.289985000	-1.712881000	1	-2.048316000	4.482542000	-1.387041000
1	0.515903000	4.684226000	-0.193914000	1	-2.298194000	4.527645000	0.264110000
7	2.073921000	2.210621000	0.277096000	7	0.439208000	3.330115000	-0.042810000
1	2.743080000	1.443276000	0.316335000	1	1.370643000	2.984772000	-0.250660000
1	2.485020000	3.078799000	-0.037257000	1	0.261168000	4.294349000	-0.285368000
6	-2.008371000	-0.168462000	-0.933246000	6	-1.934643000	-0.929714000	-0.532784000
6	-1.576968000	-1.495884000	-1.082000000	6	-1.073529000	-1.783243000	-1.239262000
6	-3.387807000	0.095867000	-0.974149000	6	-3.091126000	-1.465844000	0.054472000
6	-2.497434000	-2.525804000	-1.267140000	6	-1.363970000	-3.141324000	-1.348968000
6	-4.303868000	-0.933619000	-1.169254000	6	-3.382272000	-2.821780000	-0.067855000
6	-3.863833000	-2.250770000	-1.314153000	6	-2.517839000	-3.665153000	-0.766431000
1	-0.517455000	-1.718031000	-1.065209000	1	-0.185302000	-1.384635000	-1.712366000
1	-3.744124000	1.110787000	-0.838117000	1	-3.754014000	-0.819818000	0.618244000
1	-2.144197000	-3.544577000	-1.381898000	1	-0.690674000	-3.788650000	-1.899130000
1	-5.364335000	-0.709002000	-1.196387000	1	-4.278771000	-3.221141000	0.392481000
1	-4.578632000	-3.052728000	-1.459064000	1	-2.741124000	-4.722159000	-0.854774000
6	0.892196000	-0.648378000	1.116621000	6	0.853872000	0.430527000	0.076980000
6	2.002600000	-1.165499000	0.864844000	6	1.910460000	0.671795000	-0.695668000
6	-0.146862000	-0.680216000	2.160813000	6	0.854096000	-0.359110000	1.343789000
8	-0.315194000	-1.660215000	2.857749000	8	1.729287000	-1.140208000	1.658483000
8	-0.828236000	0.457293000	2.295523000	8	-0.203995000	-0.084717000	2.132104000
6	-1.830152000	0.480744000	3.338533000	6	-0.267081000	-0.784382000	3.393362000
1	-2.576037000	-0.293493000	3.160298000	1	-0.290853000	-1.861607000	3.228255000
1	-1.363709000	0.329348000	4.311904000	1	0.592447000	-0.526032000	4.012374000
1	-2.280369000	1.467873000	3.278984000	1	-1.188398000	-0.450194000	3.863797000
6	3.143199000	-1.101996000	0.021129000	6	3.236036000	0.195225000	-0.447689000
8	3.950960000	-0.175076000	0.041043000	8	4.095682000	0.788896000	0.203024000
8	3.266686000	-2.170161000	-0.787940000	8	3.525676000	-0.940636000	-1.150319000
6	4.394872000	-2.169145000	-1.691610000	6	4.882278000	-1.412708000	-1.076681000
1	4.348726000	-1.308057000	-2.358501000	1	5.577306000	-0.660625000	-1.453880000
1	5.329292000	-2.152875000	-1.130586000	1	5.150263000	-1.671352000	-0.051027000
1	4.307004000	-3.092958000	-2.257605000	1	4.915269000	-2.300459000	-1.705366000

**TS5**	**57**
$\nu_{im.}$ = -1307.1cm^{-1}(-1101.13233)	$\nu_{im.}$ = 0 cm^{-1}(-1101.16720)

7	-0.139164000	1.152850000	-0.041770000	7	-0.176435000	1.133340000	-0.236194000
6	0.139502000	2.496280000	-0.016541000	6	0.026256000	2.529664000	-0.147817000
6	-1.546027000	0.984234000	-0.145958000	6	-1.576515000	0.856886000	-0.270773000
6	-2.090332000	2.222853000	-0.094012000	6	-2.211250000	2.043751000	-0.177905000
7	-1.054433000	3.151026000	0.005733000	7	-1.258892000	3.051084000	-0.078582000
1	-3.111867000	2.546447000	-0.181293000	1	-3.259166000	2.278351000	-0.243995000
7	-1.245840000	4.532276000	-0.024985000	7	-1.579904000	4.405433000	-0.051401000
1	-0.808721000	4.927363000	-0.853930000	1	-1.193070000	4.874752000	-0.867109000
1	-0.847370000	4.956344000	0.807861000	1	-1.207257000	4.835579000	0.790992000
7	1.365248000	2.967899000	-0.057748000	7	1.162865000	3.125528000	-0.125612000
1	2.081921000	2.018323000	-0.258582000	1	2.199905000	1.102251000	-1.303430000
1	1.515570000	3.965452000	0.009259000	1	1.039176000	4.124546000	0.022189000
6	-2.235165000	-0.285726000	-0.412811000	6	-2.156191000	-0.466777000	-0.527322000
6	-1.754017000	-1.183431000	-1.378204000	6	-1.629068000	-1.315287000	-1.514949000
6	-3.438866000	-0.579435000	0.244762000	6	-3.302782000	-0.875914000	0.172252000
6	-2.458371000	-2.349160000	-1.670713000	6	-2.235077000	-2.537039000	-1.793244000
6	-4.146510000	-1.739248000	-0.060528000	6	-3.913271000	-2.093357000	-0.118903000
6	-3.657180000	-2.630891000	-1.015644000	6	-3.380283000	-2.930376000	-1.099526000
1	-0.839075000	-0.962975000	-1.915743000	1	-0.753073000	-1.012509000	-2.076632000
1	-3.811251000	0.097724000	1.004618000	1	-3.707036000	-0.239734000	0.951104000
1	-2.074428000	-3.031933000	-2.420094000	1	-1.817135000	-3.178965000	-2.560404000
1	-5.075136000	-1.952037000	0.456887000	1	-4.798718000	-2.393461000	0.430007000
1	-4.204791000	-3.537250000	-1.246980000	1	-3.851116000	-3.881609000	-1.319530000
6	0.916051000	0.173153000	0.059939000	6	0.841222000	0.198853000	0.006396000
6	2.165099000	0.546524000	-0.247850000	6	2.053825000	0.334566000	-0.557675000
6	0.612418000	-1.113760000	0.760885000	6	0.527040000	-0.910794000	0.989694000
8	1.176527000	-2.161218000	0.523057000	8	0.722594000	-2.083754000	0.784309000
8	-0.275315000	-0.954739000	1.754035000	8	0.004393000	-0.406910000	2.111395000
6	-0.597956000	-2.131271000	2.529022000	6	-0.352746000	-1.358825000	3.142859000
1	-1.020275000	-2.901625000	1.883981000	1	-1.106546000	-2.049805000	2.766189000
1	0.294765000	-2.511411000	3.025493000	1	0.530903000	-1.909181000	3.464747000
1	-1.330797000	-1.801875000	3.260821000	1	-0.751564000	-0.761582000	3.958073000
6	3.349862000	-0.265403000	-0.051453000	6	3.255558000	-0.437635000	-0.182572000
8	3.996310000	-0.331371000	0.985483000	8	3.478891000	-0.953114000	0.893533000
8	3.762901000	-0.856411000	-1.200486000	8	4.127249000	-0.457448000	-1.209797000
6	5.001301000	-1.591925000	-1.137552000	6	5.375504000	-1.147265000	-0.983231000
1	5.823079000	-0.937011000	-0.845084000	1	5.915085000	-0.688096000	-0.154577000
1	4.922885000	-2.417543000	-0.429187000	1	5.192324000	-2.199652000	-0.765603000
1	5.162564000	-1.973199000	-2.143398000	1	5.935503000	-1.041468000	-1.908712000

Table S14. Optimized geometries of stationary points of the Scheme 11. PCM/B3LYP/6-311++G(d,p) calculations (Methanol, 64.7 °C). Coordinates in Angstroms. Sum of electronic and thermal Free Energies in a.u. are in parentheses.

TS6				58			
$\nu_{im.}$ = -223.0cm^{-1}(-1101.11271)				$\nu_{im.}$ = 0 cm^{-1}(-1101.11690)			
7	-1.213953000	1.111637000	0.096488000	7	-1.343920000	0.504050000	-0.702693000
6	-0.201891000	1.098583000	-0.731139000	6	-0.328914000	-0.097066000	-1.254666000
6	-2.234644000	0.427575000	-0.547975000	6	-2.280991000	-0.493622000	-0.480061000
6	-1.788306000	-0.004803000	-1.777446000	6	-1.770391000	-1.700316000	-0.912752000
7	-0.483352000	0.423996000	-1.883021000	7	-0.516263000	-1.434333000	-1.412585000
1	-2.240597000	-0.569365000	-2.573981000	1	-2.157921000	-2.704139000	-0.925837000
7	0.335164000	0.168280000	-2.991300000	7	0.356670000	-2.405457000	-1.932281000
1	1.190174000	-0.280015000	-2.673439000	1	1.291628000	-2.197182000	-1.553357000
1	0.551503000	1.035888000	-3.474293000	1	0.363858000	-2.336440000	-2.947679000
7	1.068881000	1.644864000	-0.449946000	7	0.896867000	0.579088000	-1.617834000
1	0.995133000	2.304813000	0.322576000	1	0.683600000	1.583077000	-1.683953000
1	1.482845000	2.137934000	-1.240109000	1	1.222070000	0.277867000	-2.540847000
6	-3.553617000	0.244347000	0.073647000	6	-3.587452000	-0.203488000	0.127377000
6	-3.783293000	0.670982000	1.390653000	6	-3.966899000	1.120421000	0.395434000
6	-4.611314000	-0.358783000	-0.626891000	6	-4.483292000	-1.235311000	0.451112000
6	-5.029967000	0.497709000	1.988145000	6	-5.205095000	1.403229000	0.967824000
6	-5.855052000	-0.533053000	-0.026977000	6	-5.720451000	-0.949851000	1.021499000
6	-6.072265000	-0.105497000	1.284318000	6	-6.088847000	0.371108000	1.282946000
1	-2.976484000	1.137606000	1.941766000	1	-3.284788000	1.925117000	0.151310000
1	-4.466727000	-0.691857000	-1.648342000	1	-4.214217000	-2.268433000	0.262876000
1	-5.186327000	0.833955000	3.007243000	1	-5.479976000	2.433166000	1.166793000
1	-6.657804000	-1.001090000	-0.585820000	1	-6.397314000	-1.761119000	1.265029000
1	-7.041865000	-0.240590000	1.749815000	1	-7.052153000	0.591776000	1.728326000
6	2.315628000	0.344370000	0.055933000	6	2.072667000	0.403688000	-0.618455000
6	1.967984000	-0.849290000	-0.160294000	6	2.556098000	-0.812234000	-0.457700000

6	3.462995000	1.096440000	0.591782000	6	2.480654000	1.690862000	-0.006028000
8	4.439624000	0.542167000	1.047355000	8	3.354968000	1.800310000	0.823733000
8	3.318822000	2.428385000	0.521052000	8	1.769005000	2.735445000	-0.485548000
6	4.413637000	3.228999000	1.028746000	6	2.081279000	4.047703000	0.040356000
1	4.569625000	3.018327000	2.086188000	1	1.909176000	4.066548000	1.115863000
1	5.322304000	3.015913000	0.466799000	1	3.119105000	4.297042000	-0.177637000
1	4.104783000	4.260407000	0.883712000	1	1.406288000	4.729652000	-0.469104000
6	2.398278000	-2.198028000	-0.103949000	6	3.681184000	-1.143796000	0.372567000
8	2.992266000	-2.781027000	-1.003508000	8	4.841789000	-1.206374000	-0.017338000
8	2.028257000	-2.806153000	1.052172000	8	3.310770000	-1.517455000	1.625609000
6	2.375166000	-4.200684000	1.180071000	6	4.361329000	-1.987673000	2.493261000
1	1.905747000	-4.787083000	0.389440000	1	4.844241000	-2.869912000	2.070817000
1	3.456814000	-4.333807000	1.142641000	1	5.105475000	-1.207291000	2.657027000
1	1.991900000	-4.502659000	2.151973000	1	3.869916000	-2.240504000	3.430202000

TS7				59			
$\nu_{im.}$ = -1760.2cm^{-1}(-1101.09278)				$\nu_{im.}$ = 0 cm^{-1}(-1101.19391)			
7	1.532893000	0.048422000	0.867942000	7	1.387294000	0.024973000	0.908534000
6	0.354908000	-0.461169000	0.624021000	6	0.402784000	-0.830579000	0.758722000
6	2.419958000	-0.634950000	0.049344000	6	2.486070000	-0.536681000	0.284218000
6	1.727517000	-1.568185000	-0.688885000	6	2.132099000	-1.759283000	-0.250869000
7	0.406451000	-1.455626000	-0.312945000	7	0.811425000	-1.943610000	0.064162000
1	2.020496000	-2.297499000	-1.423918000	1	2.669363000	-2.501402000	-0.815691000
7	-0.602144000	-2.299906000	-0.803515000	7	0.069705000	-3.077605000	-0.303290000
1	-1.342197000	-1.741244000	-1.217517000	1	-0.720730000	-2.766421000	-0.866643000
1	-0.990372000	-2.843801000	-0.037988000	1	-0.287061000	-3.518406000	0.540485000
7	-0.839187000	-0.065305000	1.293659000	7	-0.893192000	-0.709642000	1.279534000
1	-0.572285000	0.585039000	2.035474000	1	-0.989002000	-0.793767000	2.283983000
1	-1.745360000	-0.877699000	1.678304000	1	-3.289815000	-0.157215000	2.252191000
6	3.858211000	-0.333360000	0.045577000	6	3.788121000	0.145149000	0.244621000
6	4.396997000	0.568047000	0.976259000	6	3.950275000	1.402089000	0.847473000
6	4.725341000	-0.935429000	-0.881007000	6	4.896896000	-0.437305000	-0.391506000
6	5.760278000	0.855560000	0.981654000	6	5.180849000	2.054521000	0.815182000
6	6.087194000	-0.648203000	-0.872155000	6	6.125298000	0.216670000	-0.423016000
6	6.613039000	0.249251000	0.059262000	6	6.275100000	1.466833000	0.180217000
1	3.738848000	1.039770000	1.695157000	1	3.103246000	1.862018000	1.340870000
1	4.336415000	-1.628797000	-1.618120000	1	4.803912000	-1.407794000	-0.865725000
1	6.156946000	1.554655000	1.709638000	1	5.284400000	3.025352000	1.287336000
1	6.739066000	-1.123403000	-1.596717000	1	6.967974000	-0.251300000	-0.919758000
1	7.673604000	0.473402000	0.063878000	1	7.232281000	1.975057000	0.155064000
6	-2.065033000	0.384271000	0.544612000	6	-1.986866000	-0.245525000	0.592457000
6	-3.017053000	-0.456281000	0.955307000	6	-3.182993000	-0.013189000	1.184078000
6	-2.082806000	1.569943000	-0.336232000	6	-1.771691000	-0.075060000	-0.901145000
8	-3.064324000	1.925732000	-0.954128000	8	-1.729647000	-1.013650000	-1.666733000
8	-0.900973000	2.199417000	-0.357419000	8	-1.566904000	1.191963000	-1.223172000
6	-0.810249000	3.386033000	-1.181731000	6	-1.357956000	1.481925000	-2.630008000
1	-1.015550000	3.135159000	-2.222128000	1	-0.490260000	0.936266000	-2.998409000
1	-1.517787000	4.137946000	-0.833155000	1	-2.246923000	1.204776000	-3.195464000
1	0.211280000	3.737293000	-1.066003000	1	-1.189203000	2.553748000	-2.676493000
6	-4.442171000	-0.427999000	0.656229000	6	-4.362768000	0.396763000	0.438470000
8	-5.273289000	0.124570000	1.356399000	8	-4.449841000	0.518906000	-0.773766000
8	-4.752202000	-1.168972000	-0.425482000	8	-5.404445000	0.628659000	1.270958000
6	-6.156176000	-1.284593000	-0.750688000	6	-6.643680000	1.031006000	0.654388000
1	-6.700593000	-1.742229000	0.075557000	1	-6.515345000	1.971801000	0.118043000
1	-6.577059000	-0.303721000	-0.972115000	1	-6.994891000	0.261966000	-0.034425000
1	-6.194955000	-1.921561000	-1.630710000	1	-7.347104000	1.155486000	1.474065000

Table S15. Optimized geometries of stationary points of the Scheme 12. PCM/B3LYP/6-311++G(d,p) calculations (Methanol, 64.7 °C). Coordinates in Angstroms. Sum of electronic and thermal Free Energies in a.u. are in parentheses.

TS8				60			
$\nu_{im.}$ = -201.9cm^{-1}(-1101.10937)				$\nu_{im.}$ = 0 cm^{-1}(-1101.11532)			
7	2.410478000	-1.484710000	-0.685771000	7	2.264143000	-1.698926000	-0.310565000
6	1.206983000	-1.879664000	-1.022181000	6	1.046501000	-2.056407000	-0.616379000
6	2.329682000	-0.112533000	-0.463202000	6	2.286044000	-0.302693000	-0.313145000
6	1.039821000	0.318708000	-0.657581000	6	1.043086000	0.190027000	-0.615372000
7	0.339876000	-0.816272000	-1.036158000	7	0.254604000	-0.942945000	-0.791742000
1	0.555077000	1.274948000	-0.565941000	1	0.629022000	1.180666000	-0.680136000
7	-1.018501000	-0.868594000	-1.379402000	7	-1.040128000	-0.967986000	-1.380256000
1	-1.202722000	-1.797277000	-1.767728000	1	-1.242277000	-1.961782000	-1.564383000

1	-1.219625000	-0.176419000	-2.105120000	1	-1.017179000	-0.472829000	-2.282062000
7	0.785711000	-3.139297000	-1.420729000	7	0.526237000	-3.318560000	-0.844618000
1	1.568881000	-3.767959000	-1.550982000	1	1.252390000	-4.021806000	-0.904707000
1	0.101623000	-3.561311000	-0.800972000	1	-0.185527000	-3.598727000	-0.177425000
6	3.506981000	0.670264000	-0.064332000	6	3.513871000	0.441078000	0.001953000
6	4.749663000	0.041223000	0.108326000	6	4.706571000	-0.246873000	0.270872000
6	3.424703000	2.056085000	0.153116000	6	3.528023000	1.845298000	0.035927000
6	5.872775000	0.774043000	0.486903000	6	5.878346000	0.448709000	0.562368000
6	4.547319000	2.785388000	0.532514000	6	4.699171000	2.537240000	0.328051000
6	5.778850000	2.148837000	0.701794000	6	5.881599000	1.842810000	0.592704000
1	4.825599000	-1.026131000	-0.056330000	1	4.706827000	-1.329217000	0.249217000
1	2.479406000	2.571388000	0.025993000	1	2.621257000	2.404298000	-0.165215000
1	6.823574000	0.268369000	0.614921000	1	6.790329000	-0.101109000	0.766966000
1	4.461224000	3.853917000	0.695690000	1	4.689187000	3.621203000	0.349697000
1	6.652543000	2.718526000	0.997105000	1	6.793042000	2.383559000	0.820542000
6	-3.117564000	0.490569000	-0.589082000	6	-2.579252000	0.859358000	-1.116287000
6	-2.357666000	-0.410756000	-0.151106000	6	-2.146697000	-0.275095000	-0.593442000
6	-4.276129000	1.286961000	-0.445484000	6	-3.661928000	1.640298000	-0.588271000
8	-5.400985000	0.956637000	-0.802054000	8	-4.834708000	1.527229000	-0.928923000
8	-3.996985000	2.499814000	0.097498000	8	-3.234011000	2.626788000	0.243712000
6	-5.110761000	3.404686000	0.246977000	6	-4.237980000	3.542244000	0.724636000
1	-5.559725000	3.624234000	-0.722273000	1	-4.715926000	4.061937000	-0.106882000
1	-5.863481000	2.982086000	0.913220000	1	-4.993608000	3.015449000	1.308701000
1	-4.688537000	4.308062000	0.680807000	1	-3.704164000	4.251039000	1.353865000
6	-2.197855000	-1.282391000	1.022833000	6	-2.589300000	-1.020971000	0.606457000
8	-2.813034000	-1.086190000	2.048209000	8	-3.393618000	-0.599115000	1.405840000
8	-1.348385000	-2.305757000	0.845255000	8	-2.016067000	-2.242923000	0.699177000
6	-1.179258000	-3.191150000	1.982251000	6	-2.419627000	-3.054315000	1.832071000
1	-0.823133000	-2.626898000	2.842930000	1	-2.157361000	-2.550128000	2.761195000
1	-2.125106000	-3.677295000	2.217932000	1	-3.492687000	-3.236571000	1.794410000
1	-0.438693000	-3.923793000	1.673423000	1	-1.870639000	-3.987157000	1.735174000

TS9				61			
$\nu_{im.}$ = -1702.5cm^{-1}(-1101.09939)				$\nu_{im.}$ = 0 cm^{-1}(-1101.19769)			
7	-2.283631000	1.599393000	-0.479420000	7	2.239846000	-1.502352000	-0.655825000
6	-1.047448000	1.894414000	-0.793327000	6	0.984499000	-1.674342000	-0.988698000
6	-2.339347000	0.210571000	-0.354787000	6	2.415721000	-0.126292000	-0.478946000
6	-1.104378000	-0.333682000	-0.595604000	6	1.239533000	0.532382000	-0.705314000
7	-0.282056000	0.754472000	-0.870070000	7	0.310864000	-0.471869000	-1.010745000
1	-0.720817000	-1.338615000	-0.579102000	1	0.942144000	1.565535000	-0.663034000
7	1.032087000	0.681414000	-1.367530000	7	-0.980522000	-0.305663000	-1.471345000
1	1.197999000	1.519489000	-1.933621000	1	-1.046156000	0.034080000	-2.424907000
1	1.478121000	-0.387788000	-1.876558000	1	-3.225927000	0.957355000	-2.042692000
7	-0.511950000	3.124872000	-1.122909000	7	0.380286000	-2.850484000	-1.355899000
1	-1.210224000	3.857500000	-1.111463000	1	0.886177000	-3.673048000	-1.057127000
1	0.298984000	3.383088000	-0.570085000	1	-0.612053000	-2.907952000	-1.167584000
6	-3.589122000	-0.475228000	0.001226000	6	3.720361000	0.429734000	-0.094273000
6	-4.776746000	0.255426000	0.159436000	6	4.828533000	-0.414970000	0.073117000
6	-3.632421000	-1.867399000	0.187362000	6	3.896938000	1.808869000	0.111728000
6	-5.969159000	-0.385508000	0.490269000	6	6.072239000	0.102201000	0.431547000
6	-4.824196000	-2.505055000	0.517517000	6	5.139256000	2.322916000	0.469384000
6	-6.000527000	-1.767904000	0.671086000	6	6.235630000	1.472512000	0.631518000
1	-4.755870000	1.328749000	0.019743000	1	4.704459000	-1.479258000	-0.081307000
1	-2.730951000	-2.459310000	0.076336000	1	3.059464000	2.487163000	-0.006607000
1	-6.875779000	0.197992000	0.607274000	1	6.915446000	-0.568629000	0.555074000
1	-4.835000000	-3.580348000	0.656841000	1	5.252643000	3.390349000	0.623183000
1	-6.928007000	-2.266139000	0.929251000	1	7.202864000	1.874633000	0.910690000
6	2.633928000	-0.804671000	-0.968760000	6	-3.135190000	0.626245000	-1.015413000
6	2.148942000	0.320250000	-0.438153000	6	-1.999191000	0.027749000	-0.599546000
6	3.820548000	-1.552761000	-0.575738000	6	-4.290002000	0.817337000	-0.142793000
8	4.925912000	-1.371776000	-1.056590000	8	-4.404273000	0.415212000	1.002732000
8	3.538462000	-2.532507000	0.302815000	8	-5.258404000	1.514245000	-0.776035000
6	4.634601000	-3.394717000	0.685348000	6	-6.462995000	1.771262000	-0.025409000
1	5.038717000	-3.904627000	-0.189353000	1	-6.932858000	0.833603000	0.272753000
1	5.420127000	-2.816816000	1.172312000	1	-6.239608000	2.367628000	0.859664000
1	4.205283000	-4.112466000	1.379638000	1	-7.111626000	2.323195000	-0.701024000
6	2.553695000	1.151913000	0.710695000	6	-1.746239000	-0.375139000	0.842958000
8	3.433398000	0.839405000	1.482350000	8	-1.372639000	0.395073000	1.691965000
8	1.843819000	2.291611000	0.796307000	8	-1.926954000	-1.684510000	1.002883000
6	2.172450000	3.168852000	1.904983000	6	-1.699134000	-2.213463000	2.335847000
1	2.023058000	2.645397000	2.848325000	1	-0.669647000	-2.026712000	2.638966000
1	3.205967000	3.502338000	1.820451000	1	-2.389141000	-1.748099000	3.038652000
1	1.489597000	4.009627000	1.821132000	1	-1.891093000	-3.279443000	2.255395000

Table S16. Optimized geometries of stationary points of the Scheme 13. PCM/B3LYP/6-311++G(d,p) calculations (Methanol, 64.7 °C). Coordinates in Angstroms. Sum of electronic and thermal Free Energies in a.u. are in parentheses.

TS10

$\nu_{im.}$ = -362.1 cm^{-1}(-1216.84814)

7	1.856643000	1.947339000	-0.718550000
6	2.799056000	1.027703000	-0.961235000
6	0.676487000	1.283048000	-0.720651000
6	0.874375000	-0.123692000	-0.867653000
7	2.265834000	-0.213447000	-1.111559000
1	0.238792000	-0.804603000	-1.421025000
7	2.922245000	-1.412341000	-1.399825000
1	3.663099000	-1.590788000	-0.727907000
1	3.304383000	-1.388522000	-2.340755000
7	4.121412000	1.282673000	-1.003138000
1	4.394134000	2.253166000	-1.043696000
1	4.743694000	0.626514000	-1.450687000
6	-0.600529000	1.972143000	-0.580684000
6	-0.646118000	3.281120000	-0.067603000
6	-1.800916000	1.353019000	-0.969419000
6	-1.859659000	3.944647000	0.062706000
6	-3.013956000	2.024564000	-0.840535000
6	-3.048602000	3.318636000	-0.322513000
1	0.276668000	3.761966000	0.231553000
1	-1.784029000	0.359952000	-1.401322000
1	-1.882642000	4.950828000	0.465605000
1	-3.931655000	1.539211000	-1.151965000
1	-3.993907000	3.840075000	-0.224098000
6	-0.798784000	-1.284734000	1.133569000
6	0.385348000	-0.934009000	0.907629000
6	-2.159377000	-1.576259000	0.964369000
8	-2.598033000	-2.468916000	0.225539000
8	-2.972858000	-0.816907000	1.736035000
6	-4.388268000	-1.065706000	1.616638000
1	-4.722812000	-0.904112000	0.591410000
1	-4.625513000	-2.085010000	1.923248000
1	-4.860912000	-0.350396000	2.285526000
6	1.636945000	-1.060610000	1.688437000
8	2.091374000	-2.113499000	2.081520000
8	2.195926000	0.131940000	1.928670000
6	3.406338000	0.131826000	2.723355000
1	3.201101000	-0.266330000	3.716863000
1	4.177117000	-0.465123000	2.236397000
1	3.709033000	1.173570000	2.783881000
1	-1.731423000	-2.498097000	-1.413290000
8	-1.263172000	-2.298020000	-2.245161000
6	-0.792722000	-3.517987000	-2.815715000
1	-1.618841000	-4.195621000	-3.059983000
1	-0.266410000	-3.268266000	-3.738203000
1	-0.096430000	-4.037022000	-2.147038000

62

$\nu_{im.}$ = 0 (-1216.87323)

7	-2.747769000	-1.074427000	0.311310000
6	-2.159747000	-2.318307000	0.310984000
6	-1.786257000	-0.199542000	0.459494000
6	-0.424048000	-0.850250000	0.611713000
7	-0.839509000	-2.262729000	0.477665000
1	-0.077371000	-0.694121000	1.639231000
7	0.052589000	-3.322576000	0.594225000
1	0.093880000	-3.863741000	-0.263954000
1	-0.180504000	-3.911278000	1.388385000
7	-2.874051000	-3.420938000	0.170609000
1	-3.873982000	-3.345538000	0.060183000
1	-2.452602000	-4.337458000	0.169584000
6	-2.044715000	1.232773000	0.518013000
6	-3.319592000	1.721806000	0.174992000
6	-1.048340000	2.136054000	0.927037000
6	-3.586014000	3.082143000	0.236133000
6	-1.324984000	3.497580000	0.993374000
6	-2.589708000	3.972635000	0.646437000
1	-4.084956000	1.025622000	-0.142813000
1	-0.063945000	1.776635000	1.196757000
1	-4.567361000	3.452824000	-0.035202000
1	-0.554259000	4.187443000	1.315479000
1	-2.800776000	5.034735000	0.695389000
6	1.894009000	-0.059312000	0.259397000
6	0.729465000	-0.428557000	-0.300160000
6	3.083226000	0.313857000	-0.456807000
8	4.018770000	-0.439820000	-0.717886000
8	3.165534000	1.660839000	-0.671914000
6	4.397406000	2.148077000	-1.232444000
1	5.241430000	1.910714000	-0.582655000
1	4.570961000	1.721075000	-2.221516000
1	4.276032000	3.226966000	-1.308120000
6	0.508570000	-0.454431000	-1.773263000
8	1.340420000	-0.188333000	-2.618530000
8	-0.757627000	-0.809930000	-2.094854000
6	-1.090372000	-0.843466000	-3.498006000
1	-0.951434000	0.141509000	-3.944239000
1	-0.467744000	-1.572509000	-4.017325000
1	-2.136442000	-1.136806000	-3.542367000
1	2.016107000	-0.078078000	2.152285000
8	1.959685000	-0.115590000	3.151111000
6	2.774856000	-1.185757000	3.610718000
1	3.833586000	-1.031653000	3.365083000
1	2.681991000	-1.239706000	4.697744000
1	2.461787000	-2.151084000	3.192319000

TS11

$\nu_{im.}$ = -1083.8cm^{-1}(-1216.86763)

7	-2.806607000	-0.839402000	0.248461000
6	-2.333618000	-2.120519000	0.412989000
6	-1.781522000	-0.036765000	0.390034000
6	-0.503524000	-0.776670000	0.703567000
7	-1.027378000	-2.154135000	0.677801000
1	-0.181845000	-0.528231000	1.731646000
7	-0.234767000	-3.263000000	0.951868000
1	-0.203177000	-3.896922000	0.159204000
1	-0.549538000	-3.740589000	1.791190000
7	-3.131210000	-3.170953000	0.325817000
1	-4.110240000	-3.025695000	0.130800000
1	-2.794518000	-4.114848000	0.441263000
6	-1.904945000	1.411176000	0.295254000
6	-3.033534000	1.973507000	-0.329375000
6	-0.920229000	2.256595000	0.835656000
6	-3.166267000	3.352193000	-0.419764000

63

$\nu_{im.}$ = 0 cm^{-1}(-1101.19909)

7	2.125459000	-2.208112000	-0.111096000
6	1.146361000	-3.081825000	0.038982000
6	1.524104000	-0.970686000	-0.185246000
6	0.143468000	-1.084201000	-0.070217000
7	-0.078162000	-2.479383000	0.041089000
1	-2.385019000	-1.267218000	-1.002472000
7	-1.286817000	-3.083845000	0.393698000
1	-1.171025000	-3.608540000	1.256290000
1	-1.603211000	-3.702296000	-0.347180000
7	1.304457000	-4.421915000	0.270347000
1	2.236398000	-4.760947000	0.075598000
1	0.578429000	-5.038307000	-0.069233000
6	2.355472000	0.220300000	-0.433616000
6	3.563874000	0.379313000	0.264904000
6	2.005131000	1.175326000	-1.397817000
6	4.386041000	1.474751000	0.019275000

6	-1.067028000	3.637364000	0.751257000
6	-2.183806000	4.186404000	0.120842000
1	-3.788119000	1.319721000	-0.747710000
1	-0.060202000	1.838979000	1.343596000
1	-4.032162000	3.780237000	-0.910774000
1	-0.311109000	4.285013000	1.178993000
1	-2.291021000	5.262845000	0.051573000
6	1.857103000	-0.152581000	0.421556000
6	0.723570000	-0.549131000	-0.177536000
6	3.138425000	0.070376000	-0.248713000
8	4.035639000	-0.754431000	-0.309141000
8	3.284951000	1.349565000	-0.658141000
6	4.565936000	1.705167000	-1.220331000
1	5.362206000	1.537578000	-0.494397000
1	4.761817000	1.122477000	-2.121083000
1	4.491255000	2.762496000	-1.463217000
6	0.628231000	-0.770389000	-1.647942000
8	1.556516000	-0.688671000	-2.426791000
8	-0.627227000	-1.069874000	-2.035101000
6	-0.841975000	-1.282651000	-3.447468000
1	-0.582956000	-0.384108000	-4.007473000
1	-0.240524000	-2.121738000	-3.797390000
1	-1.901403000	-1.503471000	-3.549162000
1	1.741335000	0.072887000	1.710788000
8	1.381545000	0.259462000	2.933689000
6	2.042197000	-0.670195000	3.747334000
1	2.679044000	-0.183215000	4.505515000
1	1.341944000	-1.324488000	4.295224000
1	2.703474000	-1.338198000	3.163505000

6	2.831474000	2.270421000	-1.644883000
6	4.022166000	2.425679000	-0.936824000
1	3.849143000	-0.360372000	1.003406000
1	1.093093000	1.052560000	-1.968850000
1	5.311584000	1.587515000	0.572818000
1	2.547772000	2.996773000	-2.398164000
1	4.664975000	3.276896000	-1.130889000
6	-2.174019000	-0.349882000	-0.474134000
6	-0.919105000	-0.105245000	-0.024803000
6	-3.330071000	0.528603000	-0.253963000
8	-3.414394000	1.435395000	0.553864000
8	-4.350135000	0.174221000	-1.067933000
6	-5.569239000	0.933755000	-0.940300000
1	-5.966401000	0.846741000	0.071588000
1	-5.390249000	1.983588000	-1.174476000
1	-6.258953000	0.497041000	-1.658263000
6	-0.590926000	1.236763000	0.601806000
8	-0.594943000	2.290653000	0.012086000
8	-0.273391000	1.093473000	1.893834000
6	0.054697000	2.304345000	2.615839000
1	0.924759000	2.783745000	2.167780000
1	-0.793754000	2.988363000	2.604556000
1	0.274249000	1.982804000	3.630394000

TS12

$\nu_{im.}$ = -418.5cm^{-1}(-1216.86865)

7	-0.493685000	-2.418416000	-0.320114000
6	0.774726000	-2.207797000	-0.613796000
6	-1.070681000	-1.172530000	-0.133218000
6	-0.134754000	-0.176880000	-0.354875000
7	1.050585000	-0.872154000	-0.638857000
1	1.206265000	2.959548000	-0.206056000
7	2.281272000	-0.249420000	-0.920556000
1	2.331992000	-0.039370000	-1.918588000
1	3.466314000	-0.942908000	-0.356732000
7	1.689440000	-3.177279000	-0.952719000
1	2.644268000	-3.011424000	-0.661732000
1	1.376922000	-4.106220000	-0.702060000
6	-2.468249000	-1.072280000	0.312604000
6	-3.426485000	-1.969840000	-0.184227000
6	-2.870962000	-0.121650000	1.262821000
6	-4.751309000	-1.901568000	0.238712000
6	-4.197488000	-0.052838000	1.683718000
6	-5.144120000	-0.940359000	1.171806000
1	-3.124572000	-2.716029000	-0.909223000
1	-2.138963000	0.549342000	1.697715000
1	-5.479218000	-2.598587000	-0.161703000
1	-4.487867000	0.685613000	2.422678000
1	-6.175426000	-0.888999000	1.502034000
6	1.139805000	1.879026000	-0.197762000
6	-0.058621000	1.266737000	-0.303224000
6	2.463253000	1.151378000	-0.206184000
8	3.469346000	1.750646000	-0.668889000
8	2.690929000	0.582834000	1.270355000
6	2.914421000	1.609735000	2.241058000
1	3.705445000	2.290150000	1.914087000
1	1.994448000	2.173261000	2.418343000
1	3.214899000	1.121431000	3.168717000
6	-1.260185000	2.144325000	-0.487621000
8	-1.407619000	3.218677000	0.056209000
8	-2.125539000	1.631154000	-1.369375000
6	-3.313982000	2.409076000	-1.638123000
1	-3.888395000	2.544212000	-0.721726000
1	-3.043060000	3.379857000	-2.053001000
1	-3.879472000	1.828225000	-2.361797000
8	4.150941000	-1.188162000	0.450581000
6	5.562930000	-0.989490000	0.137990000
1	5.833863000	-1.729547000	-0.610711000

1	5.715784000	0.020937000	-0.237911000
1	6.123576000	-1.156925000	1.054294000
1	3.735657000	-0.433144000	1.059965000

Table S17. Optimized geometries of stationary points of the Scheme 14. PCM/B3LYP/6-311++G(d,p) calculations (Methanol, 64.7 °C). Coordinates in Angstroms. Sum of electronic and thermal Free Energies in a.u. are in parentheses.

**TS13**				**64**			
$\nu_{im.}$ = -227.0 cm^{-1}(-1216.85586)				$\nu_{im.}$ = 0 (-1216.87167)			
7	0.098176000	-0.749067000	-0.433120000	7	-0.182655000	1.032520000	0.066818000
6	-0.548220000	-1.845688000	-0.814302000	6	0.184586000	2.302102000	0.342482000
6	1.410824000	-0.851409000	-0.911641000	6	-1.529630000	1.032611000	-0.367090000
6	1.531618000	-2.019776000	-1.604669000	6	-1.953573000	2.318635000	-0.326128000
7	0.290350000	-2.644020000	-1.542753000	7	-0.888423000	3.097785000	0.114275000
1	2.336435000	-2.451164000	-2.173628000	1	-2.888483000	2.771458000	-0.602882000
7	0.006642000	-3.870062000	-2.149483000	7	-0.945765000	4.489560000	0.196662000
1	-0.712693000	-3.758045000	-2.858463000	1	-0.260664000	4.911357000	-0.423955000
1	-0.292805000	-4.547020000	-1.453565000	1	-0.800841000	4.796378000	1.153482000
7	-1.811798000	-2.218783000	-0.480264000	7	1.382790000	2.711526000	0.805079000
1	-2.494624000	-1.491634000	-0.248715000	1	2.207877000	2.314905000	0.335528000
1	-2.207148000	-2.970703000	-1.027171000	1	1.461925000	3.707858000	0.961145000
6	2.435586000	0.180039000	-0.705953000	6	-2.268835000	-0.163341000	-0.792457000
6	2.099700000	1.539612000	-0.612698000	6	-1.686733000	-1.132854000	-1.623540000
6	3.791775000	-0.180224000	-0.627185000	6	-3.608926000	-0.316562000	-0.404818000
6	3.090007000	2.506273000	-0.448368000	6	-2.431231000	-2.228942000	-2.052746000
6	4.779512000	0.788693000	-0.473293000	6	-4.351449000	-1.409182000	-0.844890000
6	4.433580000	2.137828000	-0.380147000	6	-3.764293000	-2.370979000	-1.667159000
1	1.063316000	1.842240000	-0.680763000	1	-0.658066000	-1.024117000	-1.941934000
1	4.070693000	-1.226980000	-0.672298000	1	-4.062941000	0.415964000	0.252312000
1	2.809897000	3.551739000	-0.380330000	1	-1.969805000	-2.969059000	-2.696442000
1	5.819829000	0.488670000	-0.413247000	1	-5.385194000	-1.514228000	-0.536051000
1	5.202039000	2.891746000	-0.252496000	1	-4.340517000	-3.225260000	-2.003515000
6	-0.550641000	0.319725000	1.192663000	6	0.594579000	-0.157828000	0.418716000
6	-1.230385000	1.339180000	0.965944000	6	1.726060000	-0.448083000	-0.222208000
6	-0.010073000	-0.422911000	2.346839000	6	0.043683000	-0.919008000	1.581152000
8	-0.139837000	0.007613000	3.475569000	8	0.406535000	-2.033298000	1.898873000
8	0.600919000	-1.569932000	2.056882000	8	-0.875494000	-0.220205000	2.274918000
6	1.125283000	-2.312277000	3.182036000	6	-1.437499000	-0.869922000	3.435593000
1	1.867370000	-1.716396000	3.712968000	1	-1.931731000	-1.797757000	3.147315000
1	0.317872000	-2.588822000	3.859873000	1	-0.655986000	-1.080512000	4.165947000
1	1.583079000	-3.197907000	2.749765000	1	-2.157684000	-0.164081000	3.841583000
6	-1.927017000	2.250276000	0.145562000	6	2.574199000	-1.563523000	0.119424000
8	-3.095224000	2.107671000	-0.222151000	8	3.514022000	-1.522496000	0.907153000
8	-1.192953000	3.332919000	-0.175176000	8	2.333495000	-2.651993000	-0.663384000
6	-1.842193000	4.339340000	-0.985323000	6	3.227009000	-3.769314000	-0.503233000
1	-2.136083000	3.921939000	-1.948351000	1	4.254369000	-3.478959000	-0.728581000
1	-2.717625000	4.734413000	-0.469980000	1	3.175507000	-4.164724000	0.512261000
1	-1.096261000	5.118333000	-1.120471000	1	2.884663000	-4.518597000	-1.214060000
8	-4.080273000	-0.469969000	0.055836000	8	3.426261000	1.689624000	-0.981328000
6	-5.226958000	-0.704546000	-0.763896000	6	3.374683000	2.268617000	-2.284813000
1	-5.022728000	-0.484129000	-1.817525000	1	2.350211000	2.532819000	-2.573206000
1	-5.487788000	-1.759658000	-0.671787000	1	3.976368000	3.178751000	-2.269860000
1	-6.081287000	-0.104047000	-0.434369000	1	3.787945000	1.590753000	-3.039627000
1	-3.820528000	0.469731000	-0.021202000	1	2.857101000	0.869687000	-0.958858000

**TS14**			
$\nu_{im.}$ = -1149.9cm^{-1}(-1216.86918)			
7	-0.256724000	1.040493000	0.088638000
6	-0.025687000	2.347733000	0.383425000
6	-1.622903000	0.886111000	-0.261974000
6	-2.198707000	2.103553000	-0.132775000
7	-1.211935000	2.997571000	0.272070000
1	-3.199339000	2.442704000	-0.331587000
7	-1.434279000	4.369322000	0.395671000
1	-0.847131000	4.882288000	-0.256189000
1	-1.257104000	4.672801000	1.348253000
7	1.154477000	2.887274000	0.713661000
1	1.965269000	2.612598000	0.072845000
1	1.118861000	3.872610000	0.941665000
6	-2.224147000	-0.372145000	-0.721024000

6	-1.558726000	-1.213920000	-1.625727000
6	-3.515594000	-0.718283000	-0.294700000
6	-2.173640000	-2.374473000	-2.089306000
6	-4.129959000	-1.873629000	-0.770054000
6	-3.460056000	-2.707705000	-1.665668000
1	-0.568506000	-0.954853000	-1.978679000
1	-4.030745000	-0.086319000	0.419208000
1	-1.648937000	-3.013851000	-2.789896000
1	-5.127865000	-2.127960000	-0.431632000
1	-3.936342000	-3.610862000	-2.029416000
6	0.644568000	-0.058957000	0.386635000
6	1.862447000	-0.126934000	-0.154805000
6	0.154429000	-1.018783000	1.427407000
8	0.621606000	-2.125448000	1.595157000
8	-0.817377000	-0.498312000	2.191025000
6	-1.327020000	-1.339834000	3.250301000
1	-1.742435000	-2.257396000	2.834140000
1	-0.530996000	-1.579669000	3.955052000
1	-2.102995000	-0.753550000	3.735197000
6	2.837142000	-1.165974000	0.192709000
8	3.646049000	-1.076533000	1.100397000
8	2.848077000	-2.167026000	-0.711320000
6	3.855582000	-3.185682000	-0.533542000
1	4.853273000	-2.747907000	-0.577241000
1	3.718714000	-3.693861000	0.421441000
1	3.710340000	-3.881516000	-1.356347000
8	2.926021000	2.032687000	-1.071669000
6	2.581857000	2.390637000	-2.392372000
1	1.896206000	3.251831000	-2.421436000
1	3.473316000	2.660750000	-2.974212000
1	2.084997000	1.560351000	-2.920150000
1	2.418116000	0.935949000	-0.771209000

Table S18. Optimized geometries of stationary points of the Scheme 15. PCM/B3LYP/6-311++G(d,p) calculations (Methanol, 64.7 °C). Coordinates in Angstroms. Sum of electronic and thermal Free Energies in a.u. are in parentheses.

**TS15**				**65**			
$\nu_{im.}$ = -219.9 cm^{-1}(-1216.85047)				$\nu_{im.}$ = 0 (-1216.85502)			
7	1.262607000	-0.737020000	0.643969000	7	-1.641198000	-0.069440000	-0.679723000
6	0.192017000	-0.892098000	-0.094273000	6	-0.486023000	-0.613004000	-0.420785000
6	2.300251000	-0.495040000	-0.244737000	6	-2.517147000	-0.591736000	0.259617000
6	1.810935000	-0.509926000	-1.532692000	6	-1.835531000	-1.470384000	1.073751000
7	0.459902000	-0.753101000	-1.425345000	7	-0.532053000	-1.479410000	0.632187000
1	2.265792000	-0.379120000	-2.499017000	1	-2.127847000	-2.088531000	1.904695000
7	-0.413071000	-0.833787000	-2.524466000	7	0.467010000	-2.298181000	1.186537000
1	-1.052079000	-0.043286000	-2.479030000	1	1.180660000	-1.703693000	1.601196000
1	-0.955866000	-1.694403000	-2.448057000	1	0.903988000	-2.833215000	0.435182000
7	-1.105720000	-1.124708000	0.398499000	7	0.713981000	-0.361819000	-1.193809000
1	-1.057343000	-1.448108000	1.361910000	1	0.410463000	0.164081000	-2.019738000
1	-1.624180000	-1.822268000	-0.158617000	1	1.136100000	-1.273075000	-1.507772000
6	3.678045000	-0.277720000	0.217119000	6	-3.935857000	-0.209041000	0.289299000
6	4.006142000	-0.436947000	1.572421000	6	-4.464684000	0.633107000	-0.700486000
6	4.697143000	0.092016000	-0.676597000	6	-4.792908000	-0.675372000	1.299339000
6	5.310557000	-0.235116000	2.018575000	6	-5.809603000	0.996054000	-0.681085000
6	6.000082000	0.290265000	-0.229035000	6	-6.136577000	-0.312646000	1.315378000
6	6.315206000	0.127893000	1.121555000	6	-6.652950000	0.525323000	0.325285000
1	3.229572000	-0.720903000	2.271517000	1	-3.814067000	0.999491000	-1.484635000
1	4.473000000	0.232141000	-1.728059000	1	-4.411193000	-1.322573000	2.080746000
1	5.542429000	-0.363589000	3.070210000	1	-6.199342000	1.647728000	-1.455262000
1	6.771036000	0.575878000	-0.936096000	1	-6.781447000	-0.683208000	2.104327000
1	7.330042000	0.284482000	1.469007000	1	-7.699320000	0.808014000	0.339755000
6	-2.146811000	0.450987000	0.412542000	6	1.840571000	0.405687000	-0.486304000
6	-1.663734000	1.430913000	-0.210273000	6	2.903702000	-0.283549000	-0.100273000
6	-3.345854000	0.112347000	1.198329000	6	1.613382000	1.872889000	-0.436389000
8	-4.234330000	0.913230000	1.397132000	8	2.281267000	2.641217000	0.220553000
8	-3.353964000	-1.144863000	1.666687000	8	0.607986000	2.254069000	-1.245496000
6	-4.504865000	-1.536532000	2.452295000	6	0.308716000	3.668244000	-1.302792000
1	-4.582757000	-0.908350000	3.339115000	1	0.025132000	4.028939000	-0.314490000
1	-5.411657000	-1.451257000	1.854312000	1	1.175823000	4.219132000	-1.666032000
1	-4.323476000	-2.571252000	2.729597000	1	-0.522265000	3.758577000	-1.996875000

6	-1.763709000	2.742907000	-0.714345000	6	4.070979000	0.308324000	0.507531000
8	-2.257592000	3.043057000	-1.797323000	8	5.055990000	0.696554000	-0.107468000
8	-1.201587000	3.659508000	0.118485000	8	4.025751000	0.264521000	1.863307000
6	-1.214358000	5.027651000	-0.336821000	6	5.200958000	0.723669000	2.561218000
1	-0.668780000	5.125915000	-1.276068000	1	6.071876000	0.130201000	2.280294000
1	-2.237561000	5.381180000	-0.468913000	1	5.390322000	1.775784000	2.345051000
1	-0.718889000	5.594823000	0.447851000	1	4.979305000	0.591029000	3.617784000
8	-2.225361000	-3.066110000	-1.451685000	8	2.124698000	-2.703590000	-1.302768000
6	-1.931319000	-4.469672000	-1.308597000	6	2.820421000	-3.462955000	-2.300848000
1	-0.892392000	-4.541217000	-0.990318000	1	2.083351000	-4.075941000	-2.818864000
1	-2.573455000	-4.927972000	-0.552844000	1	3.311863000	-2.806842000	-3.025070000
1	-2.052814000	-4.988919000	-2.261875000	1	3.565974000	-4.115970000	-1.839346000
1	-3.142346000	-2.963611000	-1.732394000	1	2.736212000	-2.067795000	-0.837056000

**TS16**			
$\nu_{im.}$ = -1218.4cm^{-1}(-1216.85351)			
7	-1.665145000	-0.049044000	-0.683757000
6	-0.504550000	-0.572982000	-0.403902000
6	-2.548439000	-0.582908000	0.241692000
6	-1.868946000	-1.453940000	1.065567000
7	-0.557992000	-1.444161000	0.646046000
1	-2.167248000	-2.077084000	1.890672000
7	0.440920000	-2.256509000	1.210673000
1	1.070866000	-1.670599000	1.752925000
1	0.974260000	-2.691056000	0.452606000
7	0.694199000	-0.339528000	-1.177841000
1	0.398383000	0.169955000	-2.014850000
1	1.140052000	-1.308353000	-1.463528000
6	-3.972132000	-0.218384000	0.250757000
6	-4.499614000	0.609544000	-0.751780000
6	-4.835960000	-0.687873000	1.253687000
6	-5.849284000	0.955072000	-0.751958000
6	-6.184370000	-0.342811000	1.250111000
6	-6.699212000	0.480863000	0.247256000
1	-3.843835000	0.978592000	-1.530380000
1	-4.455404000	-1.323351000	2.045244000
1	-6.237626000	1.595895000	-1.535871000
1	-6.834184000	-0.715687000	2.033938000
1	-7.749263000	0.749995000	0.246535000
6	1.829669000	0.397346000	-0.495965000
6	2.919455000	-0.318407000	-0.235324000
6	1.631454000	1.856632000	-0.298105000
8	2.367732000	2.550768000	0.368343000
8	0.570258000	2.311781000	-0.978036000
6	0.290026000	3.728904000	-0.886566000
1	0.108410000	4.005675000	0.151448000
1	1.127869000	4.300758000	-1.284437000
1	-0.601562000	3.884226000	-1.487463000
6	4.146952000	0.221525000	0.341742000
8	5.080794000	0.646089000	-0.316294000
8	4.183916000	0.076319000	1.680572000
6	5.403755000	0.480673000	2.341789000
1	6.249804000	-0.093850000	1.964096000
1	5.583049000	1.545216000	2.189844000
1	5.241991000	0.270253000	3.396013000
8	2.061413000	-2.500119000	-1.231817000
6	2.743905000	-3.198250000	-2.266807000
1	2.018721000	-3.685758000	-2.924705000
1	3.354119000	-2.517281000	-2.873613000
1	3.400034000	-3.968373000	-1.846385000
1	2.708518000	-1.633217000	-0.723235000

Table S19. Optimized geometries of stationary points of the Scheme 16. PCM/B3LYP/6-311++G(d,p) calculations (Methanol, 64.7 °C). Coordinates in Angstroms. Sum of electronic and thermal Free Energies in a.u. are in parentheses.

**TS17**				**66**			
$\nu_{im.}$ = -161.3 cm^{-1}(-1216.84290)				$\nu_{im.}$ = 0 (-1216.85589)			
7	2.533873000	-1.255072000	-1.140898000	7	2.360133000	1.721061000	0.434603000
6	1.263013000	-1.360246000	-1.449212000	6	1.138601000	2.022666000	0.789215000
6	2.669619000	-0.061718000	-0.437089000	6	2.363400000	0.360942000	0.125659000

6	1.447482000	0.555811000	-0.320452000
7	0.563871000	-0.271957000	-0.995246000
1	1.122980000	1.471638000	0.142266000
7	-0.813818000	-0.074416000	-1.151956000
1	-1.095017000	-0.512232000	-2.031906000
1	-1.008140000	0.941528000	-1.222589000
7	0.630542000	-2.336691000	-2.201658000
1	1.295262000	-2.994497000	-2.590062000
1	-0.094791000	-2.830200000	-1.689308000
6	3.972411000	0.387540000	0.070283000
6	5.149932000	-0.268949000	-0.320782000
6	4.077825000	1.476990000	0.951715000
6	6.391385000	0.152413000	0.151227000
6	5.319141000	1.897794000	1.419257000
6	6.484132000	1.237725000	1.022403000
1	5.082156000	-1.110292000	-0.998839000
1	3.185112000	1.995956000	1.282308000
1	7.288790000	-0.368459000	-0.164302000
1	5.377281000	2.740078000	2.099751000
1	7.450112000	1.565021000	1.389661000
6	-3.292356000	-0.000350000	-0.329991000
6	-2.224617000	-0.551119000	0.038144000
6	-4.688809000	0.098007000	-0.089082000
8	-5.529555000	-0.698235000	-0.481882000
8	-4.994410000	1.228296000	0.598559000
6	-6.385527000	1.421990000	0.931341000
1	-7.005211000	1.379788000	0.035771000
1	-6.715738000	0.664725000	1.643679000
1	-6.437742000	2.410184000	1.382164000
6	-1.705872000	-1.476515000	1.056417000
8	-2.175961000	-1.537666000	2.171823000
8	-0.724991000	-2.269346000	0.608372000
6	-0.191730000	-3.230954000	1.554868000
1	0.217681000	-2.711534000	2.420373000
1	-0.976345000	-3.919036000	1.867073000
1	0.591522000	-3.758626000	1.017965000
8	-1.832989000	2.601785000	-1.315220000
6	-1.546446000	3.697636000	-0.431822000
1	-0.592028000	4.116818000	-0.748091000
1	-2.316927000	4.469597000	-0.504936000
1	-1.464940000	3.361975000	0.606105000
1	-2.679547000	2.199528000	-1.065340000

6	1.106236000	-0.162757000	0.287906000
7	0.330344000	0.913305000	0.702866000
1	0.677380000	-1.133933000	0.115108000
7	-0.988908000	0.828885000	1.232724000
1	-1.201078000	1.767336000	1.594896000
1	-0.992608000	0.146645000	2.052429000
7	0.637655000	3.216742000	1.281614000
1	1.378364000	3.873031000	1.496230000
1	-0.055223000	3.650957000	0.680423000
6	3.585669000	-0.316402000	-0.329557000
6	4.768840000	0.413343000	-0.519477000
6	3.603973000	-1.698193000	-0.582170000
6	5.933721000	-0.219135000	-0.949430000
6	4.767800000	-2.326906000	-1.013623000
6	5.940002000	-1.590940000	-1.199851000
1	4.766207000	1.478912000	-0.328430000
1	2.706773000	-2.289707000	-0.439335000
1	6.837836000	0.362735000	-1.090624000
1	4.760772000	-3.394454000	-1.203053000
1	6.846058000	-2.082449000	-1.535284000
6	-2.552086000	-0.857659000	0.473123000
6	-2.041255000	0.347694000	0.254209000
6	-3.608022000	-1.441242000	-0.317151000
8	-4.801585000	-1.344976000	-0.060990000
8	-3.132300000	-2.236059000	-1.308439000
6	-4.112045000	-2.965016000	-2.075335000
1	-4.688524000	-3.629567000	-1.430636000
1	-4.786105000	-2.279373000	-2.590051000
1	-3.540456000	-3.543382000	-2.797653000
6	-2.394360000	1.332889000	-0.797413000
8	-3.126474000	1.092072000	-1.730381000
8	-1.835160000	2.545099000	-0.583856000
6	-2.164842000	3.579079000	-1.546417000
1	-1.812684000	3.290431000	-2.535932000
1	-3.241798000	3.739827000	-1.564295000
1	-1.651452000	4.473367000	-1.203767000
8	-1.393651000	-1.122261000	3.038015000
6	-0.537774000	-2.146224000	3.564641000
1	0.065841000	-1.694173000	4.351135000
1	-1.131013000	-2.958166000	3.993381000
1	0.124549000	-2.549590000	2.793156000
1	-1.911195000	-1.452232000	2.258887000

TS18

$\nu_{im.}$ = -1184.1cm^{-1}(-1216.85595)

7	2.388849000	1.684526000	0.454315000
6	1.164014000	1.976981000	0.814916000
6	2.398345000	0.324164000	0.152852000
6	1.146397000	-0.206352000	0.336363000
7	0.362594000	0.864110000	0.748208000
1	0.731147000	-1.186701000	0.183801000
7	-0.947759000	0.768107000	1.289165000
1	-1.139486000	1.677803000	1.719741000
1	-1.003924000	-0.093287000	2.148341000
7	0.669291000	3.174122000	1.304814000
1	1.406032000	3.854184000	1.444917000
1	-0.079674000	3.567541000	0.744499000
6	3.620090000	-0.351908000	-0.304448000
6	4.848082000	0.327261000	-0.300764000
6	3.595521000	-1.684789000	-0.748302000
6	6.014659000	-0.308068000	-0.721752000
6	4.762006000	-2.317430000	-1.167018000
6	5.979275000	-1.632945000	-1.155971000
1	4.879075000	1.355641000	0.036405000
1	2.659996000	-2.232166000	-0.773316000
1	6.953692000	0.234311000	-0.709861000
1	4.720507000	-3.346452000	-1.506397000
1	6.886864000	-2.126608000	-1.484116000
6	-2.644507000	-0.767248000	0.560032000
6	-2.007828000	0.376605000	0.305875000
6	-3.755948000	-1.284375000	-0.223321000
8	-4.929788000	-1.047838000	0.012844000
8	-3.352099000	-2.168767000	-1.159687000
6	-4.391187000	-2.828120000	-1.915340000

1	-5.046632000	-3.392433000	-1.251336000
1	-4.975772000	-2.098778000	-2.476701000
1	-3.871045000	-3.499203000	-2.594532000
6	-2.288472000	1.327101000	-0.801912000
8	-3.011891000	1.073177000	-1.739121000
8	-1.678449000	2.517779000	-0.634644000
6	-1.929673000	3.513496000	-1.659286000
1	-1.582003000	3.148479000	-2.624820000
1	-2.994279000	3.738712000	-1.705142000
1	-1.365804000	4.391737000	-1.356630000
8	-1.464382000	-1.119229000	2.804196000
6	-0.622503000	-2.235653000	3.128006000
1	0.223553000	-1.880948000	3.718090000
1	-1.191221000	-2.957909000	3.717220000
1	-0.248518000	-2.726619000	2.224384000
1	-2.110086000	-1.276653000	1.901655000
